# Input-output signal processing plasticity of vagal motor neurons in response to cardiac ischemic injury

**DOI:** 10.1016/j.isci.2021.102143

**Published:** 2021-02-04

**Authors:** Jonathan Gorky, Alison Moss, Marina Balycheva, Rajanikanth Vadigepalli, James S. Schwaber

**Affiliations:** 1 Daniel Baugh Institute of Functional Genomics/Computational Biology, Department of Pathology, Anatomy, and Cell Biology, Thomas Jefferson University, Philadelphia, PA, USA

**Keywords:** Molecular Physiology, Neuroscience, Transcriptomics

## Abstract

Vagal stimulation is emerging as the next frontier in bioelectronic medicine to modulate peripheral organ health and treat disease. The neuronal molecular phenotypes in the dorsal motor nucleus of the vagus (DMV) remain largely unexplored, limiting the potential for harnessing the DMV plasticity for therapeutic interventions. We developed a mesoscale single-cell transcriptomics data from hundreds of DMV neurons under homeostasis and following physiological perturbations. Our results revealed that homeostatic DMV neuronal states can be organized into distinguishable input-output signal processing units. Remote ischemic preconditioning induced a distinctive shift in the neuronal states toward diminishing the role of inhibitory inputs, with concomitant changes in regulatory microRNAs miR-218a and miR-495. Chronic cardiac ischemic injury resulted in a dramatic shift in DMV neuronal states suggestive of enhanced neurosecretory function. We propose a DMV molecular network mechanism that integrates combinatorial neurotransmitter inputs from multiple brain regions and humoral signals to modulate cardiac health.

## Highlights

• Single cell transcriptomics analysis of hundreds of vagal motor neurons
• Input-output signal processing units identified from single neuron states
• Remote ischemic preconditioning and cardiac injury counter-shift neuronal states
• Putative vagal motor neuron molecular network mechanism to modulate cardiac health

## Graphical Abstract

**Figure:**
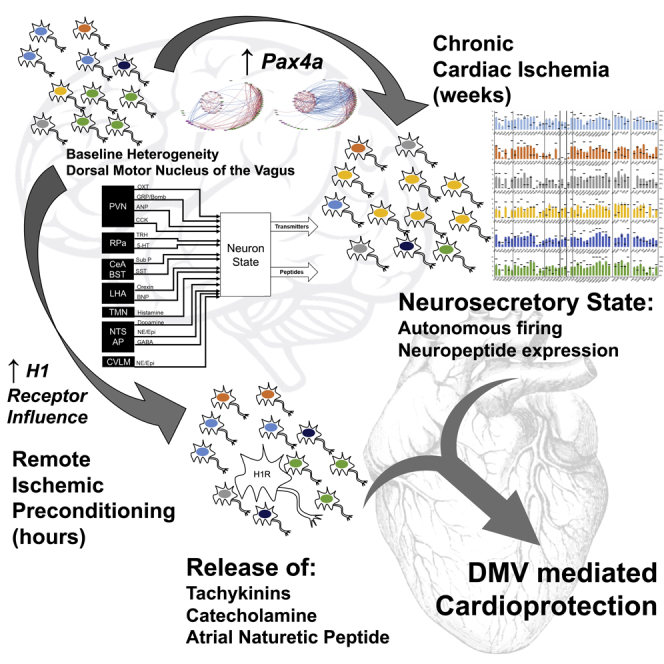

## Introduction

We aim to unravel how the dorsal motor nucleus of the vagus (DMV) responds to physiological perturbations and interacts with the periphery via the vagus nerve. It is clear that the DMV is a central modulator of homeostatic function of multiple organ systems based upon the anatomy of the projecting vagal neurons (Fox and Powley 1985; Jarvinen and Powley 1999; Cheng et al., 1999; Standish et al., 1994). The nucleus remains largely understudied as its integrative and functional capacity has been obscure, especially with regard to influence on the heart. However, recent findings suggest that DMV activity is critical to heart health and has the potential to rescue the heart from damage through its coordination of direct cardiac projections and indirect projections to the gut, which can be induced through remote ischemic preconditioning (RIPC) (Donato et al., 2013; Basalay et al., 2016; Mastitskaya et al., 2012). Traditional understanding of neuronal function suggests mediation of effector functions primarily through selective neuronal activity of one neuron type or another. Therefore, most attempts at characterizing the heterogeneity within the transcriptional landscape of neurons made the basic assumption that these neuronal subtypes were essentially fixed within the timescale of physiological perturbations, and thus a snapshot in time was sufficient to characterize them (Tasic 2018). Subsequent work has since shown shifts in these landscapes in response to development, aging, or even caloric restriction, showing that dynamic changes from one neuronal state to another were not only possible but also a matter of course in normal physiology (Polioudakis et al., 2019; Ma et al., 2020). We formulate our approach in light of these findings to examine the potential shift in DMV neuronal state in response to acute and subacute physiological perturbations including cardiac ischemic injury.

**Figure 1:**
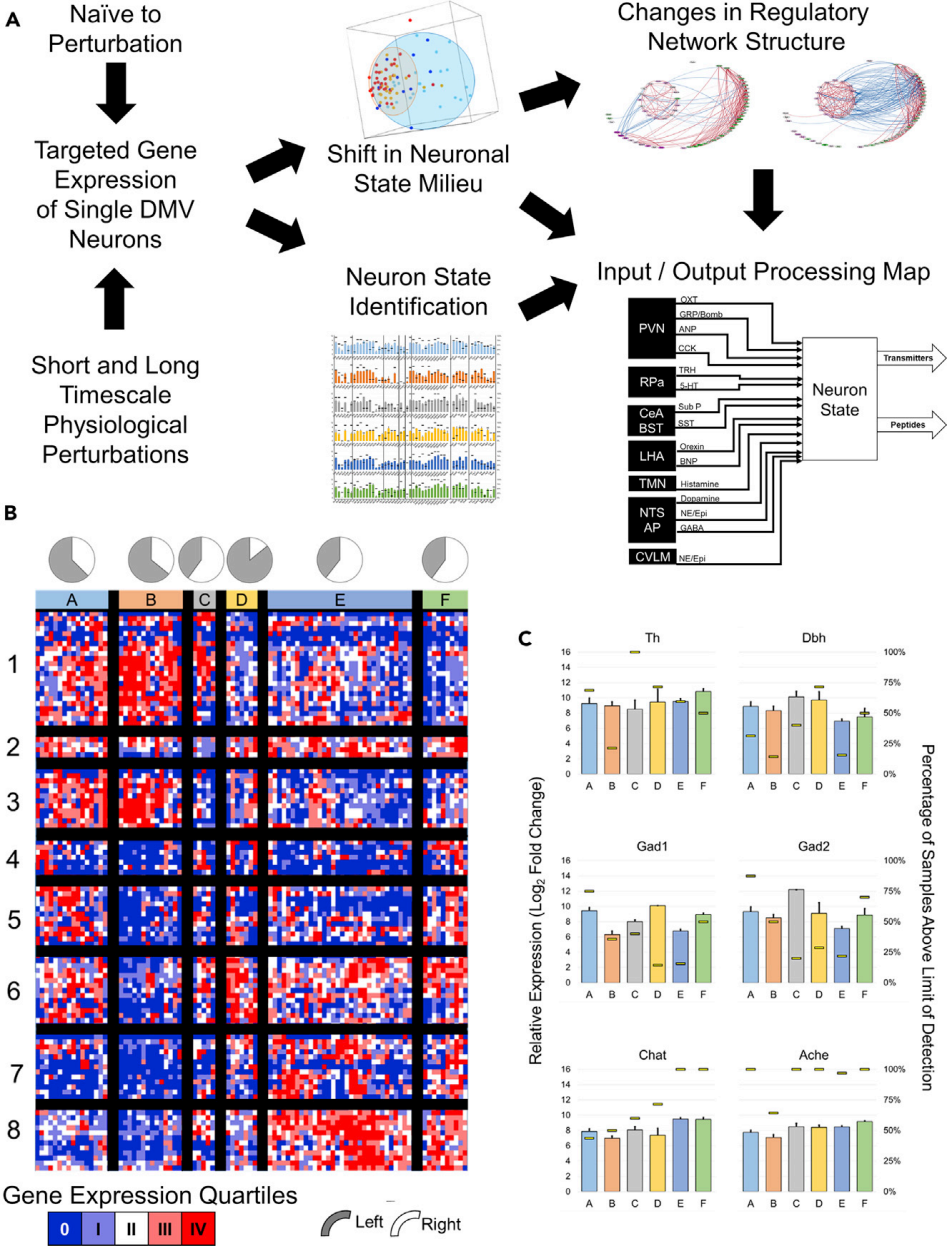
Single-cell gene expression profiling delineates distinct neuronal states in the dorsal motor nucleus of the vagus (DMV) (A) Experimental and analytical workflow to determine the DMV neuronal states and their shifting responses to physiological perturbations. The single neuron-scale gene expression data were analyzed to identify correlational neuronal clusters corresponding to distinguishable molecular states. These states, taken together with the correlated networks of receptors and neurotransmitters/neuropeptides, were used to infer state-specific input-output signal processing maps connecting DMV neuronal phenotypes to upstream brain regions as well as delineating downstream neuromodulatory systems. (B) Heatmap showing six distinct neuronal states A–F based on hierarchical clustering of single cell-scale expression of genes corresponding to neuropeptide/neurotransmitter production and neuropeptide receptors. Gene clusters 1–8 along the vertical axis are described in Table S2. Data colored to represent the quartiles of gene expression values with dark blue showing values below the limit of detection. Pie charts show the proportion of samples within each state corresponding to left versus right DMV. (C) Neuronal state-wise expression of typical neurotransmitter systems conventionally used to delineate central neuronal phenotypes. Colored bars indicate expression level relative to the limit of detection (mean ± SEM, left axis). Yellow horizontal lines show the percentage of samples with expression above the limit of detection within each state (right axis). The state-wise expression patterns of additional genes of interest are shown in the supplemental figures: neuropeptides (Figure S1), neuropeptide receptors (Figure S2), and calcium channel subunits (Figure S3).

The shifting of DMV neuronal states in response to physiological perturbations likely serves to alter a neuron's effector function (an “output”) and/or its ability to be influenced by a projection (i.e., an “input”) (Dulcis et al., 2013). Hence, a useful way to delineate neuronal states is by considering each state as representative of a particular type of signal processing unit based on combinatorial weighting of a class of inputs (receptor expression) and unique collection of a class of outputs (neurotransmitters and peptides). To investigate the distribution of such signal processing units in the DMV, we performed high-throughput microfluidic RT-qPCR of laser-captured single neurons and small pools of less than five neurons, to develop a targeted mesoscale gene expression dataset with high sensitivity, specificity, and replicability (Park et al., 2014; Achanta et al., 2020). In each single cell-scale sample, we profiled a large panel of neuronally relevant genes including signaling pathways, high-yield receptors, neurotransmitter enzymes, and neuropeptides collated from a wide-ranging survey of the literature on DMV gene and protein expression, and neuronal connectivity (Table S1). We sought to analyze the data for gene expression modules and gene coexpression correlation networks and organized the results into distinct input-output signal processing units that represent the potential interaction strength of inputs from several brain regions to the DMV and the combinatorial effector molecules as putative outputs of these neuronal states (Figure 1A). We examined the potential shift in these DMV neuronal states in response to acute physiological perturbation of RIPC, whose effects on the heart occur within hours and require alteration of DMV neuronal activity (Mastitskaya et al., 2012; Basalay et al. 2012, 2016; Donato et al., 2013). In addition, we induced longer timescale dynamic changes in the DMV neuronal landscape through ligation of the left anterior descending (LAD) coronary artery in a chronic myocardial ischemia model over the course of 3 weeks. The observed changes in the distribution of neuronal states are representative of alterations in the input/output signal processing function of the nucleus as a whole and specific to each physiological perturbation. Our proposed framework to analyze DMV neuronal state landscape as a set of input-output signal processing units affecting neuromodulatory action of vagal outflow offers new insight into how the DMV accomplishes its autonomic regulatory function by dynamically responding to the demands of the organismal physiology.

## Results

### Characterization of homeostatic molecular states of DMV neurons

**Figure 2:**
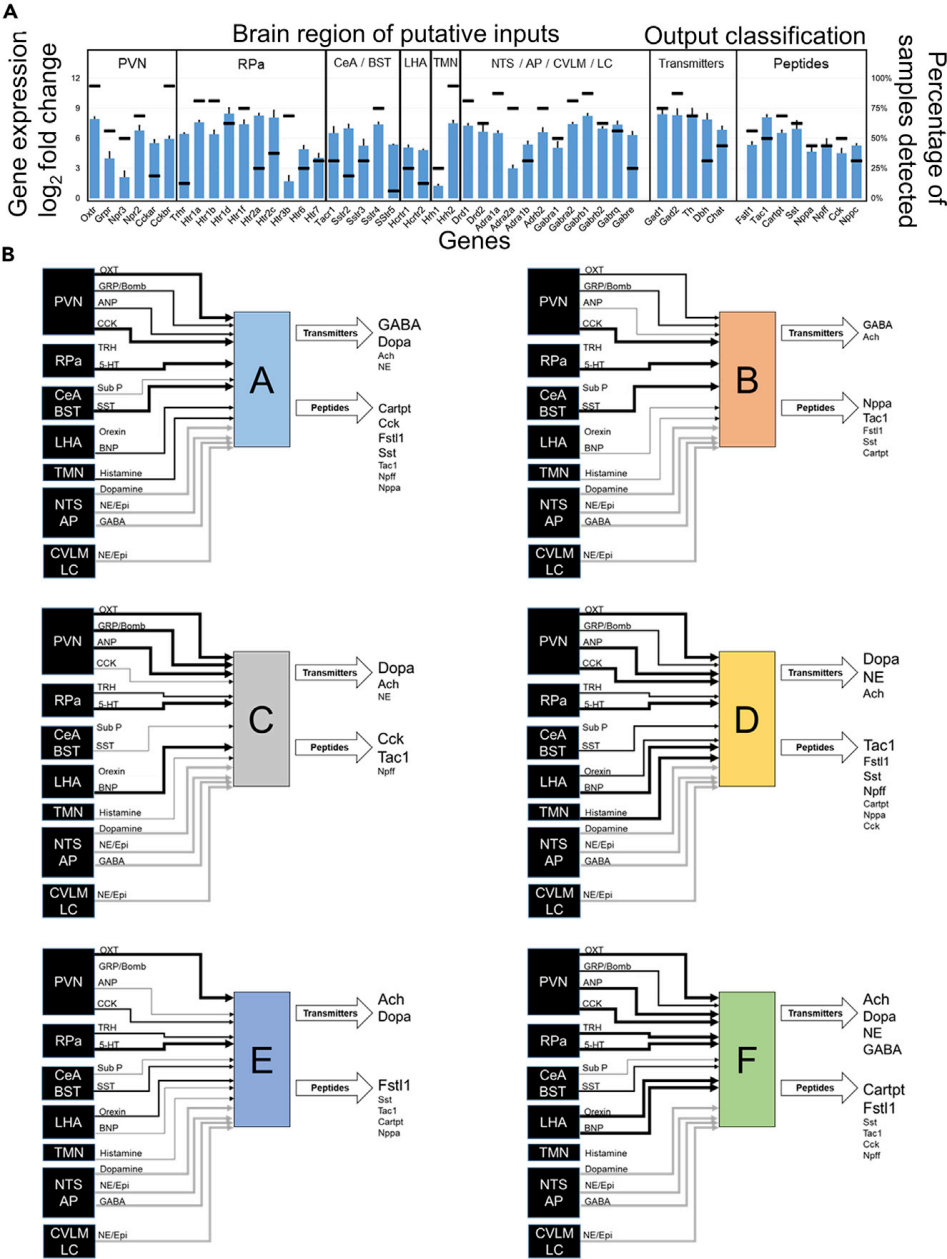
Input-output signal processing map of DMV neuronal states (A) Gene expression profile of DMV neurons in state A indicating the levels of a range of receptors linking these neurons to other brain regions (input signals) and the neurotransmitter/neuropeptide systems expressed in these neurons (output signals). Several brain regions projecting onto DMV neurons utilize specific neuropeptidergic systems, enabling inference of input connectivity and putative interaction strength based on receptor expression level within DMV. Detailed methods for network inference and supporting literature are included in the Transparent methods. Bar graph corresponds to the expression level relative to the limit of detection (mean ± SEM, left axis), and the horizontal lines correspond to the percentage of samples above the limit of detection for each gene (right axis). The input-output gene expression profiles of other states B–F are shown in Figure S4. (B) Network representations of the input-output signal processing map of DMV neuronal states. The thickness of lines connecting the brain regions to a specific DMV neuronal state is proportional to the gene expression level of the receptors corresponding to the neuropeptide inputs, signifying putative interaction strength and information flow from these brain regions into DMV. The neurotransmitter/neuropeptide output signals from the DMV neurons are indicated in varying text size proportional to the gene expression level of corresponding enzymes. Within each DMV neuronal state-specific map, the input and output signals corresponding to the genes with expression below the limit of detection in those neurons are not shown. The regions NTS, AP, CVLM, and LC are grouped together as the corresponding inputs through dopamine, GABA, and norepinephrine cannot be specifically assigned to one of the regions. These inputs are shown in gray for all states. PVN, paraventricular nucleus; Rpa, raphe pallidus; CeA, central amygdala; BST, bed nucleus of the stria terminalis; LHA, lateral hypothalamus; NTS, nucleus tractus solitarius; TMN, tuberomammillary nucleus; AP, area postrema; CVLM, caudal ventrolateral medulla; LC, locus coeruleus; Ach, acetylcholine; Dopa, dopamine; NE, norepinephrine (noradrenaline); Epi, epinephrine (adrenaline).

**Figure 3:**
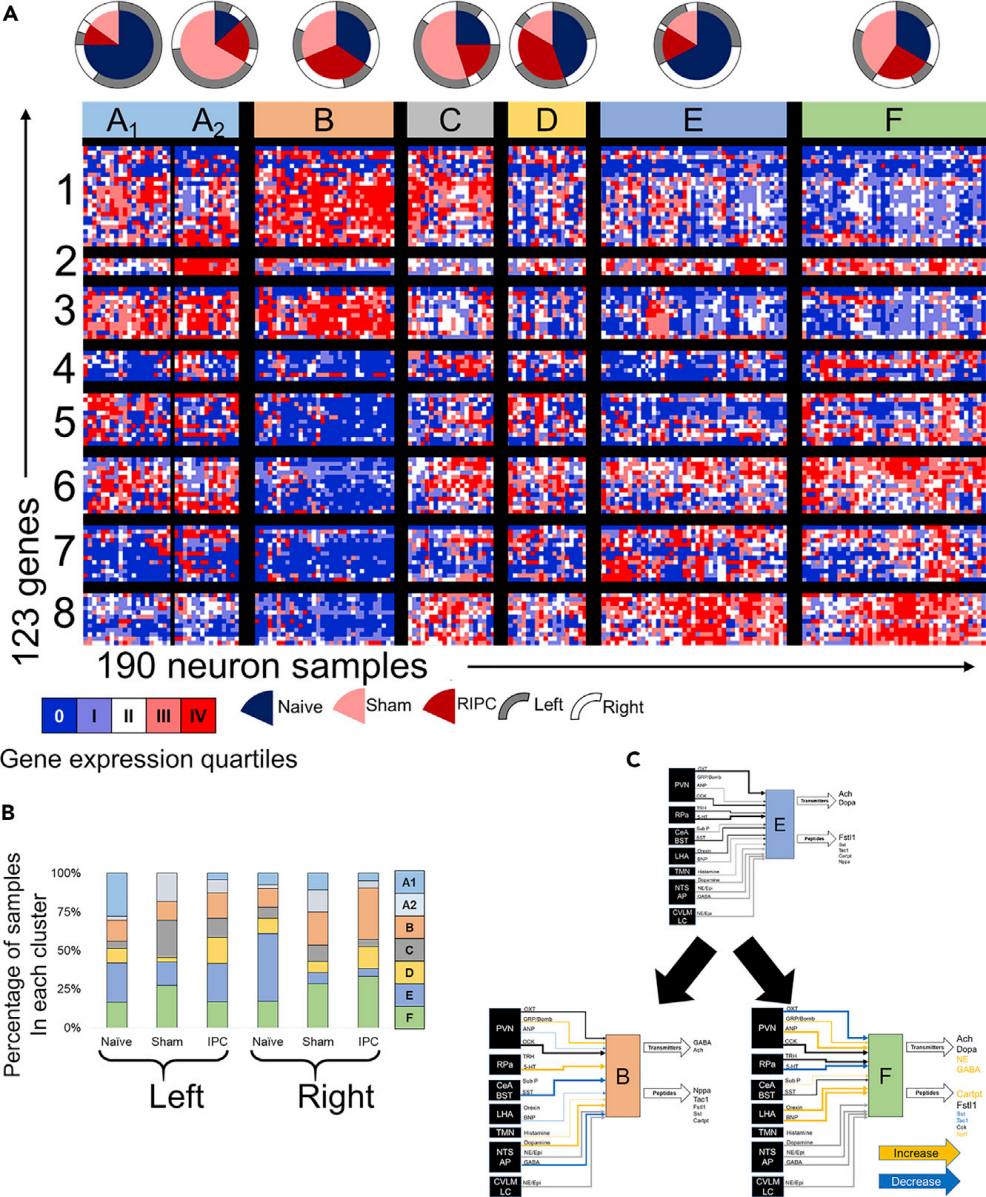
Physiological perturbations such as remote ischemic preconditioning (RIPC) and sham surgery shift the distribution of DMV neuronal states 2.5 h (A) Heatmap of single neuron gene expression showing the distribution of DMV states in homeostatic (naive), sham surgical, and RIPC conditions. The proportion of neurons from each experimental condition is indicated in the pie charts. The border of the pie charts corresponds to the proportion of cells from left versus right DMV. (B) Distribution of neuronal states on the left and right sides of the DMV in the naive, sham, and RIPC conditions. (C) Distinct neuronal states are enhanced in the right DMV after RIPC versus naive, evidenced by diminished state E with enhancement of states B and F. The physiological perturbation-induced changes in the gene expression of receptors (inputs) and neurotransmitter/neuropeptide systems (outputs) are mapped onto the state-specific networks as putative alteration of the interaction strength of input information flow or output signals: orange (increasing), black (unchanged), and blue (decreasing). PVN, paraventricular nucleus; Rpa, raphe pallidus; CeA, central amygdala; BST, bed nucleus of the stria terminalis; LHA, lateral hypothalamus; NTS, nucleus tractus solitarius; TMN, tuberomammillary nucleus; AP, area postrema; CVLM, caudal ventrolateral medulla; LC, locus coeruleus; Ach, acetylcholine; Dopa, dopamine; NE, norepinephrine (noradrenaline); Epi, epinephrine (adrenaline).

We started with delineation of DMV neuronal states in the homeostatic state devoid of any specific physiological perturbations. We obtained a single neuron-scale dataset from 178 neurons, and assayed 169 genes in each sample. Hierarchical clustering of quartile binned data yielded six transcriptional phenotypes (Figure 1B; Table S2 contains the details of the associated gene modules). These six DMV neuronal states were not distributed along the lines of canonical neurotransmitter production, as several neurotransmitter systems were abundantly expressed in multiple neuronal states (Figures 1C, 2, and 3). However, peptides (Figure S1), peptide receptor subtypes (Figure S2), and ion channels (calcium channels highlighted in Figure S3) showed differential expression across the six DMV neuronal states, suggesting differential neuromodulatory functions (see the section *Explanation of bar graphs for gene expression used throughout the manuscript* in Supplemental information*,* for further explanations of data representation).

We analyzed the six homeostatic neuronal states to develop a signature set of genes that are informative of the neuromodulatory signal processing within each state. We assessed a wide range of literature to construct an input-output signal processing structure onto which we mapped the differential gene expression data to infer distinctive neuromodulatory functions of DMV neurons (Supplemental Text). In this scheme, the relative differential expression pattern of genes corresponding to the processing of afferent signals (e.g., receptors) and those of effector functions (e.g., neurotransmitters) indicates the input-output signal processing likely to occur in the DMV neurons in a given state (Figures 2A and S4). We represented the gene expression signatures corresponding to the six neuronal states (Figure 1B) as distinguishable input-output signal processing units (Figure 2B). Neuronal states A and F mainly correspond to the GABAergic phenotypes (Figures 2B and S5–S8) with state A including mostly *Gad*+ subtype G_A with a little bit of G_B and F including subtypes G_C and G_D. In neuronal states B and E there is a small cohort of *Gad+* neurons that share some characteristics of G_B, but not all (e.g., expression of *Chat*). Further examination of Gad + neurons in the DMV and their selected subtypes defined as G_A through G_D can be found in the Supplemental Text.

State B neurons have the highest expression levels of peptide receptors as well as most of the serotonin, dopamine, and histamine receptors. There were two distinct sub-states with a notable difference in the expression of *Gad1* and *Gad2*. It is possible that given the diversity of more highly expressed receptors, these neurons play an integrative role taking a wide diversity of input. The production of several peptides in the relative absence of the neurotransmitter enzymes assayed here are suggestive of a peptidergic neurosecretory phenotype of neurons in state B.

Neuronal state E represents what may be considered a canonical efferent motor neuron from the DMV. These neurons express a combination of channels consistent with the observations by Goldberg et al. (2012) and Cooper et al. (2015) and have the requisite *Chat* and *Ache* expression corresponding to cholinergic neurons. These neurons also highly express the more standard GABAA and GABAB receptor subunits, in line with observations that a subset of DMV motor neurons was especially sensitive to GABAergic signaling (Cauley et al., 2015). It is notable that neuronal state E constitutes a greater proportion of samples than any other of the distinguishable states. The relative lack of other receptors and the ion channels related to firing canonical action potentials rather than tonic or burst pacemaking (HCN and low-voltage calcium channels) suggests that the unipolar neurons described in the monosynaptic gastric reflex circuits by Rinaman et al. may be primarily of this phenotype (Rinaman et al., 1989).

Neuronal states C and D are most notable for their dominant expression of either gene group 1 or gene group 6 (Table S2). Both clusters are made up of a relatively small number of samples, but have distinct gene expression patterns compared with nearest neighbor states B and E. State C neurons expressed much of the same canonical satiety peptides and neurotransmitter receptors as that of state B neurons, but without inputs derived from the same peptidergic neurons. The receptor expression profile in state B neurons suggests a greater interaction strength to respond to opioids, somatostatin, and cytokines like leukotriene B4 and IL-6 inputs. Neuronal state D is similar to state E, but with a more distinct upregulation of genes in group 6, whose membership includes multiple genes associated with pacemaker-like behavior (Hcn2, Girk2). Also of note is the distinct upregulation of Hrh3 coding for the histamine 3 receptor, which is a modulatory hub of satiety in the rat (Attoub et al., 2001).

### DMV neurons show specific state shifts in response to surgery and remote ischemic preconditioning

We obtained a comparable single neuron gene expression profiling dataset on DMV neurons after RIPC (Figure 3A). We compared the shift in DMV neuronal state due to RIPC to that in the homeostatic condition as well as due to sham surgery. Surprisingly, sham surgery had a prominent effect on gene expression in DMV neurons, even after a mere 2.5 h. Several clusters differed from baseline in their sample distribution, most prominently the states A2, C, and D (Figure 3A). Both A2 and C have an over-representation of sham samples, whereas D has an over-representation of RIPC samples. The main differences from A1 to A2 can be found in parts of gene module 1, 2, and 7 (Table S2). The differences between the otherwise similar neuronal states C and D can be found mostly in gene cluster 1, which contains genes involved in sensing peptide and canonical neurotransmitters as well as key intracardiac neuron effectors like ANP and tachykinins as discussed later. These results indicate that neuronal response to RIPC leads to a DMV-wide gene expression pattern more similar to homeostatic naive samples rather than sham surgery, suggesting a RIPC-induced repression of the stress response seen in sham.

In addition, the effect of the surgical treatment (RIPC or sham versus naive control) was significant in the *Fos*+ neuronal subset (Supplemental Text, Figure S9). The most prominent shift was the significant upregulation of *Th* in DMV neurons from the RIPC and sham cohorts (ANOVA, p < 0.001), suggesting that DMV neurons can act like sympathetic neurons in an acute stress response.

A subset of DMV neurons appear to respond to RIPC or sham surgery by shifting to another state characterized by altered input-output signal processing structure reflected in the gene expression profile. This occurred in the sham condition most prominently on the left side of the DMV, where state A_1_ (the same as state A in Figure 1B) shifted to state A_2_ (Figure 3B). The state transition of DMV neurons within the six signal processing structures delineated in Figure 2B was readily apparent in the RIPC condition, particularly on the right side of the DMV where the decrease in proportion of neuronal state E coincided with an increase in the proportion of states B and F (Figure 3B). We inferred the functional implications of such a shift in neuronal states by mapping the gene expression profiles to the input signal processing that is likely to be dialed up or down and which output effector functions are enhanced or suppressed. Our results suggest that following RIPC, the processing of inhibitory signals from the NTS (nucleus tractus solitarius) and CeA (central amygdala) are likely diminished, while at the same time amplifying the processing of excitatory signals from the RPa leading to an increased effect of natriuretic peptide A and tachykinins on the DMV through enhanced neuronal state B (Figure 3C). By contrast, the sham surgery-induced shift toward neuronal state F represents a potential amplification of response to excitatory inputs from LHA and paraventricular nucleus (PVN) via a canonical sympathetic combination of neurotransmitter signals (NE and Cartpt) (Figure 3C).

The RIPC-specific effects on DMV neuronal gene expression were subtle with the most notable change being a shift in histamine receptor subtypes (Figures S10 and S11) with no notable shifts in housekeeping genes (Figure S12), suggesting increased interaction strength from the TMN. Gabra2 (gamma-aminobutyric acid receptor subunit alpha-2) and Adra1a (adrenergic alpha-1 receptor) have a uniquely lower expression after RIPC, suggesting that RIPC diminishes inhibitory input signal processing mediated by GABA or NE (Figure S10B). In addition, there is a reduction in expression of *Npr2* (signal transducer of natriuretic peptide) and up-regulation of *Npr3* (clearance receptor for the natriuretic peptide) in the DMV neurons (Figure S10B). This shift in balance suggests a reduced influence of natriuretic peptide inputs from PVN to DMV, or alternatively, decreased influence from inhibitory interneurons producing natriuretic peptide within DMV. Also notable in the RIPC-specific response is a decrease in the number of DMV neurons expressing *Fstl1*. The cardioprotective peptide follistatin-1 can potentially be released via projections from DMV onto cardiac ganglia (Ogura et al., 2012; Wei et al., 2015) and is known to suppress afferent transmission by potentiating Na+/K + ATPase in neurons and myocytes (Li et al., 2011). Our results suggest that the cardioprotection by RIPC reported by physiological studies (Mastitskaya et al. 2012, 2016; Basalay et al., 2016) may not involve follistatin-1-mediated processes stimulated by vagal efferents originating from DMV.

### DMV microRNA regulatory network changes in response to cardiac ischemic injury and RIPC-mediated cardioprotection

**Figure 4:**
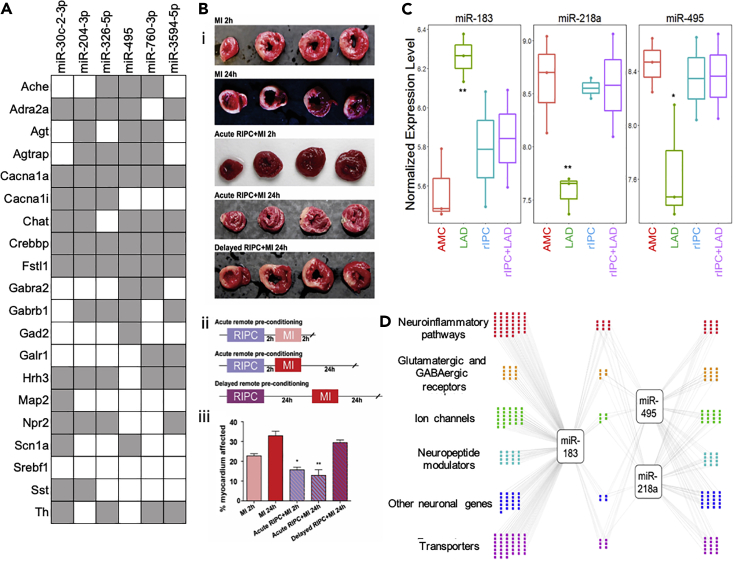
Likely microRNA mediators of DMV neuronal gene expression changes from RIPC (A) Putative regulatory interaction matrix microRNA regulators RIPC-downregulated target genes. Although the significant genes that were discussed in this work are present, several other genes with the same expression pattern that did not meet the cutoff for statistical significance were also included in the query. Putative binding was determined through the use of several algorithms (mirWalk, RNA22, miRanda, and Targetscan) and a “hit” taken when more than one of these was determined there to be a putative binding site in the 3′UTR. (B) Effects of remote ischemic preconditioning (RIPC) on the extent of myocardial tissue damage are present after 2 h and subside by 24 h. (i) Cardiac tissue after each treatment combination stained with triphenyl tetrazolium chloride (TTC) with areas of infarct shown in white and viable myocardium in red. (ii) Timeline of each RIPC experimental treatment group. The MI 2h and MI 24h groups did not have an RIPC component and only included induction of MI with the respective waiting time to tissue collection. (iii) Quantitative analysis of infarct size expressed as percentage of left ventricle (mean ± SEM). N = 3–5 per group. ∗p < 0.05, ∗∗p < 0.005. (C) Putative microRNA control points in the DMV that showed dysregulation by LAD and normalization by RIPC. ∗∗p < 0.005; ∗p < 0.05, compared with age-matched control. We tested for expression and differential regulation of ~400 microRNAs using Nanostring digital counting platform. A total of 146 microRNAs were detected (Figure S13A). Template analysis identified a subset of microRNAs that showed dysregulation in LAD and normalization by RIPC (Figure S13B). Of these, a set of three microRNAs showed >2-fold differential regulation due to LAD compared with RIPC. (D) Differentially expressed microRNAs target genes in processes relevant to neuronal plasticity and neuroinflammatory processes. We used miRWalk to predict genes relevant to neuronal processes that are putative targets of the three high-priority differentially regulated microRNAs. We filtered miRWalk predictions for those expressed in the DMV using available RNA-seq data from rat models of autonomic dysfunction. Colored dots represent the target genes categorized based on gene ontology and functional annotation.

With coordinate gene expression changes, it may be possible to determine likely effectors, such as microRNAs. Assaying microRNA changes in the DMV during LAD ligation with and without RIPC through find several that shift significantly in RIPC or that are renormalized by it (Figures 4, S13, and S14). Based on the p value from template matching, fold change between LAD and controls, and overall abundance, three microRNAs were identified as potential candidates for therapeutic intervention, miR-218a, miR-495, and miR-183. Of these three microRNAs, miR-495 has been shown to have strong cell type specificity, being heavily enriched in neurons compared with astrocytes, oligodendrocytes, and microglia (Jovičić et al., 2013). Of the top 10 microRNAs that were determined to most likely affect the genes examined that were down regulated in RIPC (Figure 4A), miR-495 was shown to actually have differential regulation during LAD ligation and be returned to normal levels with RIPC before LAD. Further examination of the gene network that these three microRNAs may be regulating was carried out using the miRWalk database. Genes with the highest binding prediction were taken into consideration. Genes that did not have robust expression in the DMV were filtered out using available RNA sequencing (RNA-seq) data in the brainstem. The remaining network represents the important genes involved in inflammatory and immune processes, excitatory and inhibitory receptors, ion channels, neuronal peptides and regulators, as well as transporters that are targeted by miRs-218a, 495, and 183 (Figure 4D).

### Effects of persistent cardiac ischemia on transcriptional state

**Figure 5:**
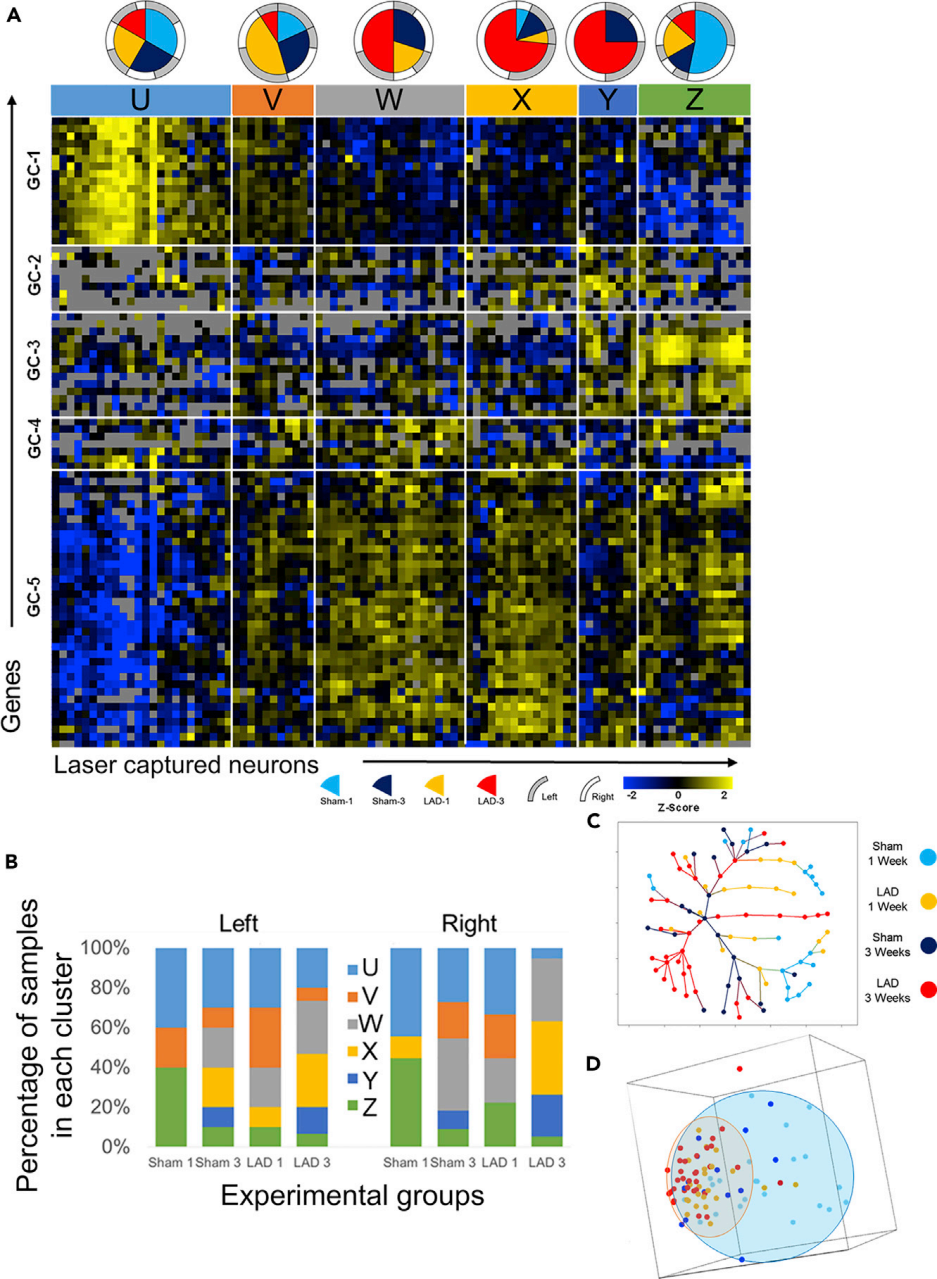
Chronic cardiac ischemia shifts the neuronal states of DMV neurons with distinct distributions at 1 and 3 weeks (A) Heatmap showing gene expression (*Z* score of -ΔC_t_) organized by hierarchical clustering of both genes and samples as well as composition of sample cluster (pie charts). Each column represents one sample (pool of ~5 neurons collected from only one side of the DMV). Each row represents a gene assayed through multiplex RT-qPCR. Sample clusters are colored for clarity of reference in subsequent figures. Gray boxes indicate that no data is available for that particular reaction. Experimental design regarding sample clusters can be found in Table S3, and annotations for gene clusters can be found in Table S4. (B) Distribution of neuronal states on the left and right sides of the DMV in the sham and LAD treatment groups. (C and D) Dimensionality reduction through minimum spanning tree (C) and principal-component analysis (D) demonstrates diminished expression space for LAD-3 samples toward the neuronal states W, X, and Y expression patterns. Minimum spanning tree colored for experimental groups shows the unique terminal branches of LAD-3 composed of samples of neuronal states X and Y. The same MST colored for neuronal state is shown in Figure S16. (D) Plot of first three principal components with ellipsoids showing the general expression space for the sham samples (blue) and the LAD samples (orange) regardless of time point. Points are colored according to the experimental group. This plot demonstrates a reduction in the expression space (diversity) of LAD samples toward an expression pattern that is present in some of the sham samples.

To investigate DMV homeostatic responses on a longer timescale, ligation of the proximal LAD artery was performed with survival times of 1 or 3 weeks to examine early dynamics of DMV responses to persistent ischemia (Table S3). Similar approaches of assaying gene expression were used as with the RIPC studies. Through the use of a similar hierarchical clustering algorithm with average linkage of Pearson correlations, it is possible to identify six neuronal states and five gene clusters (GC) through dendritic tree height cutoffs that parsimoniously segregate groups. This provides the framework for understanding the heterogeneous responses to cardiac ischemia over time (Figure 5A, Table S4). The distribution of samples from experimental groups among these six neuronal states is not uniform as would be expected if LAD ligation had limited effect on the behavior of the DMV as a whole. Instead, a clear increase in the representation of certain states with specific conditions and in many cases, distinct distributions of phenotypes on the left and right is observed (Figures 5B and S15). The 1-week sham (Sham-1) samples are generally divided into three main states, State-U, State-V, and State-Z. The 1-week LAD ligation samples (LAD-1) are distributed similarly, but with the additional representation in State-W. The sham 3-week samples (Sham-3) are represented in all single neuron states with negligible left/right differences. The 3-week LAD samples (LAD-3) distribute much differently than all the other groups; State-U and State-Z have only sparse representation, whereas State-W, State-X, and State-Y are heavily represented, an effect that is exaggerated on the right side when compared with the left (Figure 5B). Details of the gene order in the heatmap and sample composition can be found in Table S3.

The distinguishing features of State-U are high expression of GC-1 and low expression of GC-5 with average levels of the others. State-V may be considered as a transition from State-U to State-W with expression levels of nearly all the GC in between those of State-U and State-W. State-W has a characteristic high expression of GC-4 and GC-5 together and lower expression of GC-1 and GC-2. State-X has a high expression of GC-2 and GC-5 with more modest upregulation of GC-4 and downregulation of GC-3. State-Y includes upregulation of GC-2, GC-3, and GC-4 together. State-Z has a marked downregulation of GC-1 and marked upregulation of GC-3 with modest upregulation of GC-5. Although not exact opposites, the composition of State-U and State-Z has gene clusters (GC-1, GC-3, and GC-5) that have opposing expression tendencies (Figures S16 and S17).

The two sample clusters at the opposite ends of the heatmap in Figure 5A, State-U and State-Z, are the most clearly defined distinct transcriptional phenotypes based upon their average Euclidean distances in the minimum spanning tree (MST) (Figures 5C and S16). Of note as well is that both State-U and State-Z are composed of samples from all the experimental groups, albeit in different proportions, perhaps suggesting their ubiquity in DMV function, irrespective of cardiovascular perturbation. Clusters State-V, State-W, and State-X may initially all appear to be transitional groups that represent the populations of neurons that are in the process of shifting from State-U to State-Z, or vice versa. However, an examination of the MST in Figure 5C suggests that State-V and State-W are more likely transitional clusters, whereas State-X and State-Y are terminal phenotypes. The branching pattern for State-Y in the MST suggests that it is really two separate phenotypes that in this case differ due to ischemic cardiac damage (Figure S16). There is one branch that is composed entirely of LAD-3 samples and has a distance of 3–4 in the MST from the bulk of the two main phenotypes, State-U or State-Z (Figure S16). The other State-Y samples are from the Sham-3 group and are adjacent to the rest of the samples from State-Z (Figure S16).

A more broad interpretation, considering all genes examined here, suggests that State-U and State-Z are the states of DMV neurons that exist either at resting state or are recruited for generalized injury/stress response. This is shown in the distribution of sample clusters within experimental groups in Figure 5B. The combination of nearly equal representation of all experimental groups for each and the distinct gene expression patterns support this. Although the heatmap makes State-W and State-X appear very close in expression patterns, examination of the spanning tree suggests that State-X is more of a terminal state, whereas State-W represents the transitional state. Given the poorly defined separation of these clusters, it is possible that they are a similar state, but are behaving in different ways, perhaps a result of inputs or feedback, but are operating under a similar overall coordinated program. The main difference between State-W and State-X is the higher expression of GC-2 in State-X, a gene module that contains neuropeptides like somatostatin and galanin along with the somatostatin-3 and the oxytocin receptors. It also has a clear upregulation of the beta-1 adrenergic receptor, better known for its cardiac specificity in the periphery, and for which little is known about function in neurons of vagal motor neurons. Another distinction includes the upregulation of GC-4 in State-W compared with State-X. GC-4 contains several inhibitory receptors: two GABA receptor subunits (ε and θ), two G_I_ -coupled muscarinic receptors, and the G_I_ -coupled alpha 2 adrenergic receptor. When considered along with the relatively increased expression of several calcium channel genes (except Cacna1c) (Figure S17), this is suggestive of a neuronal state capable of pacemaker or even burst firing (Cui et al., 2018; Yang et al., 2018; Pape and McCormick 1989; Zhu et al., 1999). Also contributory to this conjecture is the upregulation of Grin2a, Hcn2, and Kcnn4, all of which are involved in autonomous firing (Dong et al., 2016; Chan et al., 2004; Yang et al., 2018). Along with the unique inhibitory receptors from GC-4 there is also increased expression of Gabra1 and Gabrb1 GABA receptor subunits, which may be necessary to lower the resting membrane potential of these neurons or prevent large depolarization events to permit their function as autonomous pacemakers capable of burst firing (Cui et al., 2018; Yang et al., 2018; Zhu et al., 1999). Also of interest in State-W and State-X is the increased expression of several receptors that are responsive to two somatostatin receptor subtypes and both orexin receptor subtypes. This may suggest an increased interaction strength with the central nucleus of the amygdala and lateral hypothalamus, respectively, as the sources of somatostatin and orexins as neurotransmitters (discussed in Supplemental Text).

Consideration of the expression pattern across all measured genes using principal-component analysis (Figure 5D) yields two interesting observations. The first is that the expression space, diversity of expression pattern, for the LAD ligation samples occupies a much more constrained space (red ellipsoid) than the sham samples (blue ellipsoid). The second observation of note is that this constrained space shown by nearly all LAD samples from both time points lies within the expression space defined by the sham samples. This suggests that there is programmatic coordination in response to ischemic injury and that this response is within the realm of normal expression patterns at least for some neurons. In this case the response phenotypes include State-V and State-W for LAD-1 and State-W, State-X, and State-Y for LAD-3.

In the consideration of gene expression correlation networks, the LAD-3 group has much lower connectivity than either of the 1-week groups and also less unique edges. Most surprising is the emergence of Pax4a as a unique hub gene in its relationship to many GC-5 genes (Figure S18). These networks also reveal an early response to LAD at 1 week that is uniquely and significantly coordinated as evidenced by the increased connectivity of the gene expression networks between the oppositely expressed GC-1 and GC-5. More detailed explanations, implications, and figures can be found in the Supplemental Text and Figures S18–S21.

### Effects of persistent cardiac ischemia on gene co-expression network topology

Gene co-expression networks were generated for each of the experimental groups using Pearson correlations and filtered using a q value cutoff of q < 10^−3^. The unique edges for each of the networks generated are given in Figure S18 with the connectivity in any of the experimental groups being excluded from the full network for each group given in Figures S18–S21. Genes with no connectivity in any of the networks based upon the cutoff criteria mentioned previously were excluded from all the network figures. It is clear from these networks that the LAD-1 group has the greatest overall connectivity with over half of all edges being unique to that group. Many of these unique edges are the negative correlation between genes in GC-1 and GC-5 (Figure S18). Also of note are unique edges of interconnectivity within GC-1 and GC-5, respectively. The notable unique edges of the sham 1-week group include several negative correlations with Foxo4, Camk2, and Drd4 in GC-3 with genes in GC-1 (Figures S19 and S20). Also, there are several unique positive correlations to genes in GC-5 from other GCs as well as some unique interconnectivity within GC-5. The Sham-3 group is most notable for its very sparse connectivity (Figure S20).

Within each of the treatment cohorts, there are several highly connected genes as is the baseline expectation for a biological network, suspected to have scale-free topology (Barabási 2009). Overall, a few genes are highly connected across most of the cohorts, most notable being Cacna1b that is a member of the top 5 connected genes in each cohort (Figure S17). Cebpd is highly connected in the LAD-1 group and the LAD-3 group, but with many unique edges in the LAD-1 group (Figure S18). This suggests a role of Cepbd in mediating gene expression in the acute response to cardiovascular injury that persists to some extent beyond the acute phase.

### Putative DMV neuromodulatory network mechanism mediating RIPC-induced cardioprotective effects

**Figure 6:**
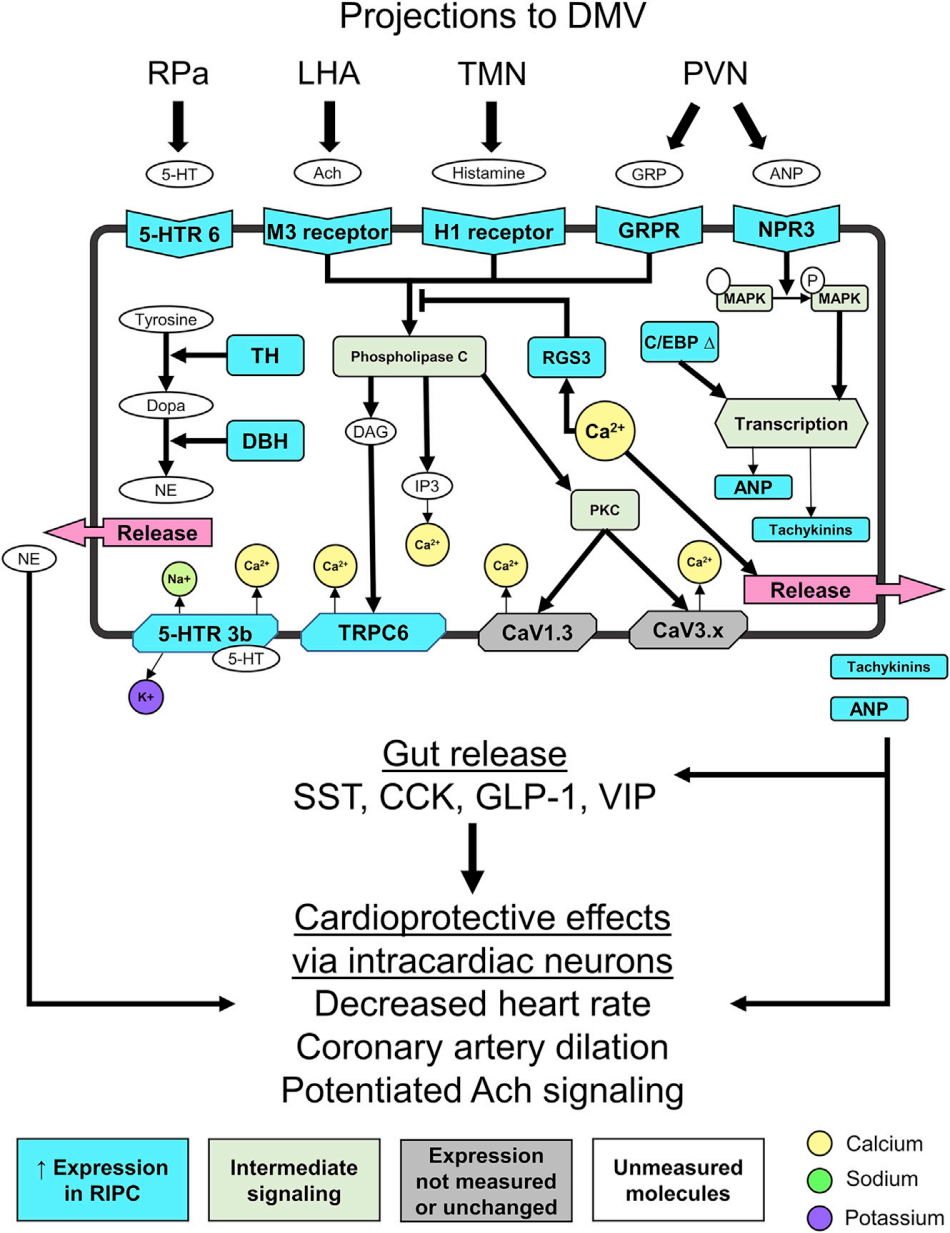
Plausible mechanism meditating RIPC effect in H1-receptor-expressing DMV neurons Schematic of histamine 1 receptor (H1)-expressing neuron highlighting potential mechanism based upon differential gene expression of receptors, intracellular effectors, and neuropeptides. Genes that are uniquely expressed in the H1-expressing neurons of the RIPC cohort compared with non-H1-expressing neurons from the RIPC group and compared with the H1 neurons of the naive and sham cohorts. Brain regions likely acting on the receptors shown are shown along the top above the brackets. All pathway information was derived from literature inferences with several pathway connections representing multiple steps and intermediaries. The combination of genes expressed suggests an increase in calcium influx that Gq signaling that drive the production and release of norepinephrine, atrial natriuretic peptide, and tachykinins from DMV efferent neurons to contribute to cardioprotection derived from remote ischemic preconditioning (RIPC). 5-HTR 6, serotonin receptor 6; M3, muscarinic acetylcholine receptor 3; H1, histamine receptor 1; GRPR, gastrin-releasing peptide receptor; NPR3, naturetic peptide receptor 3; MAPK, mitogen-activated protein kinase; TH, tyrosine hydroxylase; BH, dopamine beta hydroxylase; 5-HTR 3b, serotonin receptor 3b; TRPC6, transient receptor protein 6; DAG, diacylglycerol; IP3, phosphatidylinositol; Dopa, dopamine; NE, norepinephrine; 5-HT, serotonin; Ach, acetylcholine; GRP, gastrin-releasing peptide; ANP, atrial natriuretic peptide; Rpa, raphe pallidus; LHA, lateral hypothalamus; TMN, tuberomammillary nucleus; PVN, paraventricular nucleus; SST, somatostatin; CCK, cholecystokinin; GLP-1, glucagon like peptide 1; VIP, vasoactive intestinal peptide; DBH, dopamine beta hydroxylase; RGS3, regulator of G-protein signaling 3; Cav1.3, voltage-gated calcium channel 1.3; Cav3.x, T-type voltage-gated calcium channels; PKC, protein kinase C; C/EBP Δ, CCAAT/enhancer-binding protein delta.

Although it is more difficult to elucidate a single differentiating factor from the chronic ischemia experiments, the RIPC model has some findings that may be organized in such a way that suggest plausible mechanisms behind the effect. As one means of synthesizing the data from the RIPC neuronal states, there is a significantly larger proportion of neurons expressing Hrh1 in the RIPC group, which begs the question whether this is due to the histamine receptor 1 (H1) expressing phenotype predicted in our data or if there are just more neurons of a similar phenotype. There are several genes that are differentially represented or expressed only in the H1-expressing neurons of the RIPC group. Based on the receptors expressed, these neurons appear to have the chance for increased influence from the PVN, tuberomammillary nucleus (TMN), raphe pallidus (RPa), and lateral hypothalamus (LHA) when compared with all other neurons from RIPC or other groups. A proposed mechanism is described in Figure 6.

## Discussion

In this work, we aim to uncover the molecular substrates that underlie the DMV orchestration of the cardioprotective effects, providing insight as to what signaling cascades might mediate the effect as well as how the DMV itself integrates these signals and modulates target neurons in the gut, heart, and possibly other organ systems to promote cardioprotection. To do this, we first characterized the heterogeneity of DMV neurons at baseline, particularly with regard to their integrative input signal processing capacity leading to combinatorial neuropeptide and neurotransmitter production. Shifts in the distribution of these states occur dynamically in response to surgical stress and RIPC. Unique responses to 3 weeks of chronic cardiac ischemia include changes in gene expression, and subsequent gene regulatory networks show a dramatic shift to a neurosecretory, enteroendocrine-like phenotype in a highly coordinated fashion. The dynamic changes in the DMV neuronal landscape appear to generally occur through selective recruitment of one state from others. The implications of these findings emphasize a need to consider the functional implications of neuronal heterogeneity with the understanding that at times only a subset of neurons from a brain nucleus may exert significant effects on physiology. These subsets may not be appreciated unless experimental work is done with sufficient granularity and time-dependent sampling.

The DMV is one of the few brain regions uniquely positioned to coordinate homeostatic organ function within the body. The anatomy of afferent connections and distribution of vagal efferents has been well known, and this work provides the context needed to demonstrate the heterogeneity of DMV processing functionality. The distinct neuronal states that shift in adaptive or maladaptive directions provide a conceptual framework for thinking about how the DMV attempts to maintain the body's homeostasis. Conditions like RIPC or other ischemic preconditioning may function as hormetic stresses that encourage the state shifts in the DMV necessary to manage the systemic effects of cardiac ischemia, whereas the shifts observed after several weeks of persistent cardiac ischemia may demonstrate a compensatory response to augment the stress response.

In RIPC, there was a significant upregulation of the generally sparsely expressed H1 receptor gene (Hrh1), both in expression level as well as in number samples with expression above the limit of detection (Figures 6, S10, and S11). There is one known source of histaminergic projections to the DMV, the TMN of the hypothalamus (Poole et al., 2008; Connelly et al., 2009; Green 1978). Activation of the H1 receptor in the dorsal vagal complex has been shown to mimic the effects of satiety, reducing hyperphagia in rodent models of obesity (Deng et al., 2010). This is in line with its general ability to mediate depolarization in the low number of neurons in the DMV that express it (Poole et al., 2008). Such satiety signaling may be co-opted to mediate cardioprotective effects (Mastitskaya et al., 2016; Basalay et al., 2016; Gorky and Schwaber 2019). Although there is evidence that the TMN is responsive to cardiovascular perturbations and can indirectly modulate cardiovascular function (Yamanaka et al., 2017; Bealer 1999), this is the first indication we are aware of for its role in mediating cardioprotection in any sense. There are several genes that are differentially represented or expressed only in the H1-expressing neurons of the RIPC group. Of particular interest are significant decreases in expression in Sst and Cck. Somatostatin, delivered locally into the gut, decreases the release of somatostatin (Ipp et al., 1979), cholecystokinin (Herzig et al., 1994), glucagon-like peptide 1 (Chisholm and Greenberg 2002), and vasoactive intestinal peptide (Singh et al., 1988), thus the decrease in Sst expression effectively indicates an increased chance for release of these peptides. Coupled with this decrease is a markedly increased expression of Tac1 and Nppa. Neurokinins and atrial natriuretic peptide in the gut induce the release of the same four peptides mentioned above along with several others (Gower et al., 2003; Verspohl and Ammon 1989; Pawlik et al., 1992; Holzer 2001; Tien et al., 1991; González Bosc et al., 2000). Apart from action at the gut, both tachykinins and atrial natriuretic peptide (ANP) have well-described effects on the heart including decreased heart rate (Clemo et al., 1996; Tompkins et al., 1999), coronary artery vasodilation (Hoover et al., 2000), and potentiation of acetylcholine signaling (Chang et al., 2000; Zhang et al., 2005). Furthermore, the combination of norepinephrine and ANP likely works on post ganglionic neurons in the heart to mediate sympathoinhibition that was once thought to require input from at least two different neurons (Herring and Paterson 2009; Perrin and Gollob 2012; Floras 1995), but we now show that it may plausibly come from the same vagal preganglionic neurons. There are several receptor and ion channels whose expression is increased and whose coordinated effects would lead to increased excitability and release of peptide transmitters (Kim et al., 2017; Gardam and Magoski 2009; Hille 1994; Blackburn 2009; Zamponi and Snutch 1998). There is also a shift from high expression of regulator of G-protein signaling 2 (Rgs2) in other neurons to higher regulator of G-protein signaling 3 (Rgs3) expression. Rgs2 is an inhibitor of G_q_ and enhancer of G_i/o_ signaling, whereas Rgs3 is responsive to increased levels of Ca^2+^ as a feedback regulator of G_q_ signaling, not a tonic inhibitor like Rgs2. Based on the receptors expressed, these neurons have increased influence from the PVN, TMN, RPa, and LHA when compared with all other neurons from RIPC or other groups. These results point to a neural circuit for centrally mediated cardioprotection that may be exploited to stimulate an effect similar to RIPC.

Although a distinct subset of neurons mediates the effects of RIPC over the course of hours, there are significant shifts in the population landscape of neuronal states after 3 weeks of chronic cardiac ischemia. These shifts occur from the recruitment of neurons to a putative neurosecretory potential, particularly on the right side of the DMV more than the left, as shown in State-W, State-X, and State-Y. This programmatic response appears mediated, at least in part, by paired box 4a (Pax4a), a transcription factor that is strongly regulated by the transcriptional repressor REST, which drives stem cells into a neurosecretory phenotype (Bruce et al., 2006; Kemp et al., 2003). Disinhibition of gene expression through the downregulation of REST with concomitant upregulation of Pax4a is restricted largely to neurons, but it also occurs in developing/regenerating pancreatic islet cells, in particular β and δ cells (Biason-Lauber et al., 2005). The coordination of increased expression of the genes in GC-5 may be accomplished in part through effects of Pax4a as evidenced by its unique network connectivity in that condition (Figure S18). Coupled with the coordinated upregulation of voltage-gated calcium channel subunits Cacna1b, Cacna1d, as well as cholecystokinin (Cck), and GABA receptor subunits Gabra1 and Gabra2, the picture is painted less of a neuron and more of an enteroendocrine cell, like pancreatic β/δ cells or colonic L/I cells (Gameiro et al., 2005; Rogers et al., 2011; Schonhoff et al., 2004). Canonical satiety peptides (CCK, GLP-1, SST, etc.) have a protective effect on the peripheral organs in the setting of ischemic damage at the heart (Sartor and Verberne 2010; Mukharji et al., 2013; Grossini et al., 2013; Goetze et al., 2016; Dong et al., 2017; Mobley et al., 2006; Gorky and Schwaber 2019). That the DMV may deliver peptides directly to postganglionic neurons in the periphery adds dimension to the concept of vagal tone as a mediator of health and disease.

The National Institutes of Health (NIH) programmatic initiative aimed at the development of means to Stimulate Peripheral Activity to Relieve Conditions (SPARC) was motivated by therapeutics derived from electrical stimulation of the vagus nerve in multiple contexts. We highlight here the complexity and plasticity of the vagal efferent neurons from the DMV. We further show that acute DMV responses only involve a subset of neurons mediated by distinct effector neurotransmitters and neuropeptides. This suggests that there are molecular means by which stimulation of autonomic peripheral activity may also involve specific neuropeptides or other effectors to mediate more specific effects. As we continue to personalize medicine, we will continue to specify therapeutics. This work provides a framework for investigations to uncover more specific means by which the autonomic nervous system mediates physiological homeostasis and compensates for disease states.

### Limitations of the study

• The LCM single neuron data on gene expression provided information on neuronal states based on an extensive list of assays selected for their relevance to the signal processing circuits. Additional transcriptomics data can provide information in correlated pathways across other relevant processes, e.g., metabolism and inflammation.
• Our results show preliminary findings on bilateral differences in gene expression and neuronal states in the DMV. Increasing the sampling numbers of neurons may lead to identification of additional right versus left DMV-specific neuronal states.
• Potential sex-specific differences in DMV gene expression and response to physiological perturbations were not considered in the present study.

### Resource availability

#### Lead contact

Further information and requests for resources should be directed to and will be fulfilled by the Lead Contact, James S. Schwaber: james.schwaber@jefferson.edu.

#### Material availability

This study did not generate new unique reagents.

#### Data and code availability

The authors declare that all the data supporting the findings of this study are available within the article and its supplemental information files or from the corresponding author upon reasonable request. All the raw and processed HT-qPCR data as well as Nanostring microRNA profiling data are available online in the GEO database under SuperSeries GSE155053, which contains links to the individual datasets (HT-qPCR data: GSE155047 and GSE155049; Nanostring data: GSE154990).

## Methods

All methods can be found in the accompanying Transparent Methods supplemental file.

## Supplemental information

Document S1. Transparent methods, figures S1–S21, and tables S2–S4

Table S1. Assay selection rationale, related to figure 1

## Footnotes

Supplemental information can be found online at https://doi.org/10.1016/j.isci.2021.102143.
