# Supplementary material for: Input-output signal processing plasticity of vagal motor neurons in response to cardiac ischemic injury
Source: iScience. 2021 Feb 4;24(3):102143. doi: 10.1016/j.isci.2021.102143 (PMC7898179; doi:10.1016/j.isci.2021.102143)
Supplement: Document S1. Transparent methods, figures S1–S21, and tables S2–S4 [file mmc1.pdf]

iScience, Volume *24*

Supplemental Information

Input-output signal processing plasticity of vagal motor neurons in response to cardiac ischemic injury

Jonathan Gorky, Alison Moss, Marina Balycheva, Rajanikanth Vadigepalli, and James S. Schwaber

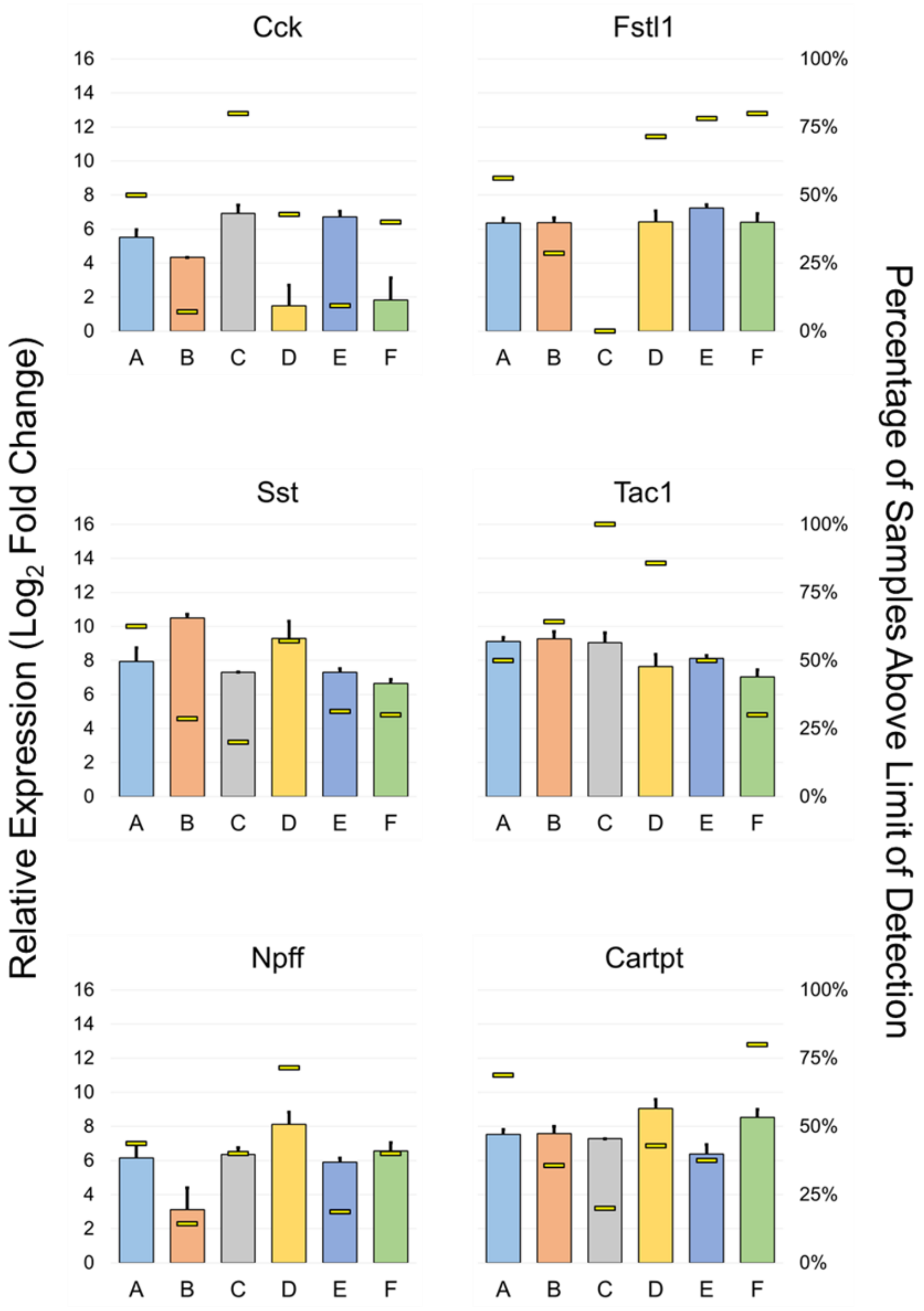

Figure S1: Expression of neuropeptides across neuronal states in the DMV, related to Figures 1,2. Sample cluster expression of genes that may indicate multiple peptidergic states. As with other figures, the left axis corresponds with the colored box with error bar (SEM) that represents gene expression (log2 relative gene expression with zero as limit of detection). The right axis represents the percentage of samples within each cluster that have an expression value above the limit of detection and is shown by the narrow bar in each group.

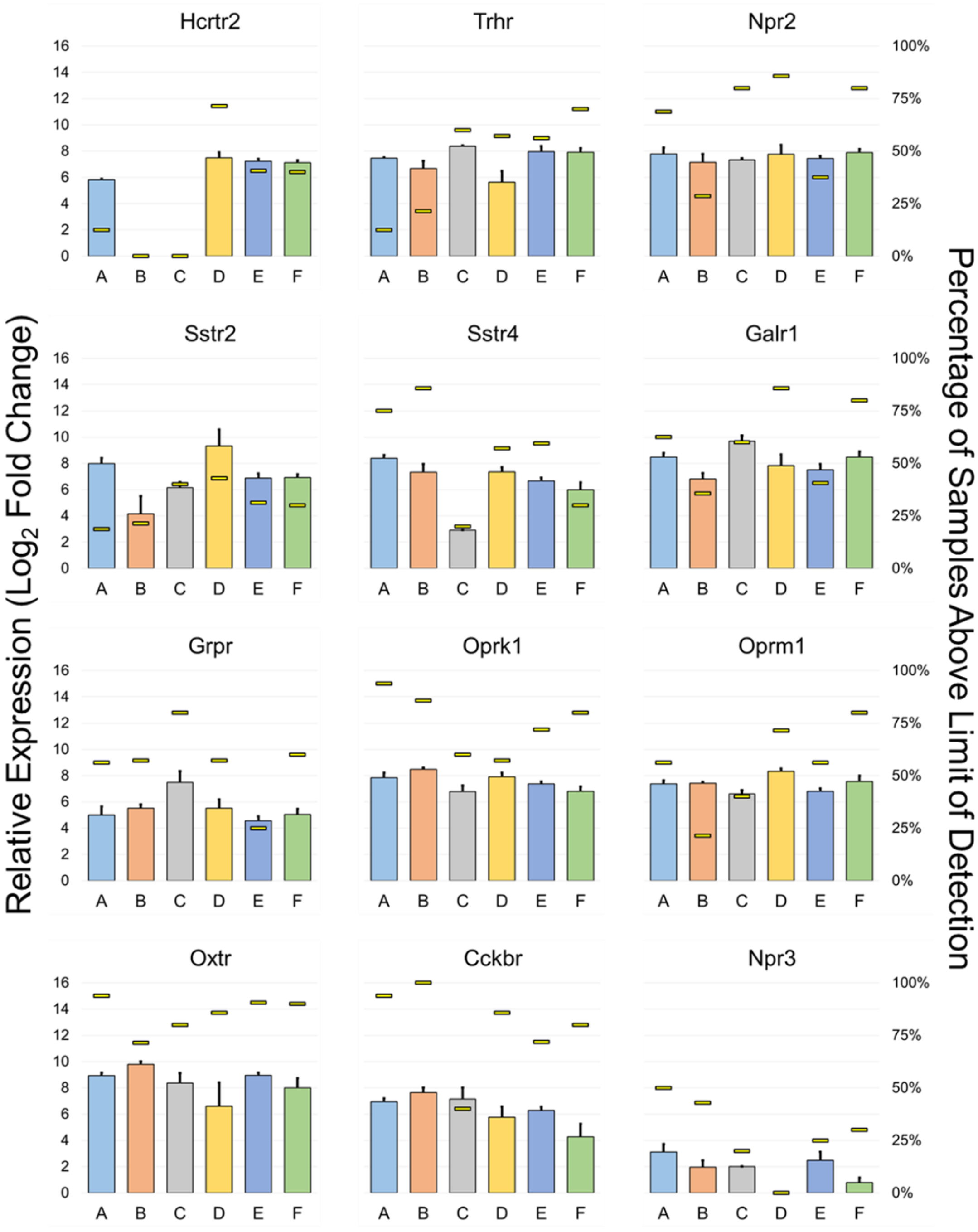

Figure S2: Expression of neuropeptide receptors across neuronal states in the DMV, related to Figures 1,2. Sample cluster expression of genes that code for peptide receptors may help determine which neurons receive peptidergic inputs from specific regions of the brain. As with other Figures, the left axis corresponds with the colored box with error bar (SEM) that represents gene expression (log2 relative gene expression with zero as limit of detection). The right axis represents the percentage of samples within each cluster that have an expression value above the limit of detection and is shown by the narrow bar in each group.

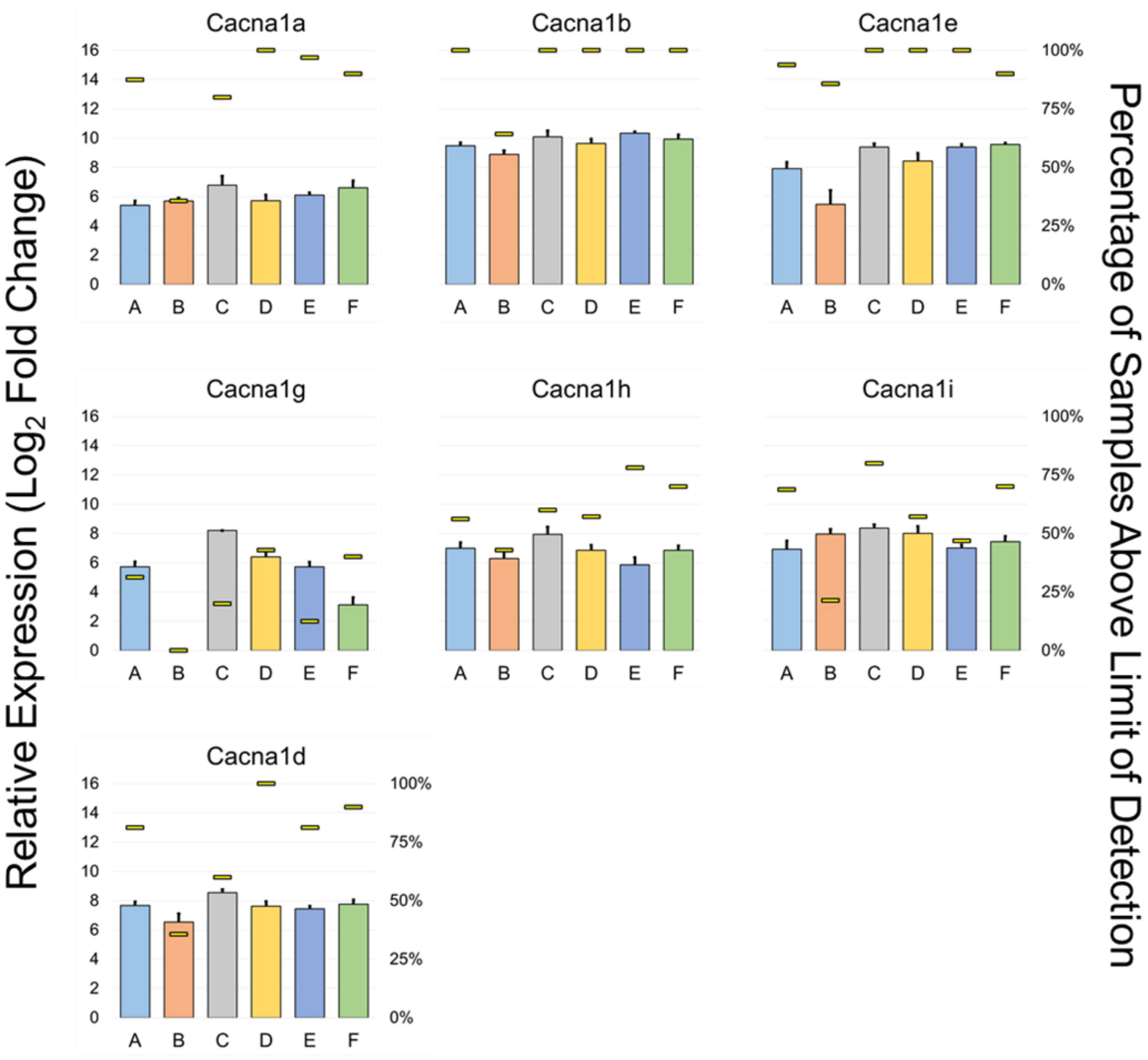

Figure S3: Expression of calcium channels across neuronal states in the DMV, related to Figures 1,2. Sample cluster expression of genes that code for calcium channel subunits. Due to the consideration of calcium fluxes in DMV neurons driving apoptosis as described by Surmeier along with the generation of pacemaker-like activity (Goldberg et al., 2012), the distinct expression of calcium channel mix is of interest here. While there is general consistency in the high-voltage gated calcium channel subunits (Cacna1a, Cacna1b, and Cacna1e), there is a great deal of heterogeneity among the low-voltage gated channels (Cacna1g, Cacna1h, Cacna1i). This may suggest differences in burst firing or peacemaking activity. As with other figures, the left axis corresponds with the colored box with error bar (SEM) that represents gene expression (log2 relative gene expression with zero as limit of detection). The right axis represents the percentage of samples within each cluster that have an expression value above the limit of detection and is shown by the narrow bar in each group.

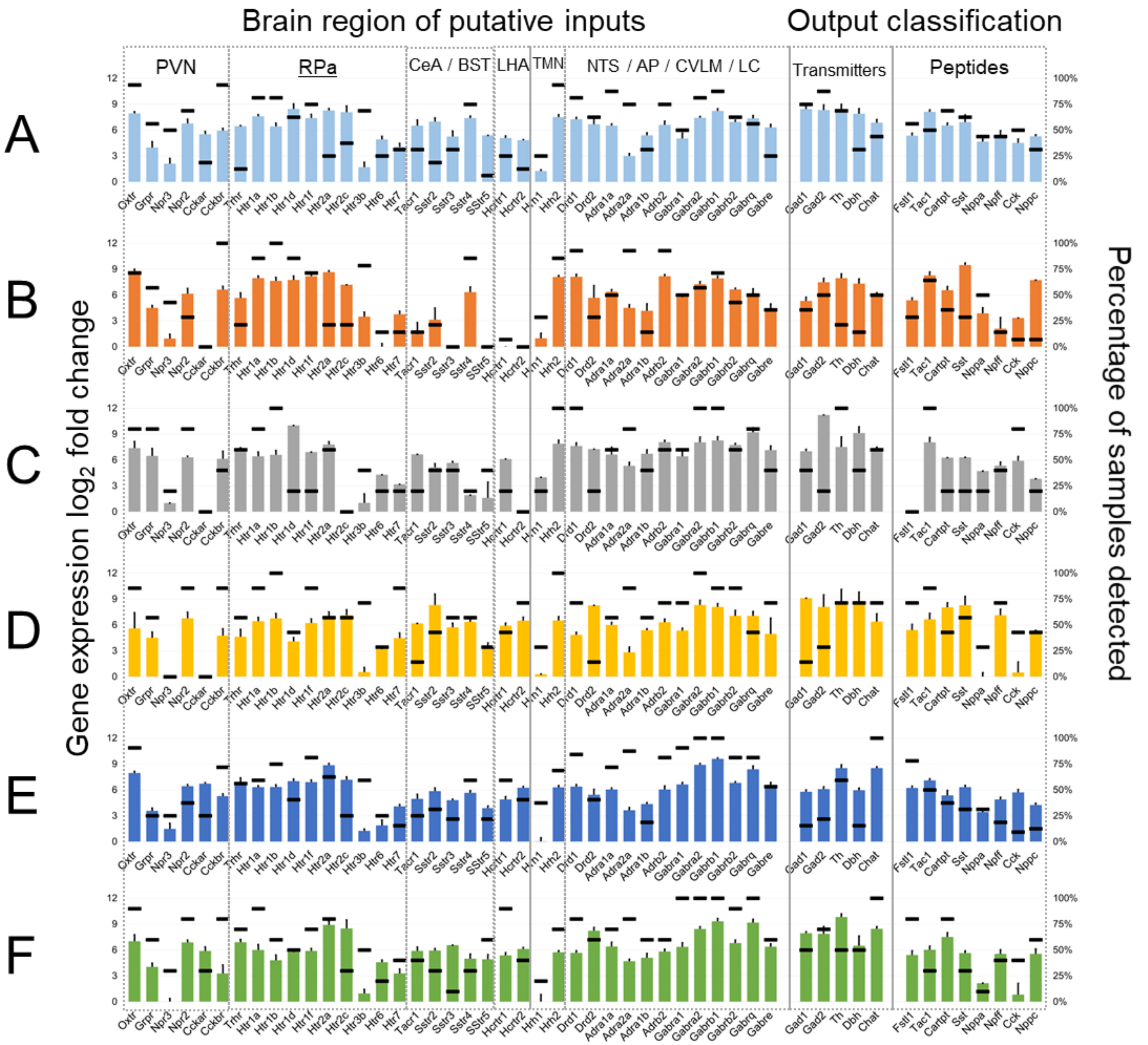

Figure S4: Input output signal processing map of DMV neuronal states, related to Figure 2. Datasets showing gene expression and sample proportion data for each state. These particular data were used to construct the input/output maps as shown in Figure 2.

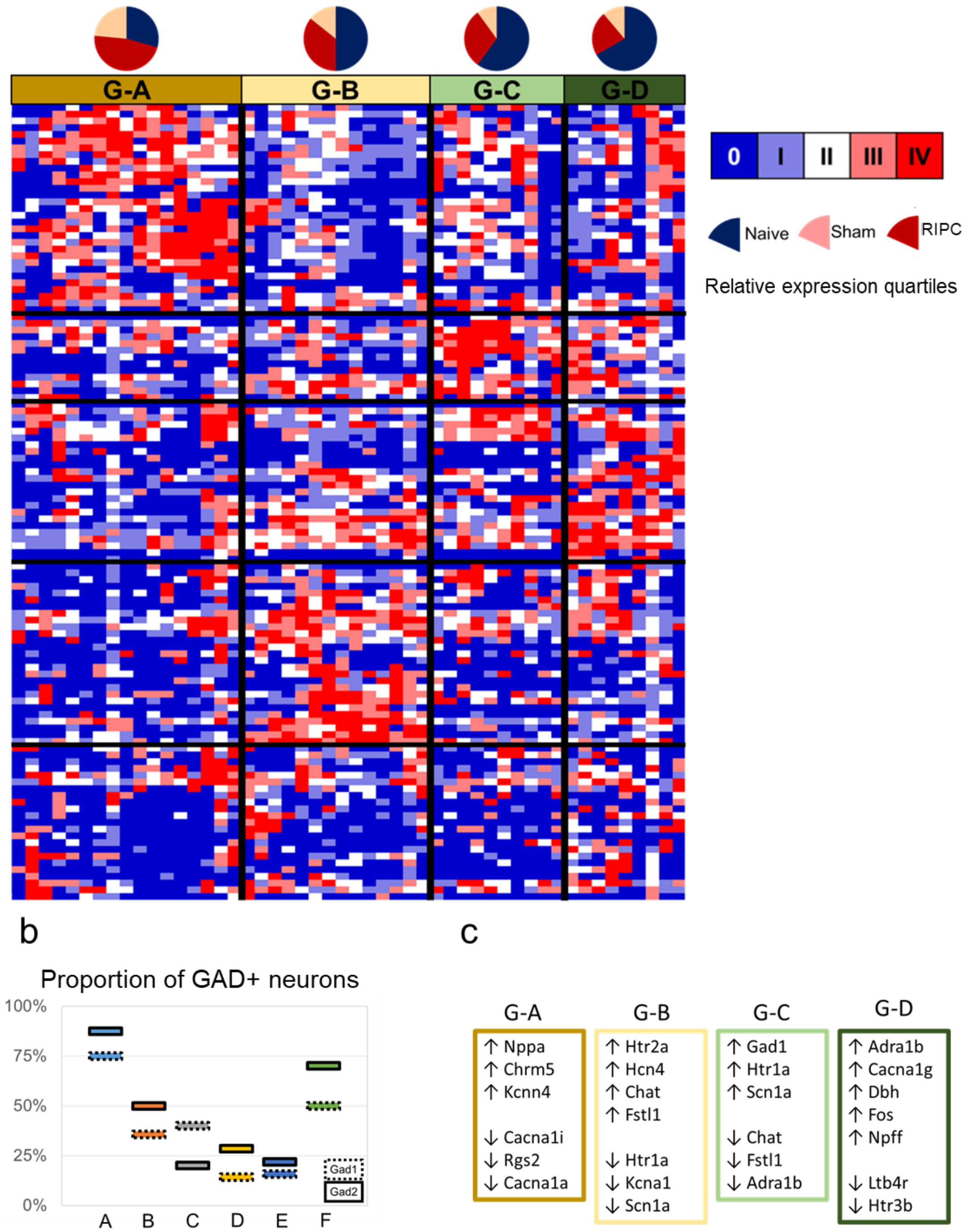

Figure S5: Clustering of Gad+ single neurons, related to Figure 3. (A) Heatmap of Gad+ single cells with distributions from experimental treatment groups. Pooled samples were omitted from this aspect of the analysis in order that anything found to be co-expressed with Gad1 or Gad2 could be attributed to a single cell as real co-expression. Four clusters emerge from this analysis that were determined without regard for the clusters found in Figure 3A in order that the most potentially meaningful and strong correlations could be identified. **(B)** Proportion of samples from each of the original neuronal states in Figure 3 that are positive for *Gad1* or *Gad2*. **(C)** Selected genes that define the unique phenotypes of GABAergic neurons of the DMV.

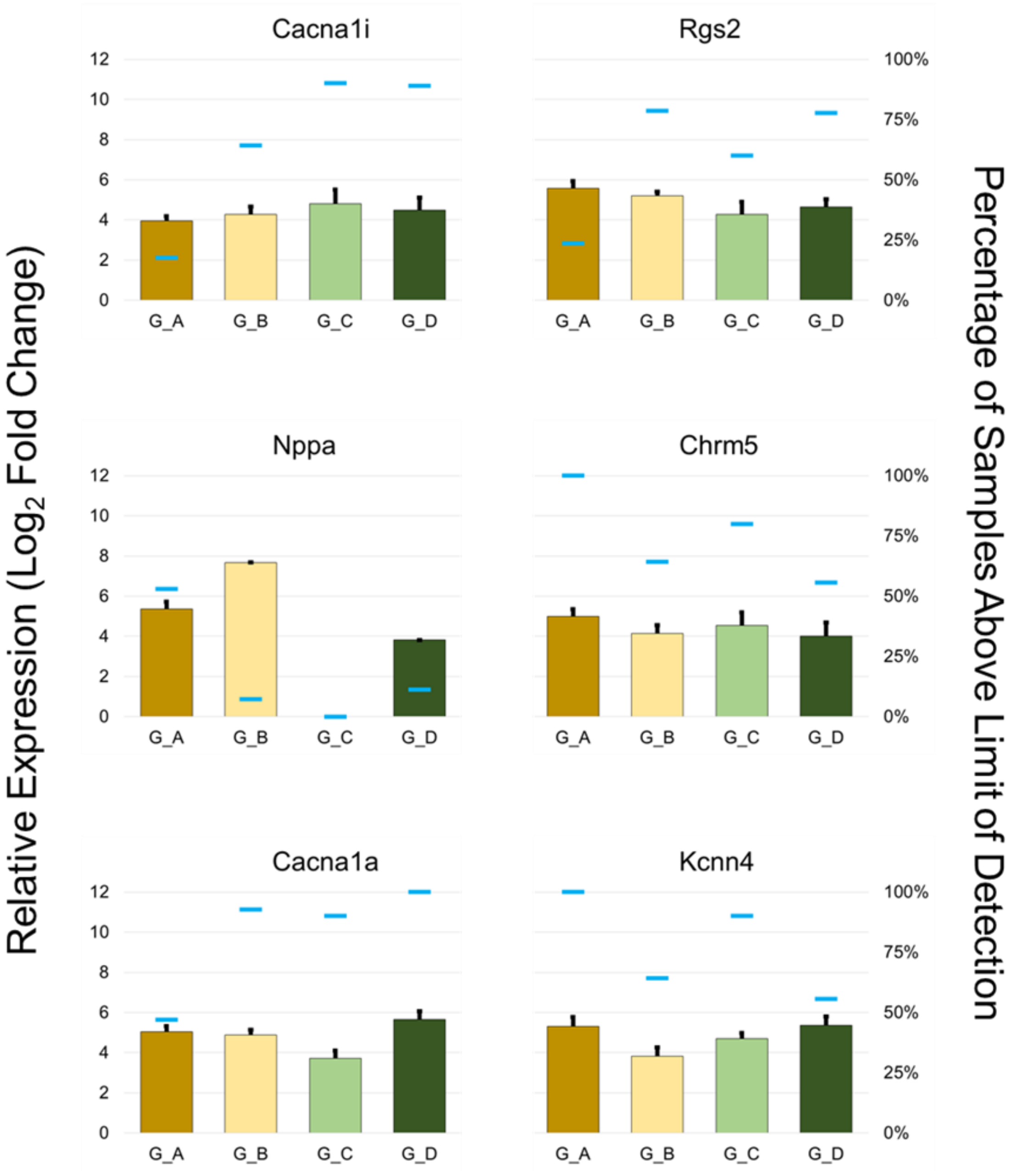

Figure S6: Genes with distinct expression patterns in cluster G_A, related to Figure 3, Figure S5. Binomial testing with a cutoff $p<0.001$ comparing G_A with at least two other groups was used to determine inclusion in this set. As with other Figures, the left axis corresponds with the colored box with error bar (SEM) that represents gene expression (log2 relative gene expression with zero as limit of detection). The right axis represents the percentage of samples within each cluster that have an expression value above the limit of detection and is shown by the narrow bar in each group.

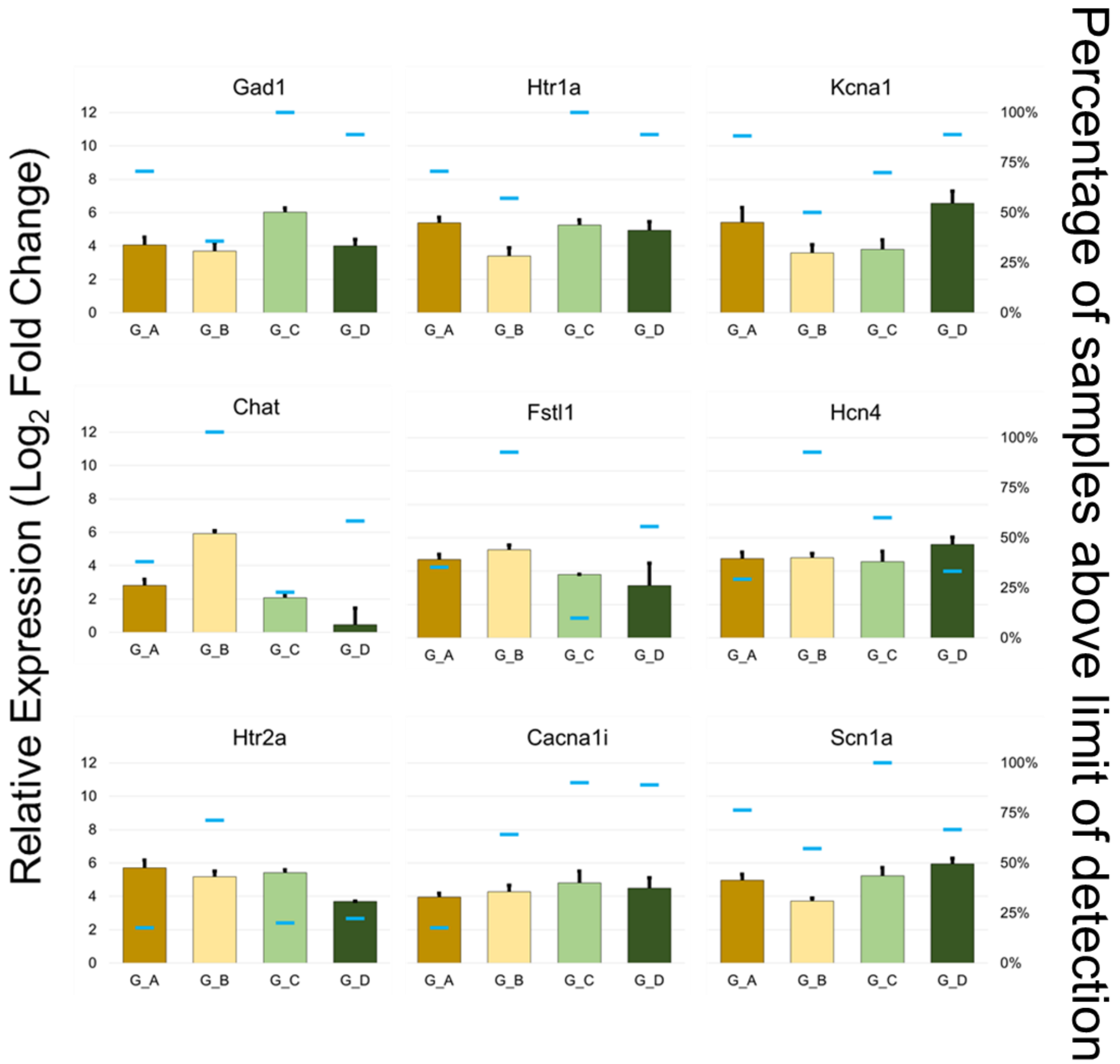

Figure S7: Genes with distinct expression patterns in cluster G_B and/or G_C, related to Figure 3, Figure S5. These clusters are included together due to the often opposite extremes in gene expression. Many of the genes collected here not only have significant differences in number of neurons with expression above the limit of detection, but also in the levels at which the gene is expressed in such neurons (ANOVA with Tukey post-hoc $p<0.05$, Exceptions: Hcn4, Htr2a, and Cacna1i). Binomial testing with a cutoff $p<0.001$ comparing G_B or G_C with at least two other groups was also used to determine inclusion in this set. As with other Figures, the left axis corresponds with the colored box with error bar (SEM) that represents gene expression (log2 relative gene expression with zero as limit of detection). The right axis represents the percentage of samples within each cluster that have an expression value above the limit of detection and is shown by the narrow bar in each group.

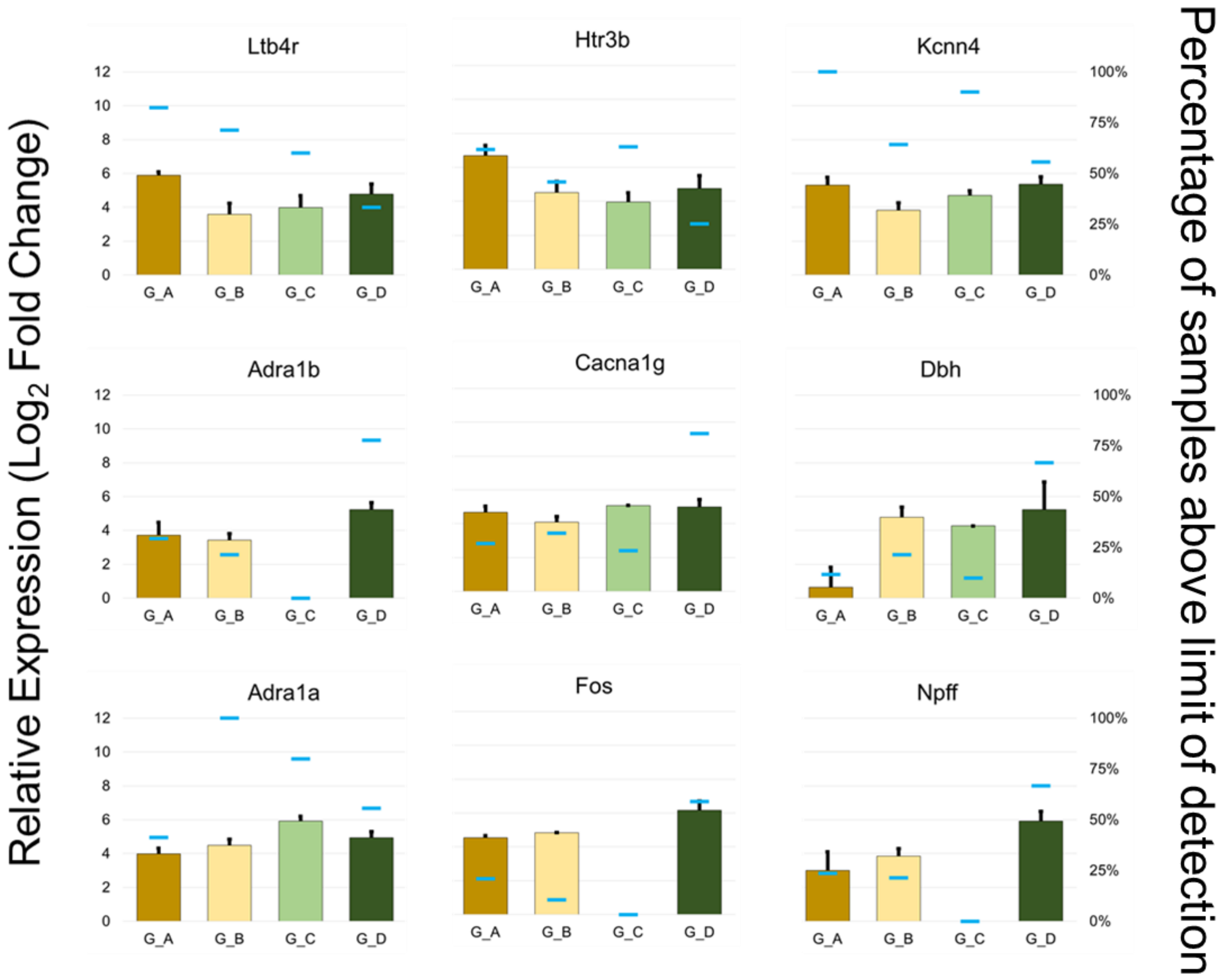

Figure S8: Genes with distinct expression patterns in cluster G_D, related to Figure 3, Figure S5. Binomial testing with a cutoff p<0.001 comparing G_D with at least two other groups was used to determine inclusion in this set. As with other Figures, the left axis corresponds with the colored box with error bar (SEM) that represents gene expression (log2 relative gene expression with zero as limit of detection). The right axis represents the percentage of samples within each cluster that have an expression value above the limit of detection and is shown by the narrow bar in each group.

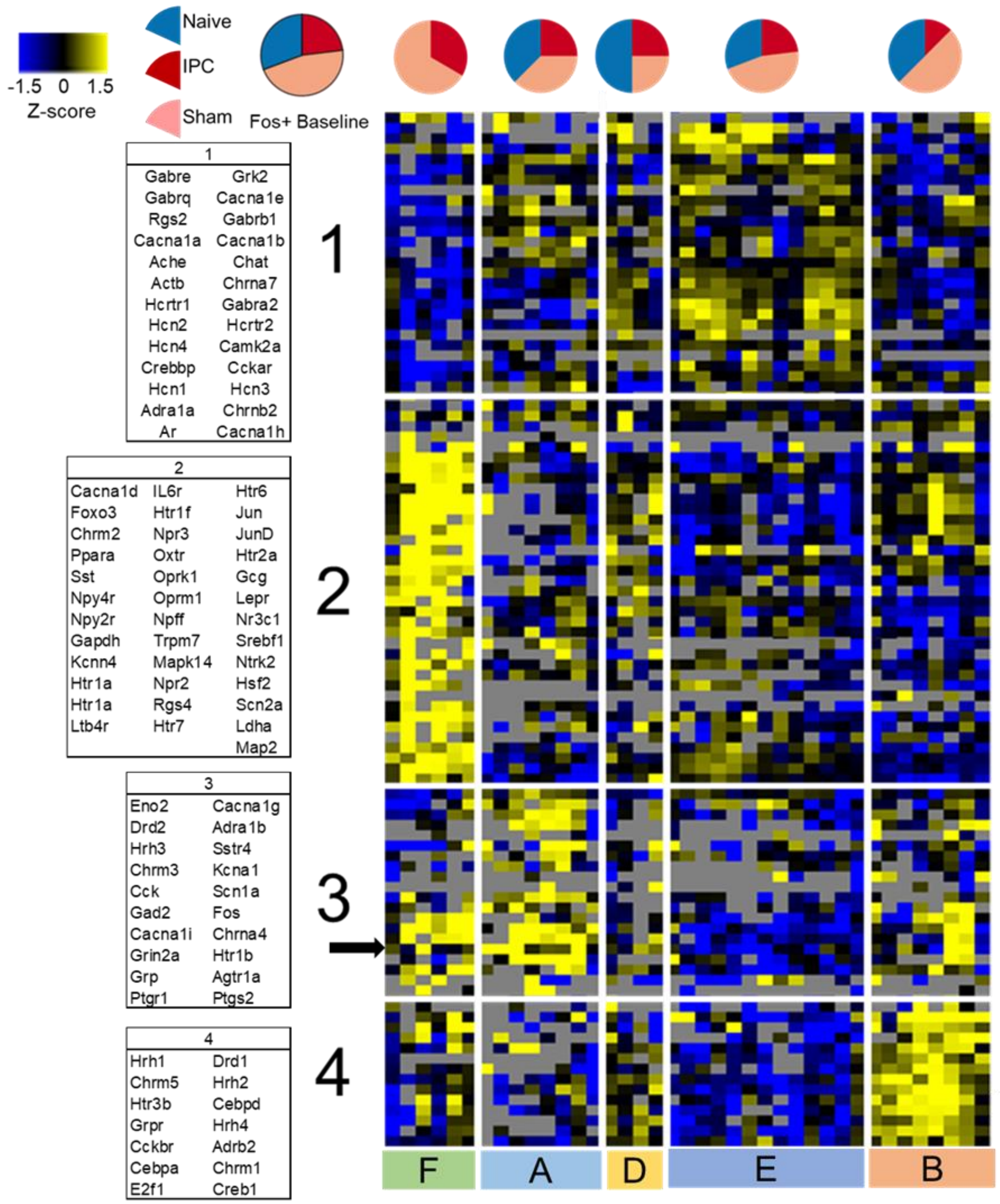

Figure S9: Clustering of Fos+ single neurons, related to Figure 3. Heatmap showing only Fos+ samples suggests characterization of a surgical effect, but not a specific RIPC effect. Heatmap is colorized by Z-score (Z±1.5) within Fos+ samples only. Genes that comprise each cluster are given to the left of the heatmap, going down the columns then from left to right ascertain gene order on the heatmap. The row corresponding to Fos is shown by the black arrow in gene cluster 3. While the sample clusters of Figure 1B were not completely recapitulated here, there are similar patterns that emerged from the *de novo* hierarchical clustering algorithm performed on this subset.

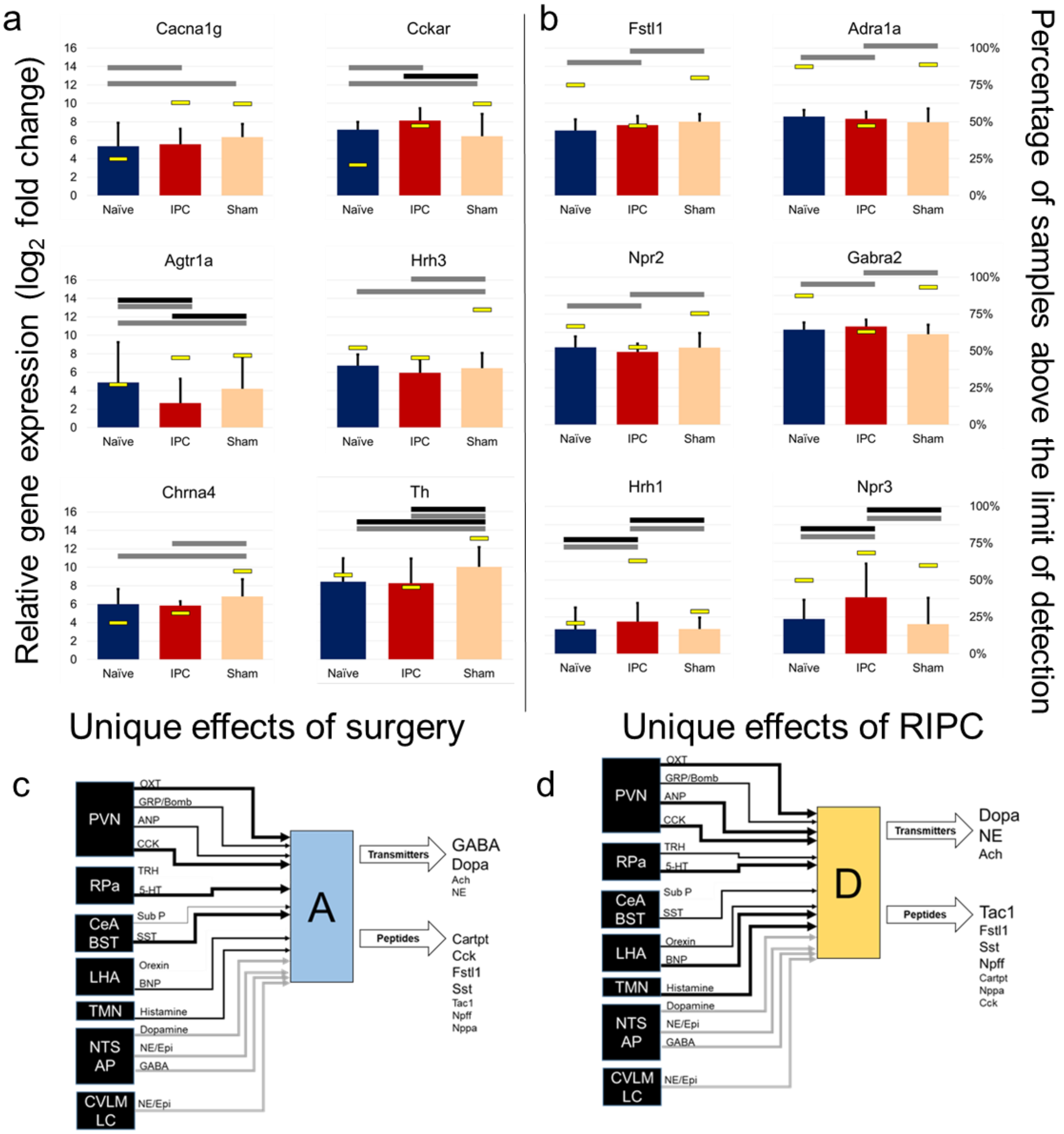

Figure S10: Effects of Sham and RIPC, related to Figure 3. (A) In a demonstration of the effects of the stress of surgery or anesthetic (Sham), several genes were differentially expressed in both the RIPC and sham cohorts as compared with the naïve**.** In the case of Hrh3 and Chrna4, there appears to be a sham only effect, suggesting that the surgical treatment effects are mitigated by the addition of transient peripheral ischemia **(B)** Genes altered uniquely in RIPC versus either sham or naïve cohorts. Data shown here is for pooled samples only in order to generate the most accurate direct comparisons. For each of these genes, the single cell data mirrored the pooled sample data. The cut-off criteria used was a combination of binomial testing (gray bar, $p<0.001$) and/or pairwise T-test (black bar, $p<0.05$) to determine differences in percent of samples above limit of detection and expression values of expressing samples. Bars shown are mean±SEM. **(C)** Neuronal state A with disproportionate association with the Sham condition as shown in state A_2. **(D)** Neuronal state D contains several uniquely upregulated genes in RIPC.

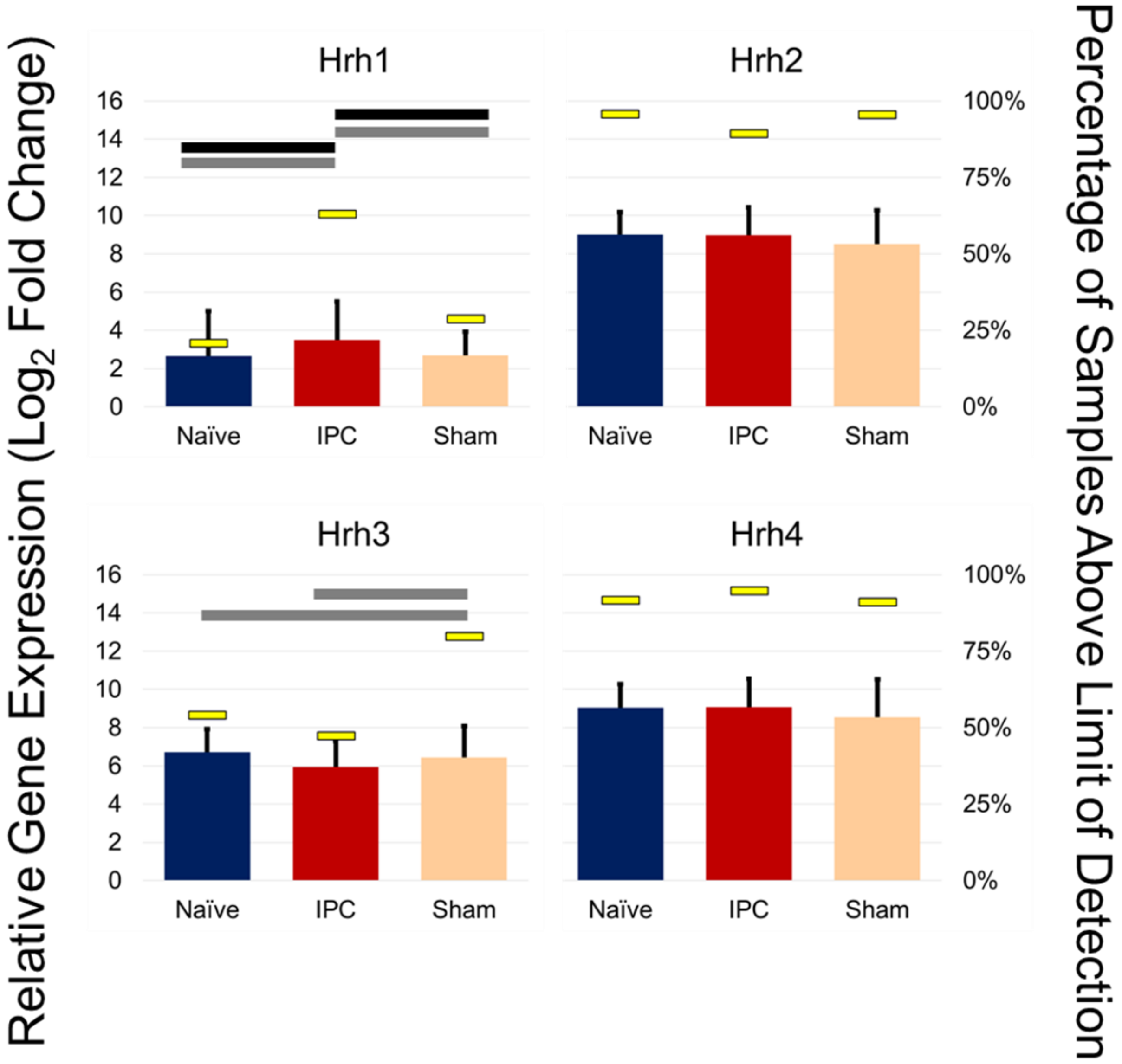

Figure S11: Histamine receptor subtype expression in response to RIPC and sham surgery, related to Figure 3. While the surgery itself leads to an increase in Hrh3 expression, this is suppressed in the RIPC to remain at naïve levels. In place of an increase in Hrh3 is an increase in Hrh1. The cut-off criteria used was a combination of binomial testing (gray bar, $p<0.001$) and/or pairwise T-test (black bar, $p<0.05$) to determine differences in percent of samples above limit of detection and expression values of expressing samples. As with other Figures, the left axis corresponds with the colored box with error bar (SEM) that represents gene expression (log2 relative gene expression with zero as limit of detection). The right axis represents the percentage of samples within each cluster that have an expression value above the limit of detection and is shown by the narrow bar in each group.

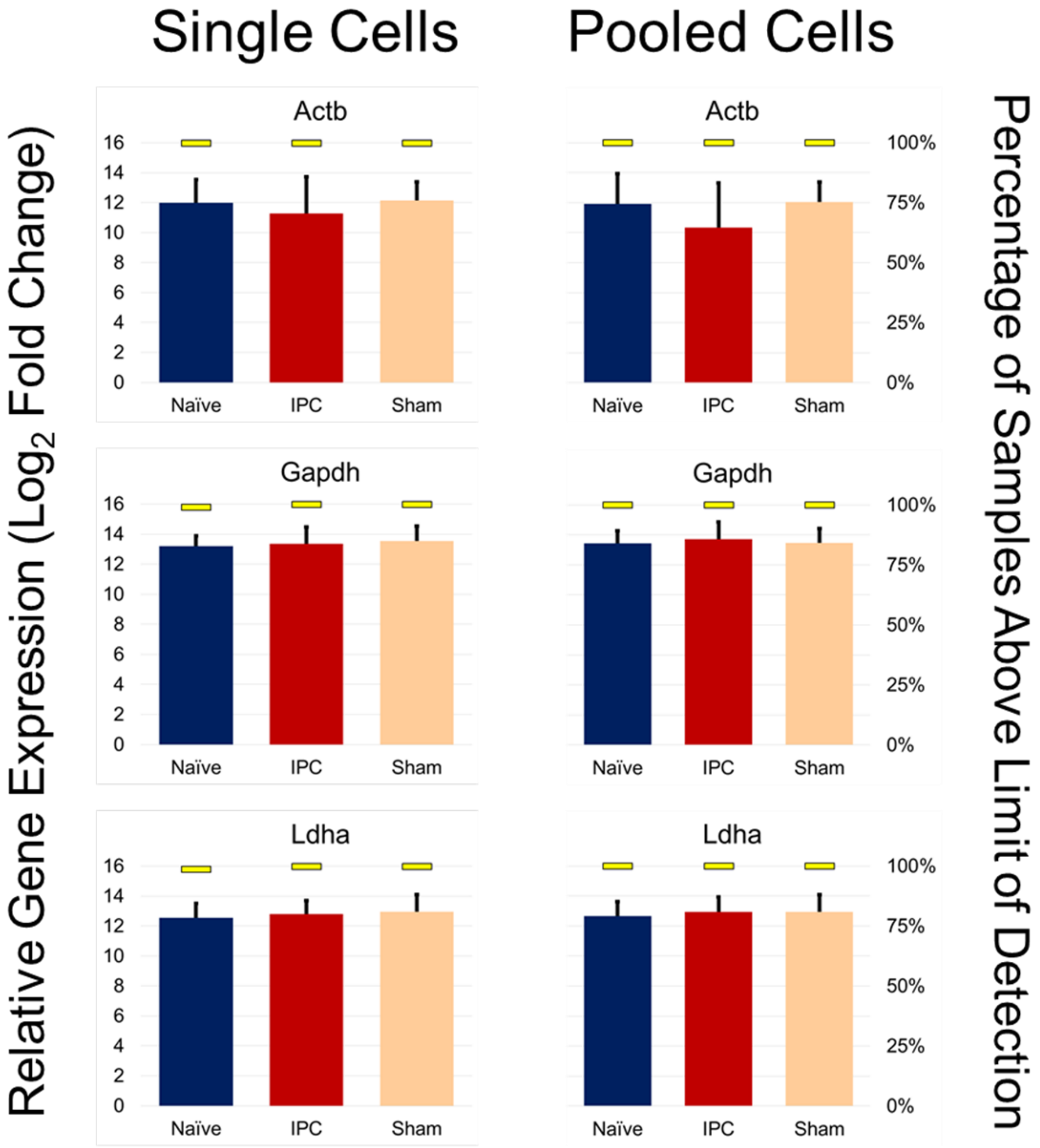

Figure S12: Reference gene expression in response to RIPC and sham surgery, related to Figure 3. Reference genes show no differences between treatment groups nor between single cells and pooled single cell samples. Given the differing number of single cell and pooled samples within each treatment group at baseline, it is important that the technical differences in assaying single cells versus pools not persist after sample median centering normalization. As with other Figures, the left axis corresponds with the colored box with error bar (SEM) that represents gene expression (log2 relative gene expression with zero as limit of detection). The right axis represents the percentage of samples within each cluster that have an expression value above the limit of detection and is shown by the narrow bar in each group.

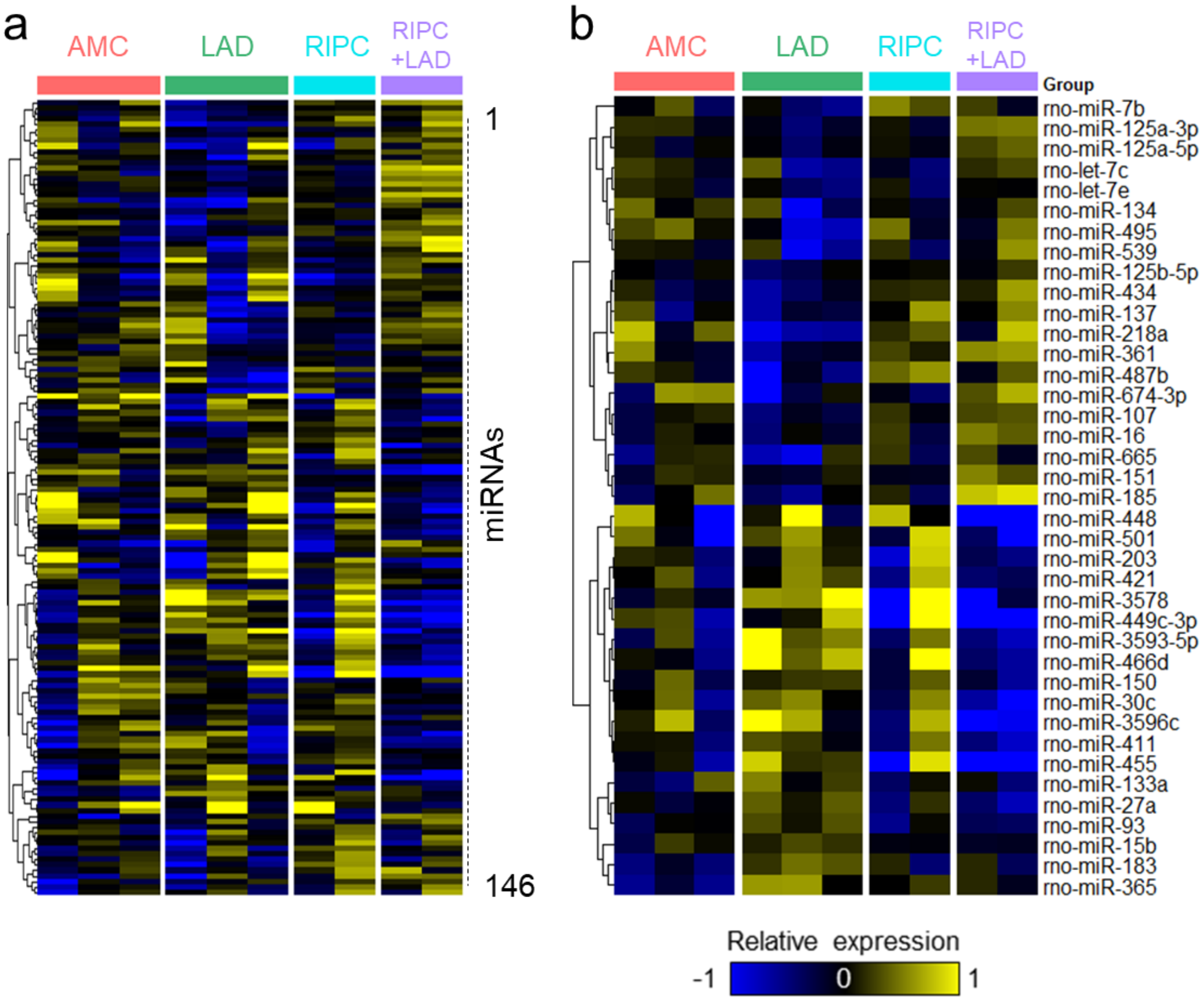

Figure S13: Expression profiles derived from Nanostring assays of microRNAs in whole DMV punches in response to LAD ligation with and without RIPC, related to Figure 4. (A) A total of 146 microRNAs were detected. **(B)** Template analysis identified a subset of microRNAs that showed dysregulation in LAD and normalization by RIPC. Samples are shown along the horizontal axis of the heatmap and microRNAs on the vertical axis.

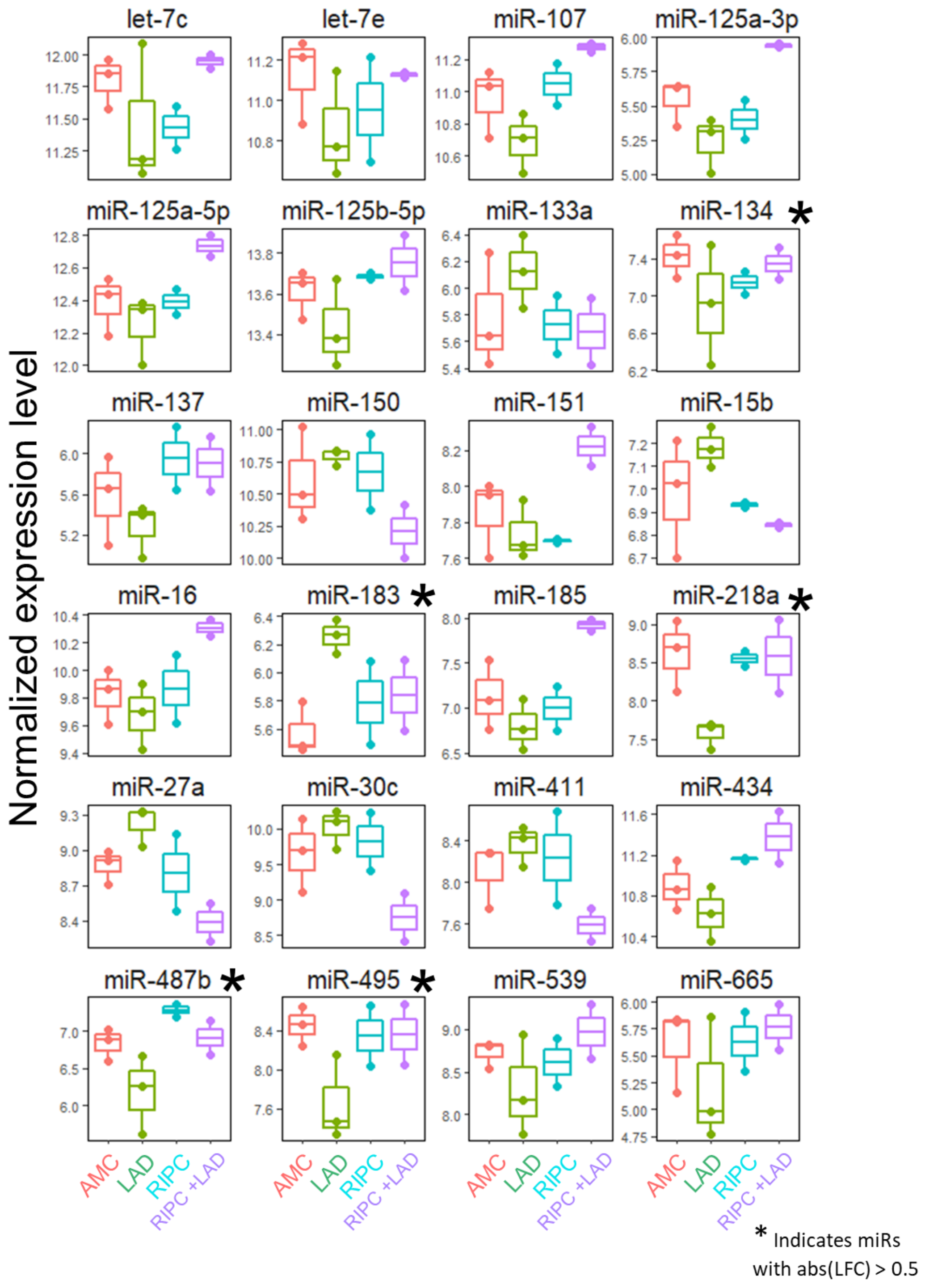

Figure S14: Expression profiles derived from Nanostring assays of microRNAs in whole DMV punches in response to LAD ligation with and without RIPC, related to Figure 4. Of the 39 miRNAs from template matching (Figure S13B), 24 miRNAs have average expression above 5.5. Box plots provide distribution of expression values for each experimental group, *Indicates miRs with abs(LFC) > 0.5.

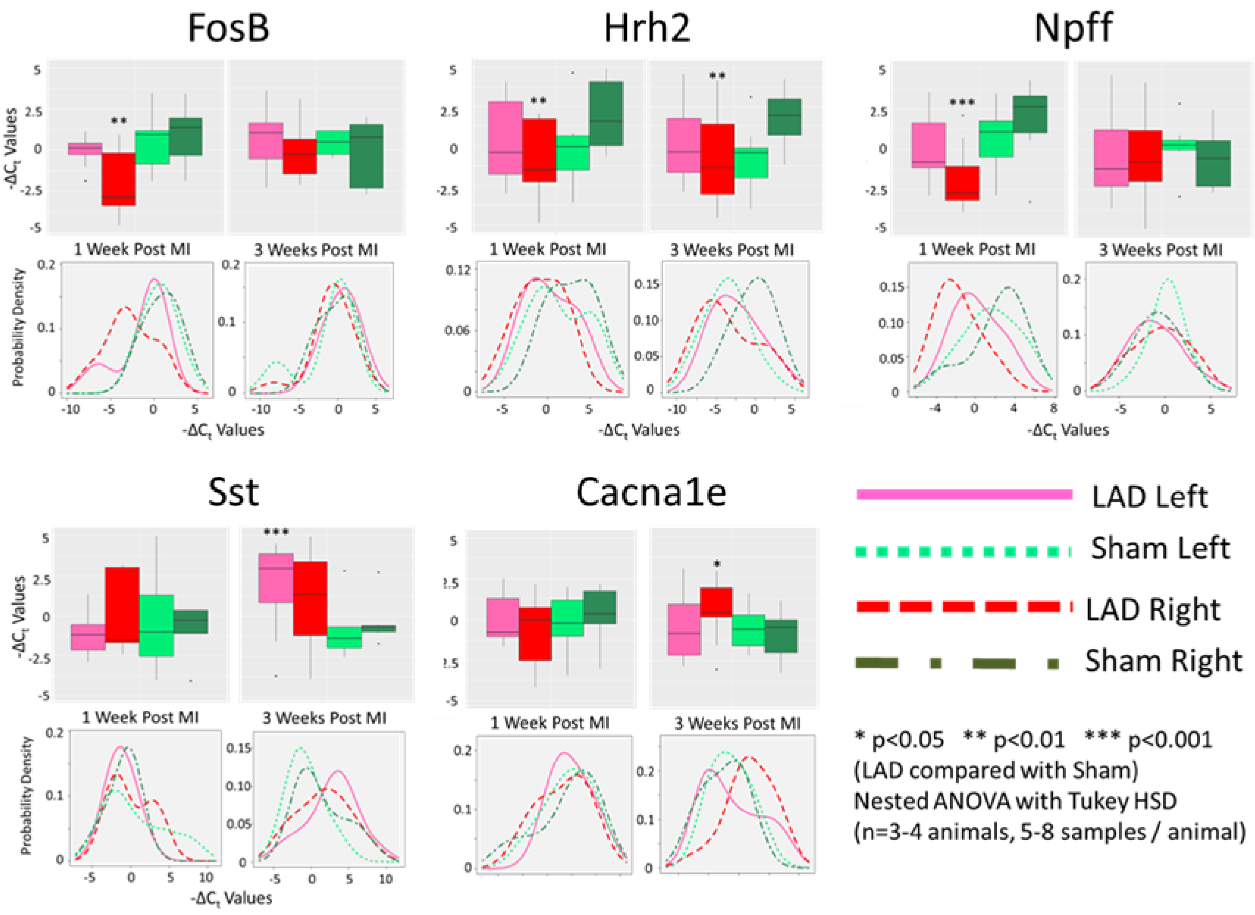

Figure S15: Genes with statistically different expression between experimental groups showed bilateral asymmetry, related to Figure 5. Of all the genes examined, only five showed statistically distinct expression levels between sham and LAD for either time point when accounting for animal level effects using a mixed linear model nested ANOVA (lme4 package in R). For each gene, boxplots of gene expression values are shown broken down by quartiles. Asterisks (*) indicate statistically significant differences between the sham and LAD surgery groups within a given time point on either the left or right side (as indicated by box/line color). Also shown for each gene is a density plot for the expression values for both left/right and sham/LAD conditions within a time point.

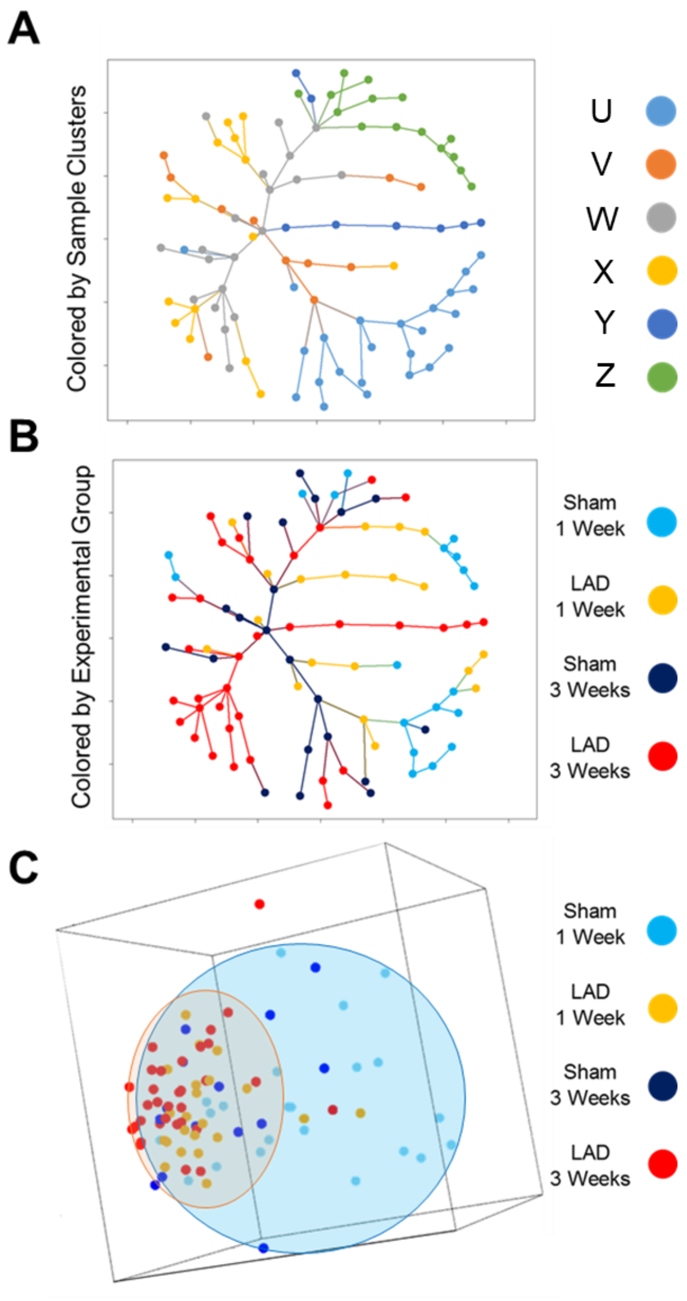

Figure S16: Dimensionality reduction though minimum spanning tree and principal component analysis demonstrates diminished expression space for LAD-3 samples toward the SC-C, SC-D, and SC-E expression patterns, related to Figure 5. **(A)** Minimum spanning tree colored by sample clusters shows that SC-B and SC-C are more transitional phenotypes with SC-A, SC-D, SC-E, and SC-F being more terminal. **(B)** The same minimum spanning tree as in (**A)**, but colored for experimental groups, shows the unique terminal branches of LAD-3 comprised of samples of the SC-D and SC-E phenotypes. **(C)** Plot of first three principal components with ellipsoids showing the general expression space for the sham samples (blue) and the LAD samples (orange) regardless of time point. Points are colored according to the experimental group. This plot demonstrates a reduction in the expression space (diversity) of LAD samples toward an expression pattern that is present in some of the sham samples.

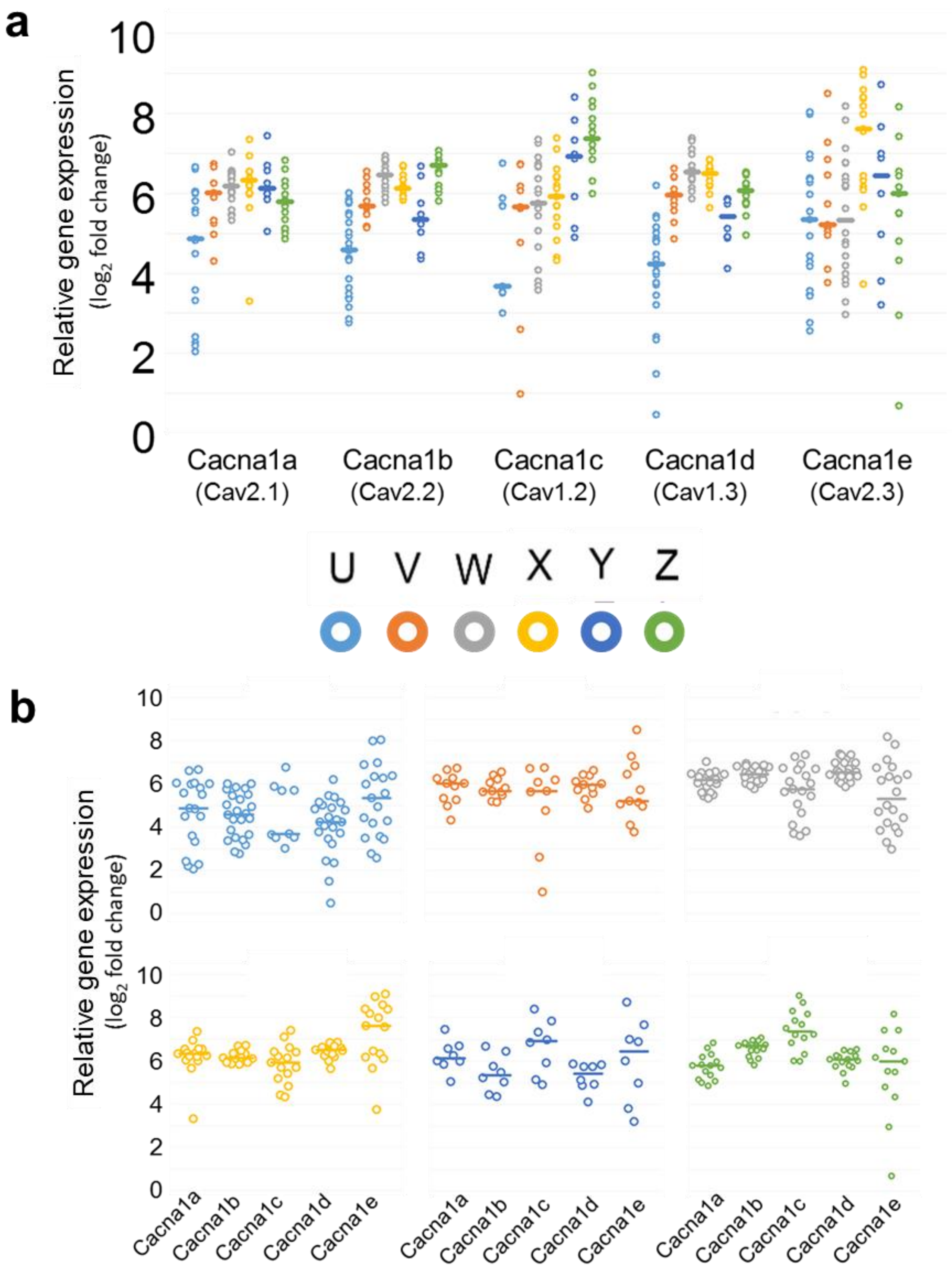

Figure S17: Expression of voltage gated calcium channels across samples clusters show some distinct patterns that may hint at functional behavior, related to Figure 5. (A) Each calcium channel's gene expression values across all sample clusters with each dot representing one sample of pooled neurons. **(B)** Each sample cluster's pattern of calcium channel expression with each dot representing a pooled neuron sample. Both **A** and **B** show the same data points, only grouped differently to aid in making comparisons.

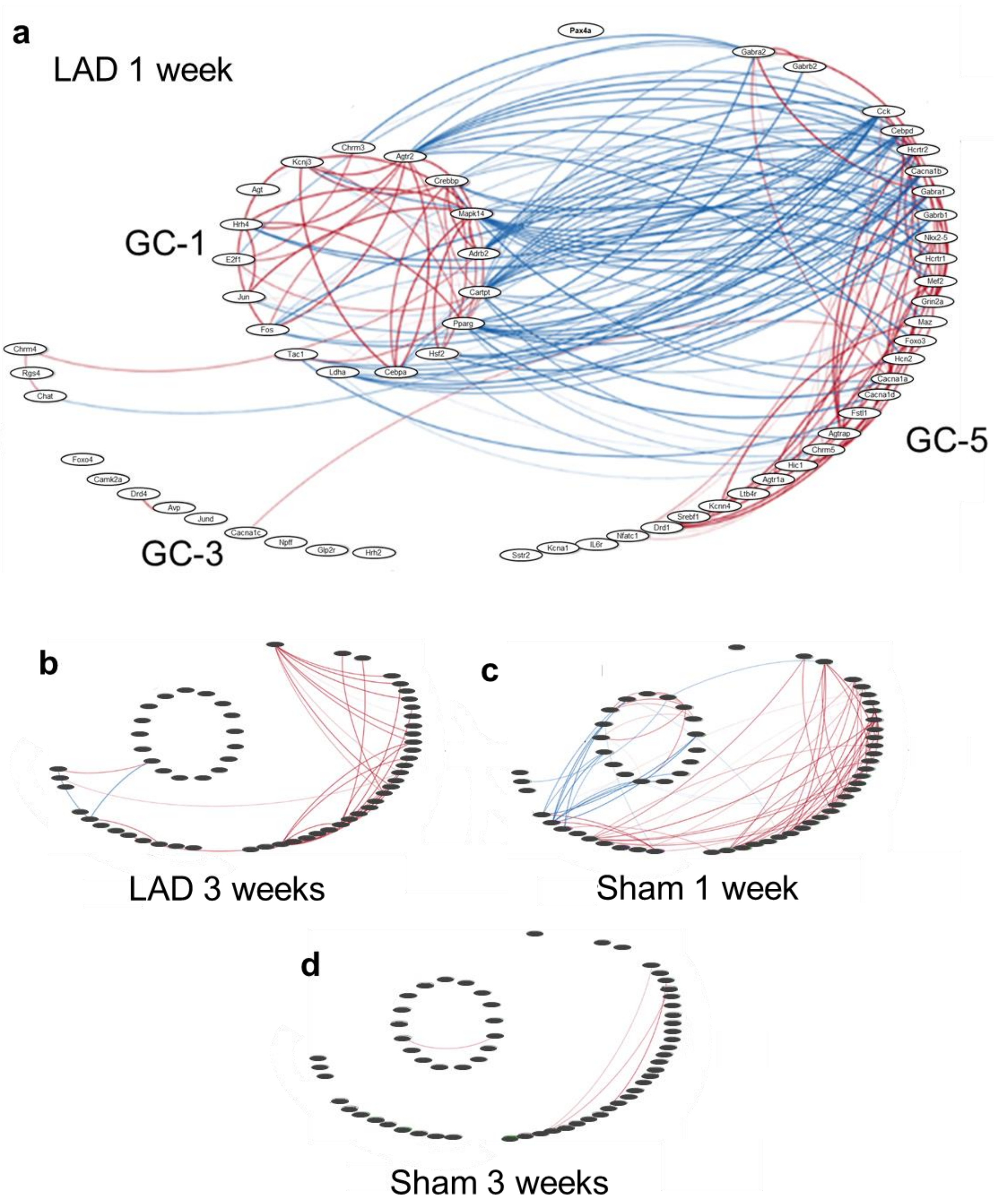

Figure S18: Correlation networks of each experimental group showing only edges that are unique to that group, related to Figure 5. Edges are derived from Pearson correlations with cutoff of $q<10^{-3}$. Blue edges are negative correlations and red edges positive with the edge thickness proportional to the correlation coefficient. **(A)** Network for LAD-1 group and nodes enlarged to show gene names. **(B)** Network for LAD-3 group. **(C)** Network for Sham-1 group. **d** Network for Sham-3 group.

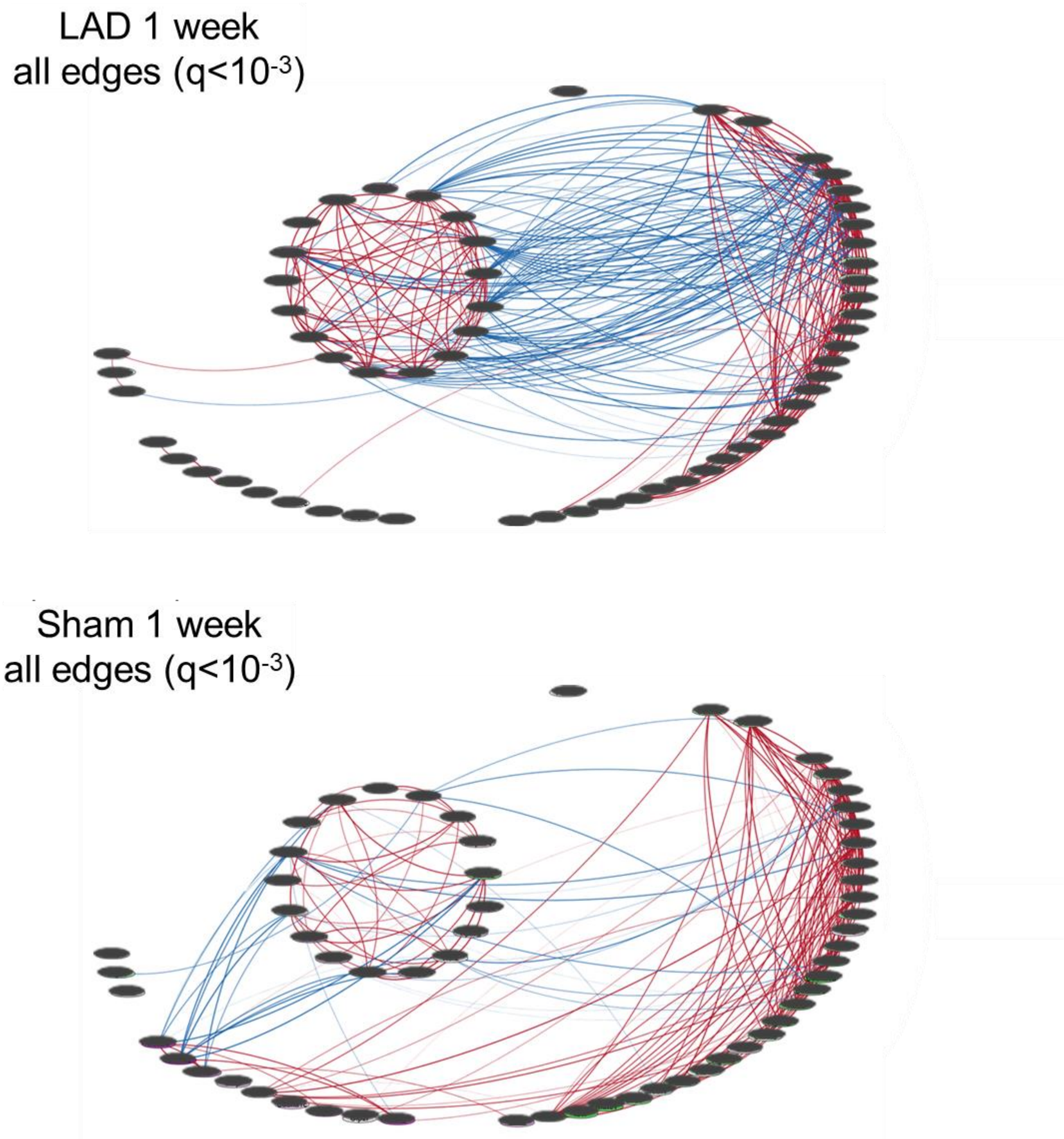

Figure S19: Correlation networks of each experimental group showing all edges, related to Figure 5. Edges are derived from Pearson correlations with cutoff of $q<10^{-3}$. Blue edges are negative correlations and red edges positive with the edge thickness proportional to the correlation coefficient.

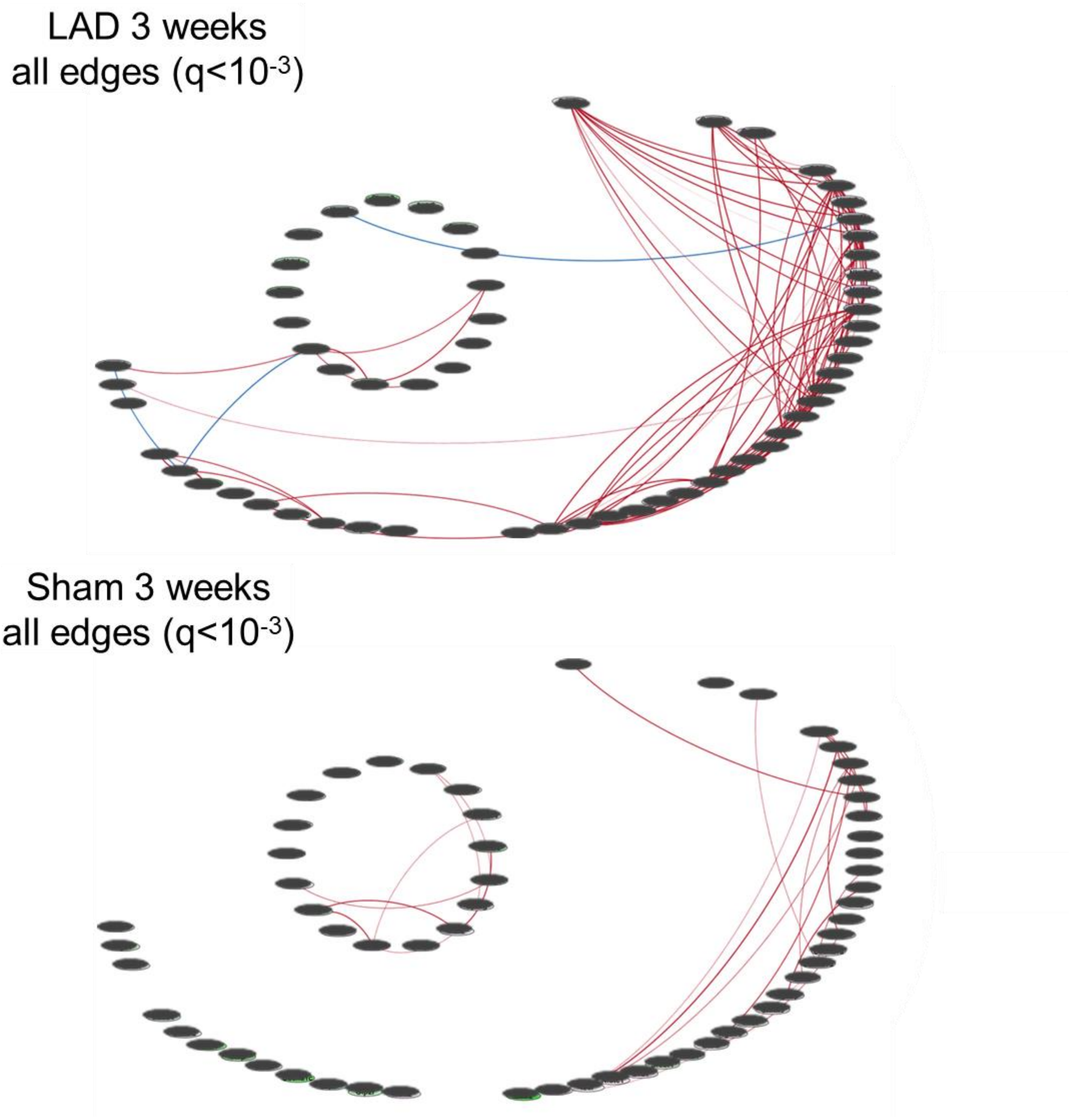

Figure S20: Correlation networks of each experimental group showing all edges, related to Figure 5. Edges are derived from Pearson correlations with cutoff of $q<10^{-3}$. Blue edges are negative correlations and red edges positive with the edge thickness proportional to the correlation coefficient.

LAD1-Sham1
Common Network
Color: LAD1 – Sham1

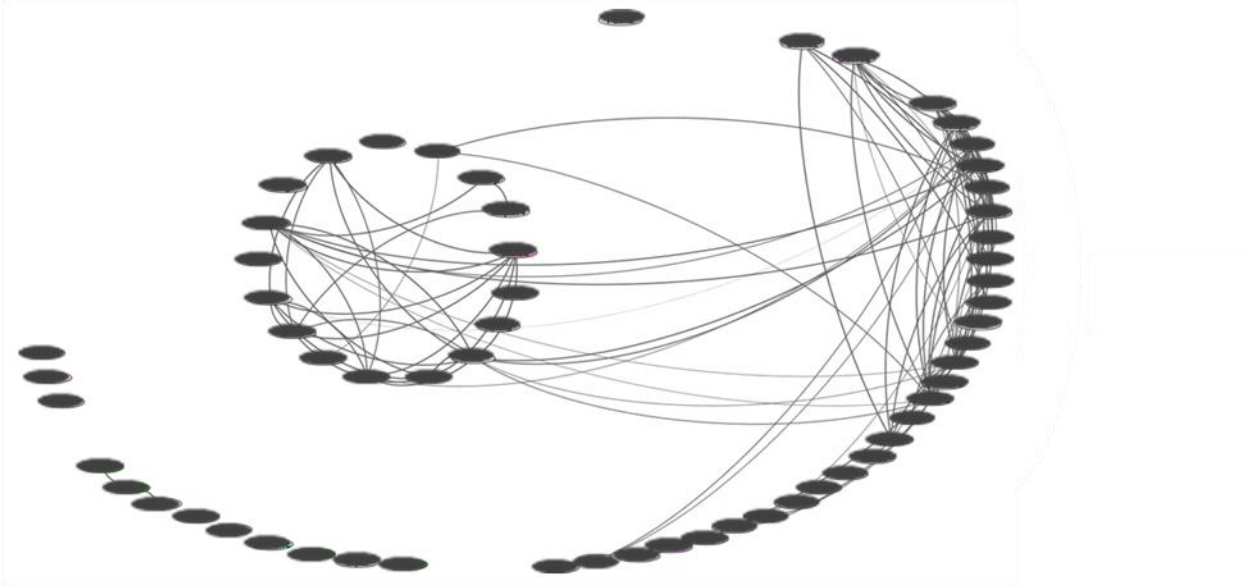

LAD1-LAD3
Common Network
Color: LAD1 – LAD3

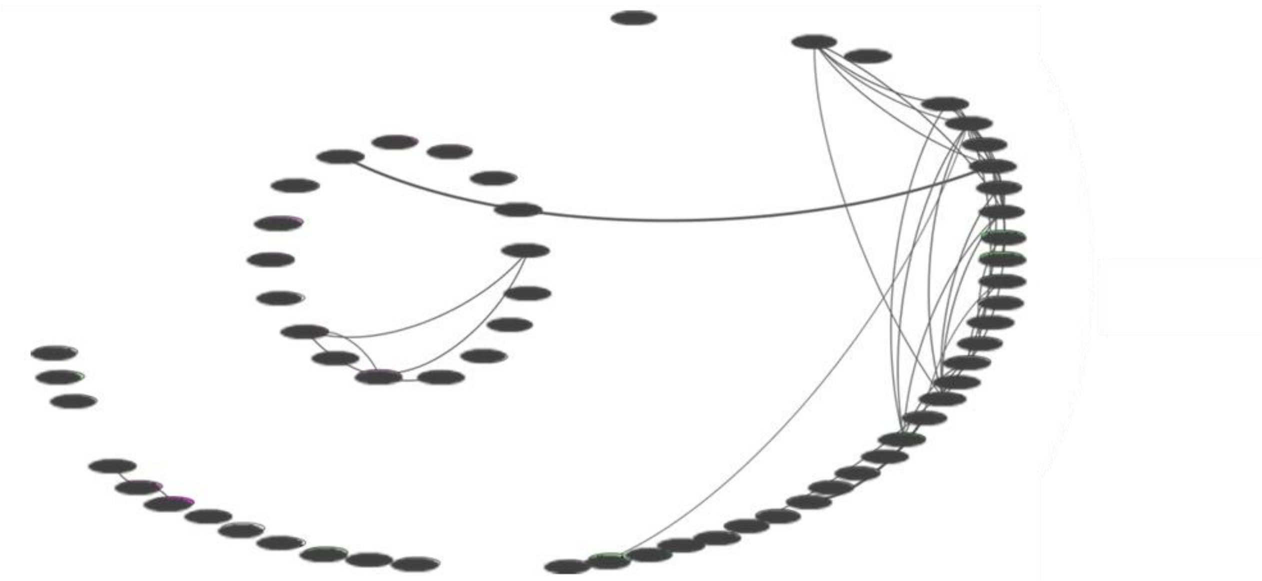

Figure S21: Network representations of common edges between two groups, related to Figure 5. Edge thickness is proportional to the average correlation coefficient. Inclusion of edges are from Pearson correlations with q>0.001.

Gene Cluster	Peptides	Peptide Receptors	Transmitters	Transmitter Receptors	Ion Channels	Transcription Factors	Other
1	Cck<br>Sst<br>Nppa<br>Tac1	Npr3<br>Cckbr<br>Npy4r<br>Oxtr		Chrm1<br>Chrm2<br>Chrm5<br>Adra2a<br>Adrb2<br>Drd1<br>Hrh2<br>Hrh4<br>Htr1b<br>Htr1d<br>Htr1f<br>Htr3b		Cebpa<br>Cebpd<br>Creb1	
2					Scna2		Ldha<br>Gapdh<br>Map2
3		IL6r<br>Ltb4r<br>Oprk1<br>Npy2r<br>Sstr4		Hrh1<br>Chrm3<br>Htr1a	Kcnn4	E2f1<br>Jun	
4	Cartpt	Galr1<br>Grpr<br>Oprm1<br>Sstr2	Th<br>Dbh				
5		Npr2	Gad1<br>Gad2	Adra1a<br>Adra1b<br>Drd2	Cacna1d<br>Cacna1i<br>Hcn1	Hsf2	Rgs4<br>Rpl19
6			Ache	Gabrb2<br>Hrh3	Gabrb2<br>Hcn2<br>Scn1a	Srebf1<br>Foxo3<br>Nr3c1	Actb<br>Eno2<br>Crebbp<br>Agtrap<br>Grk2
7	Npff	Hcrtr1<br>Hcrtr2<br>Thrh		Gabre<br>Gabrq<br>Htr2a	Cacna1h<br>Gabre<br>Gabrq<br>Hcn3<br>Trpm7	Ar	Mapk14<br>Rgs2
8	Fstl1		Chat	Gabra1<br>Gabra2<br>Gabrb1<br>Chrnb2<br>Chrna7	Gabra1<br>Gabra2<br>Gabrb1<br>Chrnb2<br>Chrna7<br>Cacna1a<br>Cacna1b<br>Cacna1e<br>Hcn4		Camk2a

Table S2: Categorization of genes from each of the clusters defined in the heatmap of Figure 1B and 3A. Gene clusters were determined by unbiased hierarchical clustering, as described in methods. There are tendencies for ion channels to be in only a few clusters, suggesting that the milieu of channels expressed is coordinated rather than emergent from a series of diverse inputs.

Surgery	Survival Time	Number of Animals	Number of Neurons	Number of Samples
**Sham**	**1 week**	3	133	19
**LAD**	**1 week**	3	136	17
**Sham**	**3 weeks**	3	158	21
**LAD**	**3 weeks**	4	279	34

**Ion Channels**		**Neuropeptides**	**Transcription Factors**	
Cacna1a	Gabre	Agt	Agtr2	Hsf2
Cacna1b	Gabrq	Avp	Cebpa	Jun
Cacna1c	Grin2a	Cartpt	Cebpb	Jund
Cacna1d	Hcn2	Cck	Cebpd	Maz
Cacna1e	Kcna1	Gal	Creb1	Mef2
Gabra1	Kcnj3	Npff	Cutl1	Nfatc1
Gabra2	Kcnn4	Sst	E2f1	Nfκb
Gabrb1	Trpc6	Tac1	Fos	Nkx2-5
Gabrb2			Foxo3	Pax4a
			Foxo4	Pparg
			Hic1	Srebf1

**G-protein Coupled Receptors**			**Modulators**	**Others**
Adra1b	Drd1	Hrh4	Agtrap	Chat
Adra2	Drd2	IL6r	Camk2	Ldha
Adrb2	Drd4	Ltb4r	Crebbp	
Agtr1a	Fosb	Oxtr	Fstl1	
Chrm1	Glp2r	Sstr2	Grk2	
Chrm2	Hcrtr1	Sstr3	Grk3	
Chrm3	Hcrtr2	SStr5	Mapk14	
Chrm4	Hrh1	Tnfrsf1a	Rgs3	
Chrm5	Hrh2		Rgs4	

Table S3: Experimental design for chronic ischemia including distribution of sample collection and list of genes with categories, related to Figure 5.

Gene Cluster	Peptides	GPCR's	Ion Channels	Modulators	Transcription Factors	Others
GC-1	Cartpt<br>Tac1<br>Agt	Agtr2<br>Chrm3<br>Hrh4<br>Adrb2	Kcnj3	Mapk14	Pparg<br>E2f1<br>Cebpa<br>Hsf2<br>Crebbp<br>Fos<br>Jun	Ldha
GC-2	Sst<br>Gal	Sstr3<br>Oxtr<br>Adra1b<br>Chrm1		Grk3	Cebpb<br>Nfkb	
GC-3	Npff<br>Avp	Drd2<br>Hrh2<br>Tnfrsf1a<br>Glp2r	Cacna1c<br>Trpc6	Rgs3<br>Camk2a	Fosb<br>Foxo4<br>Cutl1	
GC-4		Hrh1<br>Chrm2<br>Adra2<br>Chrm4	Gabrae<br>Gabraq			Chat
GC-5	Cck<br>Fstl1	Sstr5<br>Sstr2<br>Drd1<br>Chrm5<br>Hctr2<br>Agtr1a<br>Ltbr4<br>IL6r<br>Hctr1	Cacna1a<br>Grin2a<br>Gaba2a<br>Cacna1b<br>Gabrb1<br>Kcnn4<br>Gabrb2<br>Cacna1d<br>Gabra1<br>Hcn2<br>Kcna1<br>Cacna1e	Agtrap<br>Grk2<br>Rgs4	Pax4a<br>Jund<br>Hic1<br>Mef2a<br>Foxo3<br>Nkx2-5<br>Maz<br>Cebpd<br>Nfatc1<br>Srebf1<br>Creb1	

Table S4: Categorization of genes from each of the clusters defined in Figure 5A.

Transparent Methods

Animals

This study used adult male Sprague Dawley rats in the weight range 250-300g (Envigo). All animal experiments were carried out in accordance with protocols approved by the Thomas Jefferson University Institutional Animal Care and Use Committee.

Procedure for remote ischemic preconditioning (RIPC)

The remote ischemic preconditioning (RIPC) protocol used here largely mirrors that described by Gourine, Mastitskaya and colleagues (Mastitskaya et al. 2012; Basalay et al. 2016) with all protocols being approved by the Thomas Jefferson University Institutional Animal Care and Use Committee. Adult 250-300g male Sprague Dawley rats (Envigo) were given a single intraperitoneal injection of ketamine/xylazine (100 mg/kg and 10 mg/kg) and then subjected to either a sham surgery or the RIPC protocol. Both surgeries involve bilateral small incision in the upper thigh, isolation of the femoral vein and artery from the nerve in the femoral sheath, and placement of a suture thread around only the vessels. In the RIPC procedure the thread was tied to occlude the vessels, being left untied in the sham, for fifteen minutes. The suture was removed and the limbs permitted to reperfuse. Both the ischemia and reperfusion were confirmed through visualization of blood flow and pallor followed by subsequent recoloration of the surrounding muscle tissue. The animals survived for two and half hours further before sacrifice. During this survival period, the animal was placed on an isothermal heating pad and the wounds in the lower limbs covered with gauze pads soaked in sterile saline. For heart stained with 2,3,5-triphenyltetrazolium (TTC), methods were followed similar to those outlined in prior work (Redfors et al. 2012). In brief, post-mortem hearts were sectioned at a thickness of ~5mm and stained using neutral TTC for 20 minutes and then stored in phosphate buffered saline for 12 hours prior to imaging. The timing for each survival period is as shown in Figure 4B. These methods apply to data shown in Figures 3, 4, 6 as well as Supplemental Figures S5-S12.

In vivo manipulations - chronic ligation of the left anterior descending artery (LAD)

All experiments were performed with the approval of the Institutional Animal Care and Use Committee at Thomas Jefferson University. All procedures and sacrifices were done within the same four hour circadian period. Male Sprague-Dawley rats (250-300g) were anesthetized with ketamine (100mg/kg) and xylazine (10mg/kg) and intubated with an 18g venous catheter and the animal was ventilated with oxygen-enriched room air using a rodent ventilator (Harvard Apparatus). A left thoracotomy was performed using aseptic technique and the heart was exteriorized. The left anterior descending (LAD) artery ligation was performed by passing a 6-0 prolene cardiac suture around the LAD artery and tying it off. Paling of the myocardium was observed before the heart was replaced back into the thoracic cavity and the thoracotomy closed with 4-0 silk suture. The sham ligation procedure involved the same preparations and left thoracotomy, but instead involved passing the 6-0 suture and needle around the LAD artery without tying the suture. The animals were then extubated and allowed to recover on a heating pad for four hours before being transported back to the holding facility where they were maintained in a 12h light-dark cycle and given *ad libitum* access to food and water. Animals were monitored post-surgically every 24 hours for signs of distress or improper healing and removed from the study if any such signs presented. These methods apply to data shown in Figure 5 as well as Supplemental Figures S13-S21. For the data shown in Figure 4, the RIPC methods described

above as well as LAD ligation as described here were used, except with survival times as described in the figure legend.

Animal Sacrifice and Harvesting of Brainstem

After the appropriate survival period (1 to 3 weeks for LAD/Sham experiments, 2.5 hours for RIPC experiments), each animal sacrificed by rapid decapitation that was preceded by brief 60 seconds of 5% isoflurane in O_2. The brain was quickly removed and placed into ice cold artificial cerebrospinal fluid for separation of the brain stem that was then rapidly frozen in optimal cutting temperature (OCT) medium for cryosectioning. The heart was removed and rinsed in cold PBS before being rapidly frozen in OCT. No more than ten minutes passed between decapitations and freezing the tissue in OCT for each animal.

Processing of Samples for LCM

The brain stems were sectioned at 10µm and collected on SuperFrost Plus glass slides in preparation for laser capture microdissection (LCM). Neurons in the DMV were identified by use of a rapid (~15 minutes) immunofluorescence labeling procedure staining for NeuN. We have employed this method in our previous studies (e.g., Park et al 2014). In brief, high concentration of primary NeuN with alternative organism Fc binding to a secondary fluorescent antibody is performed followed by a rapid dehydration process involving increasing ethanol concentrations with two xylene washes prior to LCM. A DAPI stain is also included along with the secondary antibody to provide an opportunity for visualization of the nucleus. Such methods have been shown to adequately stain high-abundance proteins like NeuN for the purposes of cell identification while maintaining the quality of mRNA. Single neurons and pools (composed of 2-5 neurons) were collected from the DMV of adult male Sprague Dawley rats (N=3 animals) in the region coextensive with the area postrema using the Arcturus HS Capsure system, which involves use of an infrared laser to bend a membrane on a cap to contact tissue on a slide and isolate it for downstream processing. Each Arcturus cap containing neurons was visualized after capture to ensure that only neurons were included in the samples. Any cap containing non-neuronal cells were not used in the study. Apart from collecting neurons only, some samples were collected from the same slides that included the entire area of the DMV represented on that slice. The samples for this study were all collected from the part of the DMV that begins at the obex and runs caudally for 500µm.

Rationale for RT-qPCR

Morphological studies on DMV neurons have suggested for some time that there is some heterogeneity within the population (Gao et al. 2009; Fox & Powley 1992). Such work has led to uncovering of multipolar peripherally projecting efferent neurons, multipolar GABAergic interneurons, and a population of unipolar neurons (Rinaman et al. 1989). It has also been shown that there is some organization within the nucleus as to the efferent vagal targets with interneurons interspersed (Fox & Powley 1992). To date, there has been significant work attempting to identify the molecular biology that underlies DMV function, but through examination of only a handful of transmitters or peptides at a time. Many of the co-labeling studies that were performed found some populations of dual labeled cells, suggesting that co-expression of essential neurotransmitters or receptors is more the rule than the exception, a concept that will be further developed in the work that follows. This work aims to understand the molecular heterogeneity of DMV neurons through broad examination of gene expression. In order to reliably assay gene expression at the level of single cells, it was decided to take a targeted multiplex RT-qPCR approach rather than RNA-seq. In the majority of single cell RNA-seq experiments, it is very

difficult to detect low levels of expression in a reliable and repeatable way, often requiring reliance on the gold standard of PCR to confirm essential findings. Further, it is often the case that a cohort of a few hundred genes are found to be differentially expressed and even less than that to find biological relevance. Our approach considers a targeted set of 100-200 genes that are highly likely to have functional implications to DMV effector function, based upon the body of published literature accumulated to date on DMV in a variety of contexts and disease states (Table S1). Until the sensitivity and specificity of RNA-seq for single cells or larger scale targeted gene expression techniques like MER-FISH are refined, any approach will inevitably miss something, whether through technical limitations or incomplete target gene selection. Our approach here provides data with very high fidelity from which analysis and reasoning can be carried forth with confidence.

Selection of genes for RT-qPCR

The multiplex RT-qPCR experimental design included genes, which are listed in Tables S1 and S3. The selection of these genes from the thousands expressed in the DMV was accomplished through inference based on our previous work in the dorsal vagal complex and through analysis of publicly available transcriptomic studies. There is, however, one dataset examining the DMV in human patients with Parkinson's disease (PD) that may be helpful in understanding DMV pathology (GDS4154). Through use of ANOVA and template-matching, several genes that were differentially expressed in PD patients and only changed in the DMV were considered in constructing the gene list for this current work. Further justification for gene assay selection can be found in the Table S1.

High throughput RT-qPCR

The gene expression was measured in each sample using multiplex RT-qPCR via the Biomark system from Fluidigm (Park et al. 2014). These samples were randomly allocated to three different microfluidic chip multiple qPCR runs along with samples across treatment groups to minimize technical noise across sample types. For naive animals 84 samples passed quality control composed of 60 single cell samples and 24 cell pools totaling 156 cells in all. 106 samples passed quality control comprising 45 RIPC and 61 sham from N=3 animals for each cohort. Pooled single cell samples were prepared for multiplex real-time quantitative polymerase chain reaction (RT-qPCR) with the Biomark HD system using VILO III (ThermoFisher) reverse transcription of RNA into cDNA followed by 22 cycles of pre-amplification (Taqman Pre-amp Master Mix) of the cDNA using the collection of primers that were utilized in the multiplex RT-qPCR. Each set of primers was designed to span introns when possible and was validated through *in silico* PCR (Primer BLAST, NCBI) and observation of a single band with the expected amplicon size on PCR gel using standard rat brain RNA (Park et al. 2014). The quality of the results of the multiplex RT-qPCR was ensured through examination of the melt curves and standard curves. If there were multiple peaks for the melt curves or if a reliable standard curve was not present, the assay was not used in further downstream analysis. Similarly, if a sample showed that more than half of the assays were below the limit of detection, the entire sample was not utilized in downstream analysis. Similar techniques were utilized for the chronic ischemia experiments with the gene assays and numbers enumerated in Table S3.

Normalization and Handling of Missing Information

Raw C_t values from the multiplex RT-qPCR that passed melt curve based quality control were first median centered within each sample in order to account for the variations in total RNA in the sample to get delta C_t values. For analysis and reporting purposes, negative delta C_t ($-dC_t$) values

were used because a lower C_t value is representative of a higher expression level. By using -dC_t values, higher values now represent higher gene expression and are normalized to account for different initial total RNA amounts. One difficulty with assaying single cells in general is the large number of assay-sample combinations that end up having values below the limit of detection, resulting in a large number of null or not applicable (NA) values. Most of the algorithms used to cluster across several measurements omit NA values or impute them. Omission of NA values may lead to an over-estimation of expression for a given group or cohort due to not counting NA values as being of very low to zero expression. They are instead considered to be missing information. In this case, the most likely reason for an NA value is very low to no expression of a certain gene which is different from uncertainty about its expression. Similarly, imputing NA values when there is such a large number will likely introduce patterns that are overfit from the non-NA data. In order to work around this, a technique of binning the data was utilized solely for the purpose of determining membership in clusters/phenotypes. Five bins were used with the lowest being composed of all NA values and the remaining four being the quartiles of the remaining non-NA values. This method permits the clustering algorithms to use the information from a lack of expression in the calculations and generate a more accurate membership in a cluster for each sample accordingly. For the reporting of data and any associated statistics, expression values in the form of negative delta Ct values (centered on the sample median of the top 35 expressed genes) are used along with the percentage of samples in a given group that have expression values above the limit of detection. For bar plots, expression values are recentered as a whole such that no value is below zero, making them visually easier to interpret. This also effectively makes zero the limit of detection and the scale of log2 relative expression where a difference of 1 indicates a two-fold difference in expression value and a difference of 2 indicates a 4-fold difference, and so on.

In order to statistically compare groups, it was necessary to consider both the expression value measures of central tendency (means and medians) as well as the number of samples that had a value above the limit of detection. As mentioned previously, the NA values that normally are ascribed to data points where there is uncertainty in the expression value due to technical problems or very low expression appeared to come primarily from very low expression in this set of experiments. This can be seen in the presence of primer dimers in the qPCR melt curve analysis in samples that otherwise have clear signal in more than 25% of all assays. The presence of primer dimers suggest that the reaction chemistry is proceeding appropriately, but there is insufficient template to amplify. When samples that had globally low signal across all assays were removed, the NA values that remain are far more likely to result from being below the limit of detection rather than technical problems. It is this line of reasoning that led to the use of NA values as data the analyses conducted here. It should be noted that a high number of NA values are not generally handled well in dimensionality reduction, even with imputation and ascribing an actual value to the NA based upon a fixed distance below the lowest -ΔCt value introduced a regularity in the data that doesn't scale accordingly with the potential for washing out the signal.

Grouping of Samples into Neuronal States

The methods utilized for segregation of samples into neuronal states for the heatmaps Figure 1 utilized the quartile data as described above as processed by a standard hierarchical clustering algorithm, in this case run through Multiple Experiment Viewer. The dendritic tree that is produced can be used to group the samples into clusters through designation of a tree height cut-off. In this case, the tree height cut-off was determined by the grouping that most parsimoniously segregated the groups without generating groups of n=1. This means that the most groups were generated without a single sample segregating out alone. For Figures 1-3, the grouping that met this criteria included 6 groups. The groups made from the naive DMV neurons in Figure 1C were used for all

of the other downstream figures, including Figure 3, which included data from the RIPC and sham surgery groups.

For purposes of heat map visualization for Figure 5, the negative delta Ct values (normalized by sample to adjust for starting RNA content through centering on median gene expression value), can then be centered or normalized within each gene. Normalization may be done through calculation of a Z score for each value within a gene, giving the number of standard deviations from the mean (e.g. Z=1 is one standard deviation above the mean and Z=-2 is two standard deviations below the mean). This was the approach taken to visualize the data in Figure 5. As mentioned previously, all statistics were performed on the negative delta Ct values, not the gene centered or normalized values.

Other Statistical Analysis

In order to find statistical differences between treatments and over time in the chronic ischemia model work, a mixed linear model ANOVA, aggregating the pooled single cell samples up to the animal level, was used with Tukey post-hoc analysis to correct for multiple comparisons. For determinations of gene expression correlation, each sample was taken to be independent and pairwise Pearson correlations between genes were computed. All statistical calculations were performed using R statistical software.

Explanation of bar graphs for gene expression used throughout the manuscript

The bar graphs showing gene expression data (Figures 1C and S1-S4) have two types of data shown simultaneously, which is necessary for interpretation of the dataset as a whole. With data centered on single cells or small groups of single cells, there is an understanding that the population shown in each bar is made up of many single cells and that these populations are heterogenous. With single cells, even of a similar neuronal state or phenotype, not all cells express the gene of interest at the same level or even at all. Therefore, in order to understand the population for each bar, there are two things that must be known simultaneously: 1) What percentage of the cells express the gene of interest at all and of the cells that express the gene of interest, at what quantitative level? For example, there may be a population of cells that all express gene X at a low level and a different population where very few cells express gene X, but all at a very high level. In only reporting the "average" expression level, these populations may look similar, but the underlying cells that make up the populations have very different implications for how the system works. For this reason, both the percentage of cells expressing a gene are shown and the expression of level of the gene for those with detectable expression are shown. The percentage of cells with detectable expression is shown by the narrow yellow line with the axis on the right. The expression level for the gene in the detectable cells is shown by the full bar with error denoted, colored based upon the neuronal state or experimental group from which the cell population is derived.

Prediction and Testing of microRNA regulators of DMV Gene Expression in RIPC

Given the large cohort of genes that had decreased expression in the RIPC cohort, microRNAs emerge as a potential regulator. Although microRNAs might act on gene expression in a myriad of ways, searching only the 3'UTR of genes increases the chance that the microRNA that binds there plays an inhibitory role in expression of the mRNA target (Fang & Rajewsky 2011). Through combined use of four algorithms, we determined the cohort of microRNAs that most parsimoniously lead to decreased expression, a cohort of 20 genes that were decreased in RIPC. If a microRNA had a putative binding site in the 3'UTR in more than one algorithm, it is shown in

Figure 5A. The algorithms used here were miRWalk (Dweep et al. 2011), RNA22 (Loher & Rigoutsos 2012), Targetscan (Agarwal et al. 2015; Betel et al. 2008), and miRanda (Betel et al. 2008).

Broad assays of microRNA were obtained through the examination of differentially expressed microRNAs in the DMV of male rats. In this study, four conditions were tested: LAD alone, RIPC alone, RIPC followed by LAD two hours later, and age-matched control. Animals were sacrificed and brains were harvested 24 hours after LAD in the case of LAD alone and RIPC followed by LAD and 26 hours after RIPC alone. Animals were sacrificed and brainstems were harvested as described above and were sectioned at 200 μm and the DMV was extracted using a tissue punch 1mm in diameter. Samples were collected and assayed for microRNA content using Nanostring nCounter assay that we have employed previously for brainstem tissue (DeCicco et al. 2015). After normalization and quality control, 146 microRNAs remained with detectable expression. Pavlidivis Template Matching was used to find microRNAs whose expression was perturbed in LAD alone compared to controls, but whose expression returned to the control baseline with RIPC preceding LAD. Current literature on these three microRNAs (miR-218a, miR495, and miR-183) suggest many interactions with ion channels and other excitatory and neuronal processes, supporting our prediction that perturbation of these microRNAs could provide some level of protection to reduce cardiac pathology and malfunction following LAD.

Summary of literature supporting the proposed input-output signal processing map of the DMV

A great deal of effort has been expended to determine the inputs the DMV receives, both from an anatomical as well as molecular perspective. Some of these projections have also been characterized as to their effects on the physiology of DMV targets. In spite of this, connectivity information has not yet been collected into a processing map of the DMV as we have here in Figure 2. While this work has been important in deciphering the role of the DMV in coordinating and mediating CNS signaling. The following is a brief review of literature highlighting the axonal projections to the DMV and the neurotransmitters that are unique to each, therefore generating an input/output map of the DMV upon which the current data can be used to infer potential bandwidth of signaling from each source through receptor expression and effector transmitters and neuropeptides through enzyme and propeptide expression. There is the possibility that this map will require updating as more data becomes available, but this represents the state of knowledge at this current time.

Afferent Projections to DMV

The paraventricular nucleus (PVN) innervates the DMV with excitatory projections that contain combinations of several peptides, including oxytocin (Rinaman 1998; McCann & Rogers 1990), bombesins including gastrin releasing peptide (Costello et al. 1991), atrial natriuretic peptide (Saper et al. 1989), cholecystokinin (Kim et al. 2003), and some sparse dopaminergic fibers as well (Hyde et al. 1996). These projections have been shown to mediate stress, satiety, and cardiovascular behavior. The lateral hypothalamus (LHA) sends projections containing orexins and brain natriuretic peptides (Zheng et al. 2005; Saper et al. 1989). Also from the hypothalamus are histaminergic tuberomammillary projections and synapse prominently in the DMV as well as the NTS (Poole et al. 2008; Green 1978; Bealer 1999; Deng et al. 2010). The raphe pallidus (RPa) also receives projections from the PVN among other places, and sends efferent fibers to the DMV. These efferents are known to express serotonin and thyrotropin releasing hormone (Garrick et al.

1994; Palkovits et al. 1986; Taché et al. 1995). These projections have been shown to mediate both cardiovascular and gastrointestinal functions. The central amygdala (CeA) and bed nucleus of the stria terminalis (BST) send projections to the DMV containing corticotropin releasing hormone, neurotensin, tachykinins like substance P (Gray & Magnuson 1987), and somatostatin (Higgins & Schwaber 1983). There are also a large number of projections from the nucleus of the solitary tract and area postrema that include combinations of glucagon-like peptide 1, GABA, glutamate, norepinephrine, and dopamine (Hyde et al. 1996; Trapp & Hisadome 2011). There are excitatory adrenergic projections from the caudal ventrolateral medulla C1 population that terminate in the DMV (DePuy et al. 2013) and the locus coeruleus (Wang et al. 2014). Afferent cranial nerve nuclei send projections to the DMV including the trigeminal nucleus (Jacquin et al. 1982) and vestibular nucleus (Balaban & Beryozkin 1994), both of which mediate physiological reflex activity of the cardiorespiratory system.

Efferent Projections from DMV

Along with canonical vagal efferent projections, there are some efferents that stay within the CNS, projecting to the parabrachial nucleus, cerebellar cortex, and deep cerebellar nuclei (Zheng et al. 1982). There is also a population of interneurons, described as GABAergic, that appear to project locally within the DMV as well as into the NTS (Gao et al. 2009). Still the vast majority of DMV projections that leave the dorsal vagal complex run within the vagus nerve out to the periphery, innervating most major organ systems. Most prominent are gastrointestinal projections encompassing between 80-90% of all DMV-derived vagal efferent axons (Fox & Powley 1985; Jarvinen & Powley 1999; Gao et al. 2009). It should be noted that there are still a substantial number of axons that project to the intracardiac ganglia as well, about 5% of all DMV vagal efferents (Cheng et al. 1999; Cheng et al. 1997), roughly equivalent to the number of axons that originate from the NA (Standish et al. 1995). Since the molecular understanding of such projections has been derived largely from pharmacological studies, a strong characterization of the peptidergic signaling targets has not been well-defined. It is known that DMV neurons express several neuropeptides, along with acetylcholine, dopamine, and nitric oxide. However, the target specificity of such projections still remains to be fully characterized. Some work toward this has described specific dopaminergic DMV projections to the pancreas (Loewy et al. 1994), catecholaminergic projections to the gastric fundus (Guo et al. 2001), and nitric oxide, vasoactive intestinal peptide, and nicotinic receptors on gut macrophages that are signaled via the vagus nerve (Cailotto et al. 2014).

Summary of literature for interpreting the neuromodulatory functions of DMV neurons

Gad+ DMV Neurons

Almost all Gad+ single neurons express the canonical glutamatergic gene Camk2a (Liu & Murray 2012) at some level. There is ample evidence for the existence of neurons that are simultaneously GABAergic and glutamatergic, even releasing both from the same terminals at the same time (Fattorini et al. 2009; Jarvie & Hentges 2012; Danik et al. 2005; Boulland et al. 2009; Zander et al. 2010; Gutiérrez 2005; Ottem et al. 2004). While this has been described at multiple regions of the rat brain, this is the first evidence we are aware of for the presence of such neurons in the dorsal motor nucleus of the vagus. It is surprising that nearly all neurons that express one or both of the glutamate decarboxylase genes co-express Camk2a. Almost all of the single cells assayed here express Camk2a, with just less than half expressing Gad1 or Gad2. The implications for this are not entirely clear, but it suggests that it is likely true that GABAergic neurons do project into

the periphery, given that almost half of all single cells assayed at random can produce GABA. Given the plasticity that our work from the DMV in heart failure suggests, it may also be the case that the ability to generate GABA may come and go based upon the milieu of inputs driving these neurons. Further, GABA has been shown to stimulate release of GLP-1 (Gameiro et al. 2005), cholecystokinin (Jansen et al. 2000), 5-HT (Schwörer et al. 1989), and gastric acid (Xu et al. 2001) in the gastrointestinal system. In Gad+ neurons, there are several that co-express Chat as well. There is a negative correlation between Chat and both Gad1 and Gad2 across all Gad+ cells. This suggests a gradient in the expression of these genes that may indicate a gradient in the ability to co-release GABA and acetylcholine. The presence of such a gradient lends credence to the ability of these neurons to switch from one phenotype to another.

This analysis suggests that there are distinct transcriptional states of GABAergic neurons in the DMV (Figure S5). While four clusters are identified here, it is likely that some of these clusters may represent neurons of a similar state, but with different transient behaviors that might be further defined by dynamic studies. For purposes of this initial discussion, it is assumed that the four clusters represent four distinct functional states. There are several genes that show enriched or suppressed expression levels unique to each cluster and these may give clues to what makes each phenotype unique (Figures S6-S8). For G_A the calcium channels Cacna1a and Cacna1i are suppressed along with Rgs2. Nppa expression is restricted almost entirely to G_A and Chrm5 and Kcnn4 are heavily enriched in G_A. For G_B, the most relatively suppressed genes include Gad1, Htr1a, and Kcna1. More notable are the enriched genes Chat and Fstl1 that are expressed in almost all neurons sampled from G_B and almost unexpressed in G_C. Also of note for G_C is the suppression of Adra1b. G_C is enriched for Gad1 and Scn1a. While Cacna1i is suppressed in G_A, it is very strongly expressed in G_C and G_D. Of all the other genes uniquely expressed in G_D, most have no representation in G_C. This includes Adra1b, Fos, and Npff. Of note as well are Cacna1g and Dbh, both of which are starkly increased in G_D.

It may be possible to infer functional distinctions from each of the Gad+ clusters by contrasting their gene expression patterns. The most prominent feature of G_A is its unique expression of Nppa, which codes for the atrial natriuretic peptide (ANP), a protein heavily enriched in cardiac myocytes that is released to signal increased pressure within the heart (Adler et al. 2013) (Figure S6). One of the other clusters, G_D, has enriched expression of the ANP receptor Npr2. Npr2 is also expressed in cardiac neurons as well as enteric neurons. Also of interest is the high expression of Kcnn4, the mediator of the I_K potassium current that generally acts to maintain a hyperpolarized state. It may be that the hyperpolarized tendency is meant to increase the barrier to fire an action potential, requiring a strong depolarization to fire. Taken together, the GABAergic neurons of G_A are hypothesized to project out the periphery. They lack several calcium channels characteristic a burst or tonic firing phenotype and the expression of Nppa makes these neurons enticing as effectors in the periphery as does their enhanced potential for responsiveness to peptides like somatostatin and cholecystokinin based upon relative receptor expression.

Characterization of G_B includes unique downregulation of Htr1a in favor of Htr2a, representing a shift from an inhibitory response (G_i) to serotonin to an excitatory response (G_q). This comes along with a uniquely high expression of Chat and Fstl1 (Figure S7). In some parts of the brain, cholinergic and GABAergic neurons are distinct populations (Adler et al. 2013), yet they have been shown to also potentially be one in the same (O'Malley & Masland 1989). Fstl1 codes for the protein follistatin-1, which apart from its well-described role during embryological development has been shown to exhibit robust cardioprotective effects against hypertrophy and ischemia-reperfusion injury, even so far as to induce myocyte regeneration in mammalian heart tissue (Ogura et al. 2012; Wei et al. 2015). Follistatin-1 is normally secreted from cardiac myocytes, but in the face of ischemia and myocyte death, this secretion is significantly reduced by the dying

myocytes with others increasing their secretion (Hall 2014). Some evidence exists for replacement of follistatin-1 as an exogenous cardioprotective agent (Ogura et al. 2012). While this may make expression of Fstl1 a likely candidate for one of the mediators of the cardioprotective effects of the DMV, there is no significant upregulation observed in the RIPC group. Although other work done in the DMV on a longer time course for heart failure does indeed demonstrate a substantial upregulation primarily three weeks after infarct and much less so at one week post-infarct. The high expression of Chat and Fstl1 make the GABAergic neurons of G_B another attractive candidate for projecting motor neurons from the DMV to the periphery. The other two subtypes defined here have qualities that are suggestive of a role as integrative interneurons rather than projecting effectors.

Perhaps more like canonical GABAergic neurons, G_C neurons (Figure S7) have very low expression of Chat coupled with high expression of Gad1 and Scn1a, the latter of which has been shown to be essential for action potential firing more specifically in GABAergic neurons although it is present in several neuronal subtypes (Cheah et al. 2012). The high expression of Cacna1i is suggestive of a neuron that is capable of rebound burst firing at hyperpolarized resting membrane potentials (Lee et al. 2014; Andrade et al. 2016; Cain & Snutch 2013; Snutch et al. 2013). This may suggest G_C neurons to be the most likely candidate of the four to be tonic firing interneurons although a great deal more work will be needed to verify this supposition.

Much like G_C, neurons in G_D also express higher levels of Cacna1i along with uniquely expressing high levels of Cacna1g, another low voltage gated T-type calcium channel implicated in burst firing at low resting membrane potentials (Cain & Snutch 2013). Unique to this cluster is a larger percentage of neurons expressing higher levels of Adra1b, Dbh, Npff (Figure S8). The α_{1B} receptor subunit encoded by Adra1b has been associated with susceptibility to synucleinopathy both in over-expression models as well as in *in vivo* studies (Papay et al. 2002; Greene et al. 2005). While the implications of this are not clear at present it bears mentioning given the viable, yet debated, Braak hypothesis that places the DMV at the forefront of Parkinson's disease pathology preceding pathology in the substantia nigra (Braak et al. 1999; Dickson et al. 2010; Burke et al. 2008). All subtypes of GABAergic neurons examined here express some collection of adrenergic receptors, suggesting their ability to be influenced by projections from A2 neurons of the NTS or C1 neurons of CVLM among others. However, the more unique expression Adra1b in lieu of Adra1a, which is more highly expressed in G_B and G_C, may suggest a different response to adrenergic stimulation. The α_{1A} favors mediation of calcium influx whereas α_{1B} favors generation of inositol triphosphate as a second messenger (Bylund 1992). As these neurons are effectively the only GABAergic neurons that also produce NE (due to Dbh expression) and Npff, they may be considered to be more sympathetic-like rather than parasympathetic-like.

There are two potential implications for neurons reliably expressing low-voltage activated T-type calcium channels (especially G_C and G_D that will be considered here. One is that they phenotypically maintain a low resting membrane potential so that they might flux calcium without firing action potentials. Such "subthreshold" calcium fluxes have been associated with a secretory phenotype where the oscillations are associated preferentially with peptide release rather than neurotransmitters (Cooper et al. 2015; Mikoshiba 2007; Yang et al. 2018; Rogers et al. 2011). However, it is also possible that the burst firing serves to aid in network synchronization as is the case in thalamic relay networks (Cain & Snutch 2013). Through maintaining large windows of depolarization with windows of hyperpolarization, the "decision" to fire an action potential requires synchrony of inputs. This latter role for burst firing provides an excellent control concept for nucleus that has many neurosecretory cells with pacemaker activity (Goldberg et al. 2012; Cooper et al. 2015). This may provide one source by which vagal withdrawal is mediated, especially if the

putative inputs from A2 and C1 cell populations are considered. These are neurons that are known to mediate sympathetic effects in their downstream targets in the periphery. If the sympathetic effectors fire with certain patterns, it is possible that they can activate GABAergic interneurons that might modulate DMV secretomotor neurons. This modulation does not necessarily mean inhibition, as a lowering of the resting membrane potential of neurons can both inhibit action potential firing as well as encourage subthreshold calcium flux, the latter of which may mediate peptide release in a secretory fashion.

The presence of GABAergic cell bodies in the DMV was once up for debate, but with improving techniques for protein detection and gene expression sensitivity, their presence has been confirmed (Fong et al. 2005; Le Brun et al. 2008). There still exists a debate whether these GABAergic neurons act as local inhibitory interneurons, project out to the periphery, or both. With the characterization begun with this study it may be possible to define markers of projecting GABAergic neurons versus GABAergic interneurons, especially in light of the evidence mentioned previously for neurons that are a hybrid of canonical excitatory and inhibitory phenotypes. This concept and the evidence presented here with the blurred lines of distinction between GABAergic neuron subtypes strongly suggest a transcriptional plasticity of “terminally” differentiated neurons that is only beginning to gain broad acceptance.

Fos+ DMV Neurons

One of the underlying hypotheses of this study had been that the cardioprotective effects are being driven by changes in the activity of certain neurons in the DMV. Fos has been shown to be a reliable metric of recent neuronal activity (Bullitt 1990) and serves to identify neurons that were active within the RIPC effect timeframe.

Upon further examination, it may be the case that Fos that does not accurately account for subthreshold ion flux activity nor for activity that does not result in changes mediating long-term-potentiation (Kawashima et al. 2014). At least in the neurons sampled, there were no appreciable differences in the RIPC cohort as compared with the sham (Figure S9). This may indicate that the effect is mediated by activity in such a small number of neurons that the sampling performed here was inadequate to detect them. More likely, however, this is due to the cardioprotective effects being mediated by changes in the molecular identity, like peptide expression, of the neurons that did not drastically alter activity. Another possibility is that the “activity” is not composed of strong depolarizations, but rather subthreshold fluxes. Regardless of the cause, this broadened the search to include all neurons sampled rather than just the Fos+ ones.

Effects of sham surgery on shifting DMV neuronal states

A surprising finding in this work was the prominent effect that the sham surgery had on gene expression in neurons of the DMV, even after just two and half hours. This is exemplified in the distribution of samples in the clusters shown in the heatmap in Figure 3. Several clusters differed from baseline in their sample distribution, most prominently State-s A2, C and D. Both A2 and C have an over-representation of sham samples whereas D has an over-representation of RIPC samples. The main differences from A1 to A2 can be found in parts of gene clusters 1,2, and 7 (Table S2). The differences between the otherwise similar C and D clusters can be found mostly in gene cluster 1, the implications for which will also be discussed more specifically. In brief, they derive from RIPC neurons having expression patterns more similar to naive samples rather than sham, suggesting a repression of the stress response seen in sham. Given the projections to the

DMV from several brain regions that are responsive to pain or stress, including PVN and CeA. The ability for certain neurons to change gene expression patterns so rapidly is indicative of their plasticity even as they respond to a brief surgical procedure under anesthesia. When considering the effect of remote ischemic preconditioning, one of the noticeable features is an absence of the injury effect seen in sham, but not in the RIPC or the naive samples. There are numerous ways to generate a cardioprotective response, including pain and surgical stress (Cheng et al. 2017). However, others have reported a far more significant effect from peripheral ischemia as compared to a sham surgery as we performed in this study (Mastitskaya et al. 2012).

There is a large effect from the surgical treatment, observed in both RIPC and sham cohorts and not naive or observed uniquely in the sham cohort in the Fos+ samples (Figure S9). The most prominent is the significant upregulation of Th in samples from the RIPC and sham cohorts (ANOVA, $p<0.001$). It is only for Fos+ samples, the patterns of Th expression differ when all samples are taken into account as will be discussed.

The Sham response genes uncovered here include Cacna1g, Cckar, and Agtr1a. The definition of differential expression in this study includes a combination of either binomial testing ($p<0.001$) in assaying the percent of samples above the limit of detection and ANOVA ($p<0.05$ with Tukey post-hoc correction) for expression within the samples above the limit of detection (Figure 3A).

It is unclear what caused the increase in the low-voltage gated calcium channel Cacna1g (Cav3.1) in this context. It is known that this low conductance channel is involved primarily in pacemaking, calcium oscillations, and burst firing as are the other T-type channels (Perez-Reyes et al. 1998). Since the other T-type channels did not change in response to either sham or RIPC treatment, it suggests that the firing patterns of these neurons will be different from the unchanged counterparts, but the nature of this change must be left to electrophysiological interrogation. The upregulation of Agtr1a and Cckar are associated with generalized stress responses and sympathetic drive in other contexts although their role in RIPC have not been described (Kim et al. 2003; Rinaman 1999; Abegaz et al. 2013; Black 2002). Such signaling in the DMV likely has its source in the NTS and CVLM with CCK signaling also potentially originating from neurons in PVN (Herbert & Saper 1990). Indeed there is evidence for an upregulation of CCK production in PVN neurons in response to stress (Kim et al. 2003). Using template matching, the genes that are most closely expressed in a similar pattern with Cckar include Th, Galr1 (galanin receptor 1), and Sstr2 (somatostatin receptor 2). Galanin signaling may also come from the NTS, but may also include signaling from the central amygdala (CeA) and bed nucleus of the stria terminalis (BST) (Herbert & Saper 1990). Somatostatin projections have their origins most likely in the CeA and BST (Higgins & Schwaber 1983; Schwaber et al. 1982), a region well known to be one of the coordinators of stress in the CNS (de Kloet 2002). Galr1 has been shown to mediate stress responses in Th expressing neurons of other brain regions, like the locus coeruleus (LC) and may have similar effects in the DMV (Kuteeva et al. 2008). Delivery of galanin directly to the DMV has the net effect of causing hyperpolarization in gut-related DMV neurons (Tan et al. 2004). The upregulation of Hrh3 also argues for decreased excitability owing to its ability to reduce the release of Ach and other neurotransmitters through presynaptic activity albeit without an appreciable difference in excitability (Deng et al. 2010; Poole et al. 2008). Taken together, there is an overall sense of inhibiting normal gut function in the surgical response neurons as inferred from these changes, but that does not mean that the neurons modulating molecular changes in the gut, like secretory function, are also inhibited.

Interestingly, the neurons that have Agtr1a upregulated tend to coexpress Cacna1g. While the pattern of Galr1 has some overlap with these samples, Th, Sstr2, and Cckar do not. This suggests that there may be two distinct neuronal subtypes driving the surgical response. Instead, Cacna1g

and Agtr1a have patterns of expression more similar to Hrh3, Hcn2, and Npff. Hrh3 is one of the genes that is diminished in RIPC back down to naive levels. Npff has the ability to generate a positive feedback loop inducing its own expression and activating neurons in an autocrine and paracrine fashion in the autonomic control regions of the brainstem (Jhamandas & Mactavish 2002) (Yano et al. 2003). Npff mediates a sympathetic-like increase in blood pressure, heart rate, and contractility when centrally or peripherally administered (Allard et al. 1995; Jhamandas & Goncharuk 2013). It is also implicated in modulating morphine and ketamine analgesia (Elhabazi et al. 2012; Tan et al. 2015). Taken together, this cohort of neurons appear to be mediating a combination of anesthetic and stress-related responses in the DMV. That gene expression changes in the DMV less than three hours after 15 minutes of transient ischemia might be detected is a demonstration of how responsive the DMV is to peripheral afferent signals.

Summary of literature for interpreting the differential expression of mircoRNAs during LAD ligation with and without RIPC

miR-495 has been implicated in many inflammatory processes through its regulation of NOD2, where NOD2 can affect the expression of other pro-inflammatory cytokines and has also been shown to suppress NLRP3 inflammasome signaling, providing protection to cardiac microvasculature following ischemia/reperfusion injury (Cheng et al. 2017). Dysregulation of miR-495 has been implicated in processes involving motor neurons, such as ALS, and, interestingly, has been implicated in the downregulation of the GluA2 subunit of the Gria2 receptor (Capauto et al. 2018). This is in contrast to the stimulatory effects on Gria2 suggested to be associated with miR-218a. miR-218a, which has been found to be particularly enriched in neurites, stimulates the translation of the GluA2 subunit of the Gria2 AMPA glutamate receptor and has also been shown to target the RE-1 silencing transcription factor (REST), a transcriptional repressor of GluA2. Therefore, miR-218a enhances the strength of excitatory synapses by inhibiting the REST complex as well as enhances excitability through stimulation of GluA2 (Rocchi et al. 2019). Taken together, the downregulation in LAD alone and subsequent recovery of both miR-218a and miR-495 with RIPC preceding LAD shows careful control of neuron excitability as well as inflammatory processes, and suggests that treatment with miR-mimics could provide some level of cardiac protection prior to LAD. miR-183 targets a wide range of genes that have both positive and negative effects on neuronal processes and inflammatory processes (Dambal et al. 2015). In particular, miR-183 has been found to modulate genes related to neuron hyperexcitability and inflammation including Nav1.3, Bdnf, and Trpv1, a voltage-gated sodium channel, a neuroprotective agent, and a non-selective ion channel (Ureña-Peralta et al. 2018). This is consistent with upregulation of miR-183 in the DMV following LAD, as well as its downregulation as a protective effect provided by RIPC.

Effects of persistent cardiac ischemia on gene co-expression network topology

Gene co-expression networks were generated for each of the experimental groups using Pearson correlations and filtered using a q value cut-off of $q<10^{-3}$. The unique edges for each of the networks generated are given in Figure S18 with the connectivity in any of the experimental groups being excluded from the full network for each group given in Figures S19, S20, and S21. Genes with no connectivity in any of the networks based upon the cut-off criteria mentioned previously were excluded from all the network figures. It is clear from these networks that the LAD-1 group has the greatest overall connectivity with over half of all edges being unique for that group. Many of these unique edges are the negative correlation between genes in GC-1 and GC-5. Also of note are unique edges of interconnectivity within GC-1 and GC-5 respectively. The notable unique edges of the Sham 1 week group include several negative correlations with Foxo4, Camk2, and Drd4 in GC-3 with genes in GC-1. Also, there are several unique positive correlations

to genes in GC-5 from other GCs as well as some unique interconnectivity within GC-5. The Sham-3 group is most notable for its very sparse connectivity. The LAD-3 group has much lower connectivity than either of the 1 week groups and also less unique edges. Most surprising is the emergence of Pax4a as a unique hub gene in its relationship to many GC-5 genes.

Within each of the treatment cohorts, there are several highly connected genes as is the baseline expectation for a biological network, suspected to have scale-free topology(Barabási 2009). Overall, a few genes are highly connected across most of the cohorts, most notable being Cacna1b that is a member of the top five connected genes in each cohort. Cebpd is highly connected in the LAD-1 group and the LAD-3 group, but with many unique edges in the LAD-1 group. This suggests a role of Cepbd in mediating gene expression in the acute response to cardiovascular injury that persists to some extent beyond the acute phase.

Although the connectivity among the measured genes in the LAD-3 group is not as high as the either of the 1 week groups, there are still some inferences to be made from what is uniquely connected. There are three genes of interest here: IL6r, Ltb4r, and Pax4a. While IL-6 is a well-described pro-inflammatory cytokine, this is not to suppose that the effects of IL-6 binding its receptor in all contexts serves to increase inflammation. In neurons, the IL-6 receptor mediates anti-apoptotic signaling and can serve to protect against reactive oxygen species associated with metabolic stressors (Thier et al. 1999; März et al. 1998). The levels of IL6r do not differ significantly between LAD-1, LAD-3 and Sham-3 (it is suppressed in Sham-1 relative to the others). However, high connectivity of IL6r in the LAD-3 group suggests that there is some signaling that is mediated by the protein product that is having an effect on expression of other genes, including Ltb4r and the pacemaker contributing channels Kcna1 and Hcn2. Similarly, the leukotriene B4 receptor is a canonical mediator of inflammatory processes, but has a different role in neurons of the central nervous system, promoting neurogenesis and neural differentiation from progenitor stem cells (Wada et al. 2006). There is a great deal of evidence for a similar role for the IL-6 receptor in neurons of the CNS (Islam et al. 2009). It may be possible that a cohort of genes associated with IL6r and Ltb4r expression are related to neural progenitor phenotype if stem cells were under consideration. However, these cells are neurons and were selected based upon strong NeuN protein expression as evidenced through immunofluorescence. Since NeuN is only expressed in neural progenitor cells after the neuron “fate” has been determined (Gusel’nikova & Korzhevskiy 2015), it is highly unlikely that any stem cells of pre-neuronal phenotype were selected for this study if any do in fact exist in the DMV of adult rats. Therefore, it is possible that reactivation of some of the differentiating cell programming is used to regress the terminally differentiated neurons and permit them to undergo a phenotype shift. The third unique hub gene may give a clue to the nature of this phenotype shift, since Pax4a mediates differentiation into a neurosecretory phenotype both in the central nervous system as well as for the specialized secretory cells in the pancreas (Schonhoff et al. 2004; Biason-Lauber et al. 2005). This is supported by upregulation of several genes in GC-5 that are coordinated by the three hub genes, IL6r, Ltb4r, and Pax4a. Many genes in GC-5 are suggestive of a neurosecretory phenotype, including several ion channels (Cacna1d, Kcna1, Hcn2) (Goldberg et al. 2012; Cooper et al. 2015), neuropeptide signaling (Sstr2, Sstr5, Cck, Hcrtr1, Hcrtr2), and GABA receptors (Gabra1, Gabra2, Gabrb1, Gabrb2) (Gameiro et al. 2005). While not verified directly in this work here, many of the genes that are well-connected in the unique LAD-3 network are those under the influence of the repressor element 1 silencing transcription factor (REST) complex, an essential regulator of a neurosecretory phenotype (Bruce et al. 2006; Mieda et al. 1997; Wood et al. 1996). More work is needed to consider the possibility that repression of REST plays a role in the phenotype shift toward a neurosecretory phenotype.

Effects of persistent cardiac ischemia on right versus left DMV neuronal gene expression

A differential expression analysis was performed using a nested ANOVA to account for multiple measurements within each animal. While several genes did show differential expression between the sham and LAD groups at both 1 week and 3 weeks post-surgery, these significant differences can be best explained by changes on either the right side or left side only (Figure S15). Of the six genes found to be significantly different, only Camk2 showed the bilateral difference of decreased expression in LAD-1 compared with Sham-1. This overall lower expression seems to be due to a loss of a bimodal distribution that is present in the sham condition. The other five genes from this study whose expression levels differed showed a pronounced effect on only the right or the left side (Figure S15). Only Sst (somatostatin precursor) was shown to change only on the left side of the DMV with the largely increased expression reaching significance at the 3 week time point. The right side did show increased Sst expression at 3 weeks as well, but it did not rise to the level of statistical significance due to the more broad distribution of expression values. Four genes showed significant changes on the right side of the DMV: Hrh2, Npff, Fosb, and Cacna1e. The largest effect size here is the diminished expression of Npff in LAD-1 compared with Sham-1. Fosb showed decreased expression on the right only at the 1 week time point and Cacna1e showed increased expression only at the 3 week time point. Hrh2 expression had an interesting pattern, wherein the expression on the right in the sham condition at both time points was much higher than the left, which was at a comparable level to both sides in the LAD condition at both time points. The LAD ligation surgery was associated with a shift down to left sided levels at both the 1 week and 3 week points.

References

Abegaz, B. et al., 2013. Cardiovascular role of angiotensin type1A receptors in the nucleus of the solitary tract of mice. *Cardiovascular Research*, 100(2), pp.181–191.

Adler, A. et al., 2013. Different correlation patterns of cholinergic and GABAergic interneurons with striatal projection neurons. *Frontiers in Systems Neuroscience*, 7, p.47.

Agarwal, V. et al., 2015. Predicting effective microRNA target sites in mammalian mRNAs. *eLife*, 4.

Allard, M. et al., 1995. Mechanisms underlying the cardiovascular responses to peripheral administration of NPFF in the rat. *The Journal of Pharmacology and Experimental Therapeutics*, 274(1), pp.577–583.

Andrade, A. et al., 2016. A rare schizophrenia risk variant of CACNA1I disrupts CaV3.3 channel activity. *Scientific Reports*, 6, p.34233.

Balaban, C.D. & Beryozkin, G., 1994. Vestibular nucleus projections to nucleus tractus solitarius and the dorsal motor nucleus of the vagus nerve: potential substrates for vestibulo-autonomic interactions. *Experimental Brain Research*, 98(2), pp.200–212.

Barabási, A.-L., 2009. Scale-free networks: a decade and beyond. *Science*, 325(5939), pp.412–413.

Basalay, M.V. et al., 2016. Glucagon-like peptide-1 (GLP-1) mediates cardioprotection by remote ischaemic conditioning. *Cardiovascular Research*, 112(3), pp.669–676.

Bealer, S.L., 1999. Central neuronal histamine contributes to cardiovascular regulation. *Physiology*, 14(3), pp.100–105.

Betel, D. et al., 2008. The microRNA.org resource: targets and expression. *Nucleic Acids Research*, 36(Database issue), pp.D149–53.

Biason-Lauber, A. et al., 2005. Association of childhood type 1 diabetes mellitus with a variant of PAX4: possible link to beta cell regenerative capacity. *Diabetologia*, 48(5), pp.900–905.

Black, P.H., 2002. Stress and the inflammatory response: a review of neurogenic inflammation. *Brain, Behavior, and Immunity*, 16(6), pp.622–653.

Boulland, J.-L. et al., 2009. Vesicular glutamate and GABA transporters sort to distinct sets of vesicles in a population of presynaptic terminals. *Cerebral Cortex*, 19(1), pp.241–248.

Braak, H. et al., 1999. Extensive axonal Lewy neurites in Parkinson's disease: a novel pathological feature revealed by alpha-synuclein immunocytochemistry. *Neuroscience Letters*, 265(1), pp.67–69.

Bruce, A.W. et al., 2006. The transcriptional repressor REST is a critical regulator of the neurosecretory phenotype. *Journal of Neurochemistry*, 98(6), pp.1828–1840.

Le Brun, I. et al., 2008. Differential expression of Nk1 and NK3 neurokinin receptors in neurons of the nucleus tractus solitarius and the dorsal vagal motor nucleus of the rat and mouse. *Neuroscience*, 152(1), pp.56–64.

Bullitt, E., 1990. Expression of c-fos-like protein as a marker for neuronal activity following noxious stimulation in the rat. *The Journal of Comparative Neurology*, 296(4), pp.517–530.

Burke, R.E., Dauer, W.T. & Vonsattel, J.P.G., 2008. A critical evaluation of the Braak staging scheme for Parkinson's disease. *Annals of Neurology*, 64(5), pp.485–491.

Bylund, D.B., 1992. Subtypes of alpha 1- and alpha 2-adrenergic receptors. *The FASEB Journal*, 6(3), pp.832–839.

Cailotto, C. et al., 2014. Neuro-anatomical evidence indicating indirect modulation of macrophages by vagal efferents in the intestine but not in the spleen. *Plos One*, 9(1), p.e87785.

Cain, S.M. & Snutch, T.P., 2013. T-type calcium channels in burst-firing, network synchrony, and epilepsy. *Biochimica et Biophysica Acta*, 1828(7), pp.1572–1578.

Capauto, D. et al., 2018. A Regulatory Circuitry Between Gria2, miR-409, and miR-495 Is Affected by ALS FUS Mutation in ESC-Derived Motor Neurons. *Molecular Neurobiology*, 55(10), pp.7635–7651.

Cheah, C.S. et al., 2012. Specific deletion of NaV1.1 sodium channels in inhibitory interneurons causes seizures and premature death in a mouse model of Dravet syndrome. *Proceedings of the National Academy of Sciences of the United States of America*, 109(36), pp.14646–14651.

Cheng, Y.-F. et al., 2017. Cardioprotection induced in a mouse model of neuropathic pain via anterior nucleus of paraventricular thalamus. *Nature Communications*, 8(1), p.826.

Cheng, Z. et al., 1999. Projections of the dorsal motor nucleus of the vagus to cardiac ganglia of rat atria: an anterograde tracing study. *The Journal of Comparative Neurology*, 410(2), pp.320–341.

Cheng, Z. et al., 1997. Vagal afferent innervation of the atria of the rat heart reconstructed with confocal microscopy. *The Journal of Comparative Neurology*, 381(1), pp.1–17.

Cooper, G. et al., 2015. Functional segregation of voltage-activated calcium channels in motoneurons of the dorsal motor nucleus of the vagus. *Journal of Neurophysiology*, 114(3), pp.1513–1520.

Costello, J.F., Brown, M.R. & Gray, T.S., 1991. Bombesin immunoreactive neurons in the hypothalamic paraventricular nucleus innervate the dorsal vagal complex in the rat. *Brain Research*, 542(1), pp.77–82.

Dambal, S. et al., 2015. The microRNA-183 cluster: the family that plays together stays together. *Nucleic Acids Research*, 43(15), pp.7173–7188.

Danik, M. et al., 2005. Frequent coexpression of the vesicular glutamate transporter 1 and 2 genes, as well as coexpression with genes for choline acetyltransferase or glutamic acid decarboxylase in neurons of rat brain. *Journal of Neuroscience Research*, 81(4), pp.506–521.

DeCicco, D. et al., 2015. MicroRNA network changes in the brain stem underlie the development of hypertension. *Physiological Genomics*, 47(9), pp.388–399.

Deng, C., Weston-Green, K. & Huang, X.-F., 2010. The role of histaminergic H1 and H3 receptors in food intake: a mechanism for atypical antipsychotic-induced weight gain? *Progress in Neuro-Psychopharmacology & Biological Psychiatry*, 34(1), pp.1–4.

DePuy, S.D. et al., 2013. Glutamatergic neurotransmission between the C1 neurons and the parasympathetic preganglionic neurons of the dorsal motor nucleus of the vagus. *The Journal of Neuroscience*, 33(4), pp.1486–1497.

Dickson, D.W. et al., 2010. Evidence in favor of Braak staging of Parkinson's disease. *Movement Disorders*, 25 Suppl 1, pp.S78–82.

Dweep, H. et al., 2011. miRWalk--database: prediction of possible miRNA binding sites by "walking" the genes of three genomes. *Journal of Biomedical Informatics*, 44(5), pp.839–847.

Elhabazi, K. et al., 2012. Involvement of neuropeptide FF receptors in neuroadaptive responses to acute and chronic opiate treatments. *British Journal of Pharmacology*, 165(2), pp.424–435.

Fang, Z. & Rajewsky, N., 2011. The impact of miRNA target sites in coding sequences and in 3'UTRs. *Plos One*, 6(3), p.e18067.

Fattorini, G. et al., 2009. VGLUT1 and VGAT are sorted to the same population of synaptic vesicles in subsets of cortical axon terminals. *Journal of Neurochemistry*, 110(5), pp.1538–1546.

Fong, A.Y. et al., 2005. Immunohistochemical localization of GAD67-expressing neurons and processes in the rat brainstem: subregional distribution in the nucleus tractus solitarius. *The Journal of Comparative Neurology*, 493(2), pp.274–290.

Fox, E.A. & Powley, T.L., 1985. Longitudinal columnar organization within the dorsal motor nucleus represents separate branches of the abdominal vagus. *Brain Research*, 341(2), pp.269–282.

Fox, E.A. & Powley, T.L., 1992. Morphology of identified preganglionic neurons in the dorsal motor nucleus of the vagus. *The Journal of Comparative Neurology*, 322(1), pp.79–98.

Gameiro, A. et al., 2005. The neurotransmitters glycine and GABA stimulate glucagon-like peptide-1 release from the GLUTag cell line. *The Journal of Physiology*, 569(Pt 3), pp.761–772.

Gao, H. et al., 2009. Morphological and electrophysiological features of motor neurons and putative interneurons in the dorsal vagal complex of rats and mice. *Brain Research*, 1291, pp.40–52.

Garrick, T. et al., 1994. Raphe pallidus stimulation increases gastric contractility via TRH projections to the dorsal vagal complex in rats. *Brain Research*, 636(2), pp.343–347.

Goldberg, J.A. et al., 2012. Calcium entry induces mitochondrial oxidant stress in vagal neurons at risk in Parkinson’s disease. *Nature Neuroscience*, 15(10), pp.1414–1421.

Gray, T.S. & Magnuson, D.J., 1987. Neuropeptide neuronal efferents from the bed nucleus of the stria terminalis and central amygdaloid nucleus to the dorsal vagal complex in the rat. *The Journal of Comparative Neurology*, 262(3), pp.365–374.

Green, M., 1978. Histamine in the central nervous system. *Proceedings of the Western Pharmacology Society*, 21, pp.337–339.

Greene, J.G., Dingledine, R. & Greenamyre, J.T., 2005. Gene expression profiling of rat midbrain dopamine neurons: implications for selective vulnerability in parkinsonism. *Neurobiology of Disease*, 18(1), pp.19–31.

Guo, J.J. et al., 2001. Catecholaminergic neurons in rat dorsal motor nucleus of vagus project selectively to gastric corpus. *American Journal of Physiology. Gastrointestinal and Liver Physiology*, 280(3), pp.G361–7.

Gusel’nikova, V.V. & Korzhevskiy, D.E., 2015. Neun as a neuronal nuclear antigen and neuron differentiation marker. *Acta naturae*, 7(2), pp.42–47.

Gutiérrez, R., 2005. The dual glutamatergic-GABAergic phenotype of hippocampal granule cells. *Trends in Neurosciences*, 28(6), pp.297–303.

Hall, J.E., 2014. Neuroendocrine control of the menstrual cycle. In *Yen & jaffe’s reproductive endocrinology*. Elsevier, pp. 141–156.e4.

Herbert, H. & Saper, C.B., 1990. Cholecystokinin-, galanin-, and corticotropin-releasing factor-like immunoreactive projections from the nucleus of the solitary tract to the parabrachial nucleus in the rat. *The Journal of Comparative Neurology*, 293(4), pp.581–598.

Higgins, G.A. & Schwaber, J.S., 1983. Somatostatinergic projections from the central nucleus of the amygdala to the vagal nuclei. *Peptides*, 4(5), pp.657–662.

Hyde, T.M., Knable, M.B. & Murray, A.M., 1996. Distribution of dopamine D1-D4 receptor subtypes in human dorsal vagal complex. *Synapse*, 24(3), pp.224–232.

Islam, O. et al., 2009. Interleukin-6 and neural stem cells: more than gliogenesis. *Molecular Biology of the Cell*, 20(1), pp.188–199.

Jacquin, M.F. et al., 1982. Trigeminal primary afferents project bilaterally to dorsal horn and ipsilaterally to cerebellum, reticular formation, and cuneate, solitary, supratrigeminal and vagal nuclei. *Brain Research*, 246(2), pp.285–291.

Jansen, A. et al., 2000. GABA C receptors in neuroendocrine gut cells: a new GABA-binding site in the gut. *Pflugers Archiv European Journal of Physiology*, 441(2-3), pp.294–300.

Jarvie, B.C. & Hentges, S.T., 2012. Expression of GABAergic and glutamatergic phenotypic markers in hypothalamic proopiomelanocortin neurons. *The Journal of Comparative Neurology*, 520(17), pp.3863–3876.

Jarvinen, M.K. & Powley, T.L., 1999. Dorsal motor nucleus of the vagus neurons: A multivariate taxonomy. *Journal of Comparative Neurology*.

Jhamandas, J.H. & Goncharuk, V., 2013. Role of neuropeptide FF in central cardiovascular and neuroendocrine regulation. *Frontiers in endocrinology*, 4, p.8.

Jhamandas, J.H. & Mactavish, D., 2002. Central administration of neuropeptide FF (NPFF) causes increased neuronal activation and up-regulation of NPFF gene expression in the rat brainstem. *The Journal of Comparative Neurology*, 447(3), pp.300–307.

Kawashima, T., Okuno, H. & Bito, H., 2014. A new era for functional labeling of neurons: activity-dependent promoters have come of age. *Frontiers in Neural Circuits*, 8, p.37.

Kim, H. et al., 2003. Expression of neuropeptide Y and cholecystokinin in the rat brain by chronic mild stress. *Brain Research*, 983(1-2), pp.201–208.

Kuteeva, E. et al., 2008. Differential role of galanin receptors in the regulation of depression-like behavior and monoamine/stress-related genes at the cell body level. *Neuropsychopharmacology*, 33(11), pp.2573–2585.

Lee, S.E. et al., 2014. Rebound burst firing in the reticular thalamus is not essential for pharmacological absence seizures in mice. *Proceedings of the National Academy of Sciences of the United States of America*, 111(32), pp.11828–11833.

Liu, X.-B. & Murray, K.D., 2012. Neuronal excitability and calcium/calmodulin-dependent protein kinase type II: location, location, location. *Epilepsia*, 53 Suppl 1, pp.45–52.

Loewy, A.D., Franklin, M.F. & Haxhiu, M.A., 1994. CNS monoamine cell groups projecting to pancreatic vagal motor neurons: a transneuronal labeling study using pseudorabies virus. *Brain Research*, 638(1-2), pp.248–260.

Loher, P. & Rigoutsos, I., 2012. Interactive exploration of RNA22 microRNA target predictions. *Bioinformatics*, 28(24), pp.3322–3323.

März, P. et al., 1998. Sympathetic neurons can produce and respond to interleukin 6. *Proceedings of the National Academy of Sciences of the United States of America*, 95(6), pp.3251–3256.

Mastitskaya, S. et al., 2012. Cardioprotection evoked by remote ischaemic preconditioning is critically dependent on the activity of vagal pre-ganglionic neurones. *Cardiovascular Research*, 95(4), pp.487–494.

McCann, M.J. & Rogers, R.C., 1990. Oxytocin excites gastric-related neurones in rat dorsal vagal complex. *The Journal of Physiology*, 428, pp.95–108.

Mieda, M., Haga, T. & Saffen, D.W., 1997. Expression of the rat m4 muscarinic acetylcholine receptor gene is regulated by the neuron-restrictive silencer element/repressor element 1. *The Journal of Biological Chemistry*, 272(9), pp.5854–5860.

Mikoshiba, K., 2007. IP3 receptor/Ca2+ channel: from discovery to new signaling concepts. *Journal of Neurochemistry*, 102(5), pp.1426–1446.

O'Malley, D.M. & Masland, R.H., 1989. Co-release of acetylcholine and gamma-aminobutyric acid by a retinal neuron. *Proceedings of the National Academy of Sciences of the United States of America*, 86(9), pp.3414–3418.

Ogura, Y. et al., 2012. Therapeutic impact of follistatin-like 1 on myocardial ischemic injury in preclinical models. *Circulation*, 126(14), pp.1728–1738.

Ottem, E.N. et al., 2004. Dual-phenotype GABA/glutamate neurons in adult preoptic area: sexual dimorphism and function. *The Journal of Neuroscience*, 24(37), pp.8097–8105.

Palkovits, M. et al., 1986. Innervation of the nucleus of the solitary tract and the dorsal vagal nucleus by thyrotropin-releasing hormone-containing raphe neurons. *Brain Research*, 373(1-2), pp.246–251.

Papay, R. et al., 2002. Mice expressing the alpha(1B)-adrenergic receptor induces a synucleinopathy with excessive tyrosine nitration but decreased phosphorylation. *Journal of Neurochemistry*, 83(3), pp.623–634.

Park, J. et al., 2014. Inputs drive cell phenotype variability. *Genome Research*, 24(6), pp.930–941.

Perez-Reyes, E. et al., 1998. Molecular characterization of a neuronal low-voltage-activated T-type calcium channel. *Nature*, 391(6670), pp.896–900.

Poole, S.L., Lewis, D.I. & Deuchars, S.A., 2008. Histamine depolarizes neurons in the dorsal vagal complex. *Neuroscience Letters*, 432(1), pp.19–24.

Rinaman, L., 1999. Interoceptive stress activates glucagon-like peptide-1 neurons that project to the hypothalamus. *The American Journal of Physiology*, 277(2), pp.R582–90.

Rinaman, L., 1998. Oxytocinergic inputs to the nucleus of the solitary tract and dorsal motor nucleus of the vagus in neonatal rats. *Journal of Comparative Neurology*.

Rinaman, L. et al., 1989. Ultrastructural demonstration of a gastric monosynaptic vagal circuit in the nucleus of the solitary tract in rat. *The Journal of Neuroscience*, 9(6), pp.1985–1996.

Rocchi, A. et al., 2019. Neurite-Enriched MicroRNA-218 Stimulates Translation of the GluA2 Subunit and Increases Excitatory Synaptic Strength. *Molecular Neurobiology*, 56(8), pp.5701–5714.

Rogers, G.J. et al., 2011. Electrical activity-triggered glucagon-like peptide-1 secretion from primary murine L-cells. *The Journal of Physiology*, 589(Pt 5), pp.1081–1093.

Saper, C.B. et al., 1989. Brain natriuretic peptides: differential localization of a new family of neuropeptides. *Neuroscience Letters*, 96(1), pp.29–34.

Schonhoff, S.E., Giel-Moloney, M. & Leiter, A.B., 2004. Minireview: Development and differentiation of gut endocrine cells. *Endocrinology*, 145(6), pp.2639–2644.

Schwaber, J.S. et al., 1982. Amygdaloid and basal forebrain direct connections with the nucleus of the solitary tract and the dorsal motor nucleus. *The Journal of Neuroscience*, 2(10), pp.1424–1438.

Schwörer, H., Racké, K. & Kilbinger, H., 1989. GABA receptors are involved in the modulation of the release of 5-hydroxytryptamine from the vascularly perfused small intestine of the guinea-pig. *European Journal of Pharmacology*, 165(1), pp.29–37.

Snutch, T.P. et al., 2013. Molecular Properties of Voltage-Gated Calcium Channels - Madame Curie Bioscience Database - NCBI Bookshelf.

Standish, A. et al., 1995. Central neuronal circuit innervating the rat heart defined by transneuronal transport of pseudorabies virus. *The Journal of Neuroscience*, 15(3 Pt 1), pp.1998–2012.

Taché, Y., Yang, H. & Kaneko, H., 1995. Caudal raphe-dorsal vagal complex peptidergic projections: role in gastric vagal control. *Peptides*, 16(3), pp.431–435.

Tan, S. et al., 2015. Chronic effects of ketamine on gene expression changes in neurotransmitter receptors and regulators-A PCR-array study. *Molecular & Cellular Toxicology*, 11(4), pp.395–400.

Tan, Z. et al., 2004. Galanin inhibits gut-related vagal neurons in rats. *Journal of Neurophysiology*, 91(5), pp.2330–2343.

Thier, M. et al., 1999. Interleukin-6 (IL-6) and its soluble receptor support survival of sensory neurons. *Journal of Neuroscience Research*.

Trapp, S. & Hisadome, K., 2011. Glucagon-like peptide 1 and the brain: central actions-central sources? *Autonomic Neuroscience: Basic & Clinical*, 161(1-2), pp.14–19.

Ureña-Peralta, J.R. et al., 2018. Deep sequencing and miRNA profiles in alcohol-induced neuroinflammation and the TLR4 response in mice cerebral cortex. *Scientific Reports*, 8(1), p.15913.

Wada, K. et al., 2006. Leukotriene B4 and lipoxin A4 are regulatory signals for neural stem cell proliferation and differentiation. *The FASEB Journal*, 20(11), pp.1785–1792.

Wang, X. et al., 2014. Optogenetic stimulation of locus ceruleus neurons augments inhibitory transmission to parasympathetic cardiac vagal neurons via activation of brainstem α1 and β1 receptors. *The Journal of Neuroscience*, 34(18), pp.6182–6189.

Wei, K. et al., 2015. Epicardial FSTL1 reconstitution regenerates the adult mammalian heart. *Nature*, 525(7570), pp.479–485.

Wood, I.C., Roopra, A. & Buckley, N.J., 1996. Neural specific expression of the m4 muscarinic acetylcholine receptor gene is mediated by a RE1/NRSE-type silencing element. *The Journal of Biological Chemistry*, 271(24), pp.14221–14225.

Xu, X. et al., 2001. Promotive effects of gaba on acid secretion from isolated mouse stomach In vitro. *Dong wu xue bao. [Acta zoologica Sinica]*.

Yang, Y. et al., 2018. Ketamine blocks bursting in the lateral habenula to rapidly relieve depression. *Nature*, 554(7692), pp.317–322.

Yano, T. et al., 2003. Localization and neuronal response of RFamide related peptides in the rat central nervous system. *Brain Research*, 982(2), pp.156–167.

Zander, J.-F. et al., 2010. Synaptic and vesicular coexistence of VGLUT and VGAT in selected excitatory and inhibitory synapses. *The Journal of Neuroscience*, 30(22), pp.7634–7645.

Zheng, H., Patterson, L.M. & Berthoud, H.-R., 2005. Orexin-A projections to the caudal medulla and orexin-induced c-Fos expression, food intake, and autonomic function. *The Journal of Comparative Neurology*, 485(2), pp.127–142.

Zheng, Z.H., Dietrichs, E. & Walberg, F., 1982. Cerebellar afferent fibres from the dorsal motor vagal nucleus in the cat. *Neuroscience Letters*, 32(2), pp.113–118.
